# Supplementary material for: Subcellular and regional localization of mRNA translation in midbrain dopamine neurons
Source: Cell Rep. Author manuscript; Available in PMC 2022 Feb 15. (PMC8844886; doi:10.1016/j.celrep.2021.110208)
Supplement: 9 [file NIHMS1771089-supplement-9.pdf]

Cell Reports

Article

Subcellular and regional localization of mRNA translation in midbrain dopamine neurons

Graphical abstract

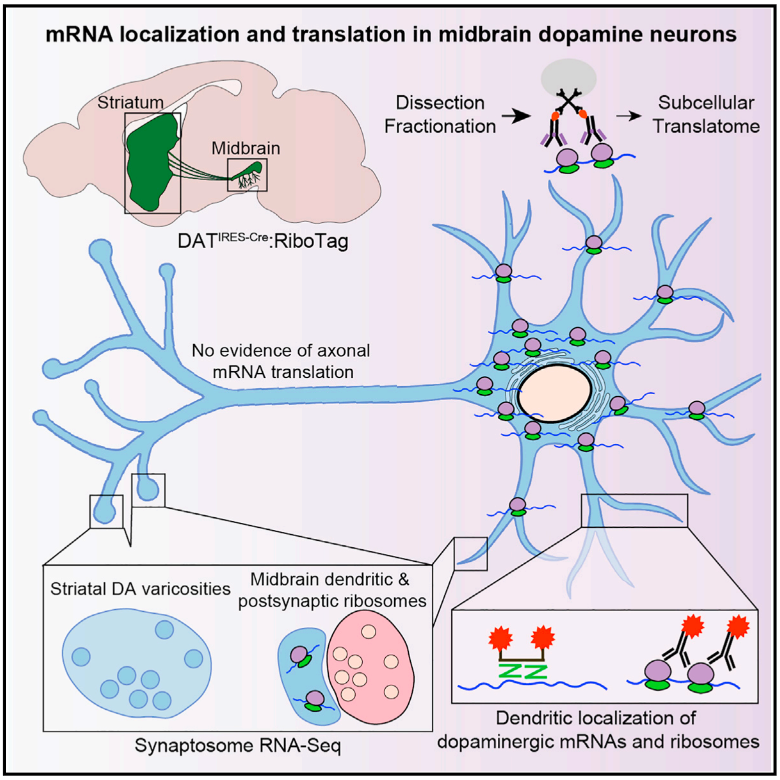

Authors

Benjamin D. Hobson, Linghao Kong, Maria Florencia Angelo, ..., Etienne Herzog, David Sulzer, Peter A. Sims

Correspondence

etienne.herzog@u-bordeaux.fr (E.H.),
ds43@cumc.columbia.edu (D.S.),
pas2182@cumc.columbia.edu (P.A.S.)

In brief

Local translation regulates the subcellular proteome in neurons but has not been characterized in midbrain dopamine neurons, cells with large dendrites and axonal arborizations. Hobson et al. investigate messenger RNA localization and translation in midbrain dopamine neurons in the mouse brain, finding ribosomes and dopaminergic mRNAs in dendrites, but not axons.

Highlights

- No evidence of mRNA translation in dopaminergic axons in the mouse brain
- Dopaminergic dendrites contain ribosomes and mRNA encoding DA transmission proteins
- Dendritic localization of mRNAs and exocytic fusion proteins
- Molecular signature of SNr mDA neurons, including *Atp2a3*/SERCA3, an ER calcium pump

Hobson et al., 2022, Cell Reports *38*, 110208
January 11, 2022 © 2021 The Author(s).
https://doi.org/10.1016/j.celrep.2021.110208

Cell Reports

CellPress
OPEN ACCESS

Article

Subcellular and regional localization of mRNA translation in midbrain dopamine neurons

Benjamin D. Hobson,[1,2,6] Linghao Kong,[1] Maria Florencia Angelo,[3,4] Ori J. Lieberman,[2,6,13] Eugene V. Mosharov,[6,8] Etienne Herzog,[3,4,12,*] David Sulzer,[5,6,7,8,11,12,*] and Peter A. Sims[1,9,10,11,12,14,*]
[1]Department of Systems Biology, Columbia University Irving Medical Center, New York 10032, NY, USA
[2]Medical Scientist Training Program, Columbia University Irving Medical Center, New York, NY 10032, USA
[3]Interdisciplinary Institute for Neuroscience, Université de Bordeaux, Bordeaux, France
[4]Interdisciplinary Institute for Neuroscience, CNRS UMR 5297, Bordeaux, France
[5]Department of Neurology, Columbia University Irving Medical Center, New York, NY 10032, USA
[6]Department of Psychiatry, Columbia University Irving Medical Center, New York, NY 10032, USA
[7]Department of Pharmacology, Columbia University Irving Medical Center, New York, NY 10032, USA
[8]Division of Molecular Therapeutics, New York State Psychiatric Institute, New York, NY 10032, USA
[9]Department of Biochemistry & Molecular Biophysics, Columbia University Irving Medical Center, New York, NY 10032, USA
[10]Sulzberger Columbia Genome Center, Columbia University Irving Medical Center, New York, NY 10032, USA
[11]Aligning Science Across Parkinson's (ASAP) Collaborative Research Network, Chevy Chase, MD, USA
[12]Senior author
[13]Present address: Department of Neurology, University of California San Francisco School of Medicine, San Francisco, CA, USA
[14]Lead contact
*Correspondence: etienne.herzog@u-bordeaux.fr (E.H.), ds43@cumc.columbia.edu (D.S.), pas2182@cumc.columbia.edu (P.A.S.)
https://doi.org/10.1016/j.celrep.2021.110208

SUMMARY

Midbrain dopaminergic (mDA) neurons exhibit extensive dendritic and axonal arborizations, but local protein synthesis is not characterized in these neurons. Here, we investigate messenger RNA (mRNA) localization and translation in mDA neuronal axons and dendrites, both of which release dopamine (DA). Using highly sensitive ribosome-bound RNA sequencing and imaging approaches, we find no evidence for mRNA translation in mDA axons. In contrast, mDA neuronal dendrites in the substantia nigra pars reticulata (SNr) contain ribosomes and mRNAs encoding the major components of DA synthesis, release, and reuptake machinery. Surprisingly, we also observe dendritic localization of mRNAs encoding synaptic vesicle-related proteins, including those involved in exocytic fusion. Our results are consistent with a role for local translation in the regulation of DA release from dendrites, but not from axons. Our translatome data define a molecular signature of sparse mDA neurons in the SNr, including the enrichment of *Atp2a3/SERCA3*, an atypical ER calcium pump.

INTRODUCTION

Midbrain dopaminergic (mDA) neurons play critical roles in reward processing, movement control, and cognitive function. Their elaborate cytoarchitecture includes unmyelinated axons that course through the medial forebrain bundle (MFB) to reach basal ganglia and cortical targets (Björklund and Dunnett, 2007). Individual mDA neurons of the murine substantia nigra pars compacta (SNc) exhibit axonal arborizations reaching up to 500 mm in total length that possess 10^4–10^5 presynaptic varicosities (Matsuda et al., 2009). In addition to axonal DA release in the striatum and cortex, mDA neurons release DA within the midbrain (reviewed in Rice and Patel, 2015 and Cheramy et al., 1981), including from ventrally directed dendrites of SNc neurons that can project more than 500 μm into the substantia nigra pars reticulata (SNr) (Geffen et al., 1976; Korf et al., 1976; Tepper et al., 1987). Recent work has begun to identify molecular mechanisms that regulate DA release in the midbrain (Chen and Rice, 2001; Mendez et al., 2011; Robinson et al., 2019; Witkovsky et al., 2009) and striatum (Banerjee et al., 2020; Liu et al., 2018), but it is unclear how mDA neurons localize and maintain DA neurotransmission machinery in both dendritic and axonal compartments.

The subcellular proteome of neurons is regulated in part by local translation. Dendritic protein synthesis plays a critical role in several forms of postsynaptic plasticity (Bradshaw et al., 2003; Cracco et al., 2005; Huber et al., 2000; Kang and Schuman, 1996), and local translation is involved in the developmental pathfinding of axonal growth cones and the regeneration of peripheral axons (reviewed in Crispino et al., 2014; Jung et al., 2012). Recent evidence suggests that local translation occurs in mature central nervous system axons of excitatory and inhibitory neurons (Hafner et al., 2019; Ostroff et al., 2019; Scarnati et al., 2018; Shigeoka et al., 2016; Younts et al., 2016). Less is known about local translation in monoaminergic neurons. Intriguingly, the messenger RNA (mRNA) encoding tyrosine

© 2021 The Author(s).
This is an open access article under the CC BY license (http://creativecommons.org/licenses/by/4.0/).

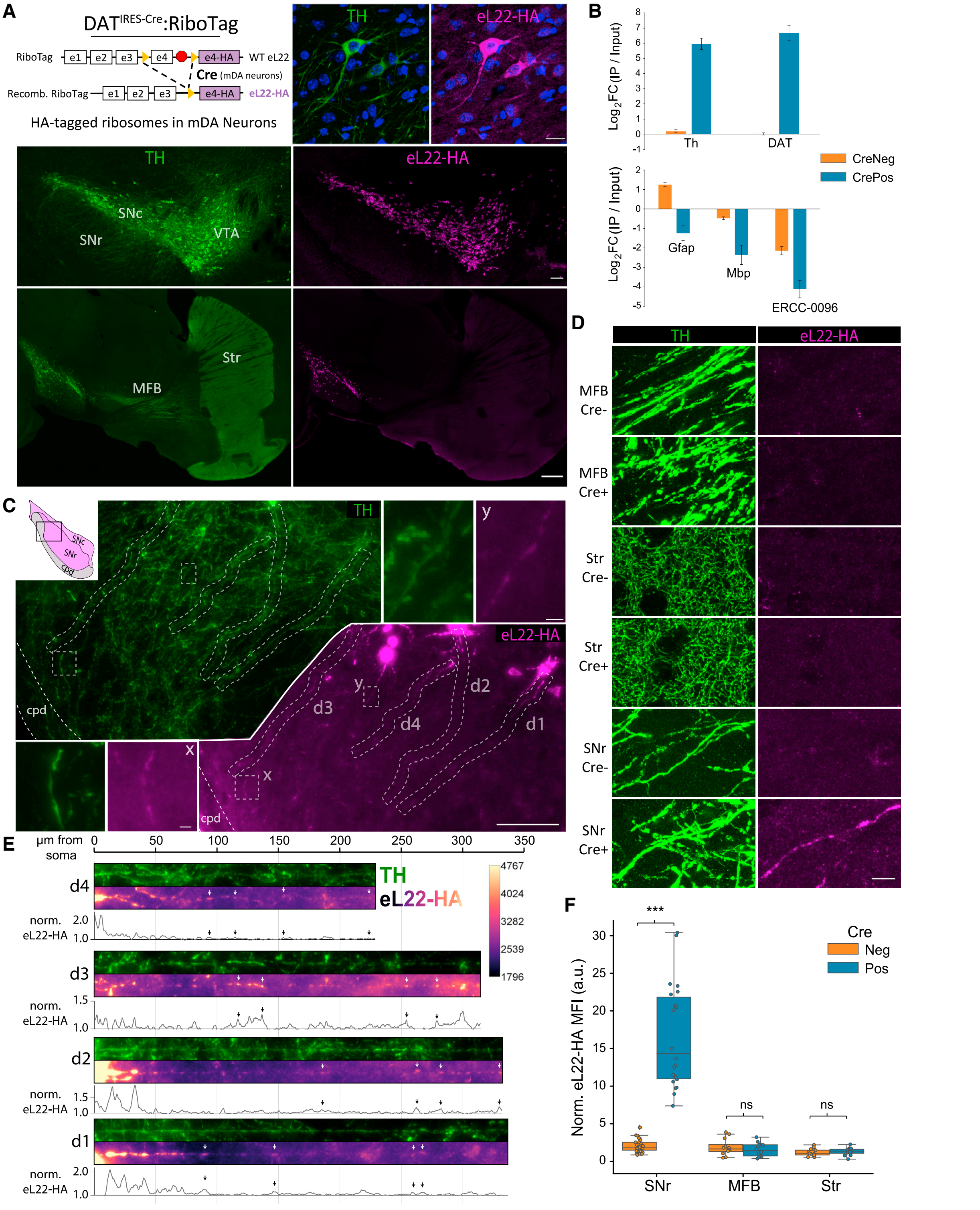

Figure 1. Subcellular distribution of eL22-HA tagged ribosomes in adult (10–14 mo) DAT$^{IRES-Cre}$:RiboTag mice

(A) DAT$^{IRES-Cre}$:RiboTag genetics (*upper left*). TH and eL22-HA IF. *Upper right*: mDA neurons in the SNc, DAPI in blue. Scale bar, 20 μm. *Middle*: Coronal midbrain section. Scale bar, 100 μm. *Lower*: Sagittal section. Scale bar, 500 μm.

(B) qRT-PCR from VM Input and RiboTag IPs from DAT$^{IRES-Cre}$:RiboTag mice (Cre$^+$, n = 7) or Cre$^-$ littermates (n = 5) showing IP/Input enrichment relative to *Actb* (mean ΔΔCq +/− SEM).

(legend continued on next page)

hydroxylase (TH), the rate-limiting enzyme in catecholamine biosynthesis, is localized to axons of sympathetic neurons *in vitro* (Gervasi et al., 2016). Ablation of an axonal localization motif in the 3′ untranslated region of *Th* mRNA decreases axonal TH protein levels and release of norepinephrine (Aschrafi et al., 2017), suggesting that local protein synthesis might regulate neurotransmission in mDA neurons.

Here, we systematically investigate mRNA localization and translation within mDA neurons in the mouse brain. Our results reveal the subcellular organization of protein synthesis in mDA neurons, with implications for the regulation of DA neurotransmission in health and disease.

RESULTS

DAT$^{IRES-Cre}$:RiboTag mice enable visualization and capture of mDA neuronal ribosomes

To study subcellular translation in mDA neurons, we crossed DAT$^{IRES-Cre}$ mice (Bäckman et al., 2006) with RiboTag mice (Sanz et al., 2009) to express HA-tagged eukaryotic ribosomal protein L22 (eL22-HA) specifically in TH$^+$ mDA neurons in the SNc and ventral tegmental area (VTA) (Figures 1A and S1A). Anti-HA immunoprecipitation (IP) of ventral midbrain (VM) polysome extracts from Cre$^+$ RiboTag mice (DAT$^{IRES-Cre/wt}$;RiboTag$^{+/-}$) yielded a more than 64-fold enrichment of mDA neuron-specific mRNAs *Th* and *Slc6a3*/DA transporter (DAT), and depleted glial and mRNAs *Gfap*, *Mbp*, and the soluble polyadenylated spike-in standard *ERCC-0096* (Figure 1B).

Consistent with previous studies of DAT-Cre lines (Bäckman et al., 2006; Lammel et al., 2015; Mingote et al., 2017; Turiault et al., 2007), no expression of eL22-HA was observed in the dorsal striatum, nucleus accumbens, or cortex (Figure 1C). In concordance with recent work using another DAT-Cre line (Papathanou et al., 2019), we found scattered TH$^-$/tdTom$^+$ cells in the lateral septum of DAT$^{IRES-Cre/wt}$ mice crossed to Ai9 tdTomato reporter mice (Figure S1B) (Madisen et al., 2010). However, we found no evidence of eL22-HA expression in the bed nucleus of stria terminalis or lateral septum of DAT$^{IRES-Cre/wt}$;RiboTag$^{+/-}$ mice (Figure S1B). Nonetheless, we removed all tissue medial to the lateral ventricles (see STAR Methods) in our striatal dissections of DAT$^{IRES-Cre/wt}$;RiboTag$^{+/-}$ mice to ensure that striatal eL22-HA was derived from solely from mDA axons.

We analyzed the subcellular distribution of mDA neuronal ribosomes using anti-HA immunofluorescence (IF). eL22-HA labeling was present in soma and proximal dendrites (Figures 1A and S1C), but undetectable within axons in the MFB or striatum (Figure 1A). SNc mDA neurons typically possess three to six long, mostly unbranched dendrites; the largest one or two are directed ventrolaterally into the SNr (Juraska et al., 1977; Prensa and Parent, 2001; Tepper et al., 1987). With tyramide signal amplification (Adams, 1992; Bobrow et al., 1992) (Figures S1D and S1E), eL22-HA labeling was apparent within such dendrites in the SNr (Figures 1C and 1E), even at the distal edge near the cerebral peduncles (cpd; Figure S1F, *lower*). Co-localization with TH IF confirmed that eL22-HA clusters were scattered throughout ventral-directed dendrites of SNc mDA neurons (Figures 1C and 1E), which could be distinguished from a few mDA neuronal soma in the SNr (Figure S1F, *upper*). Consistent with a previous study (Brichta et al., 2015), we observed no specific eL22-HA labeling in the MFB or striatum (Figure 1D). Quantification of eL22-HA in Cre$^+$ versus Cre$^-$ (DAT$^{wt/wt}$;RiboTag$^{+/-}$) mice revealed significantly higher fluorescence in TH$^+$ SNr dendrites, but not TH$^+$ axons in the MFB or striatum (Figures 1D and 1F). Assuming that eL22-HA reflects the localization of endogenous ribosomes (Sanz et al., 2009; Shigeoka et al., 2016), these data are consistent with the majority of mDA neuronal ribosomes residing in the soma and proximal dendrites, with lesser abundance in the distal dendrites and exceedingly low levels in axons.

Sensitive, quantitative capture of dopaminergic ribosomes from regional dissections

To identify translating mRNAs in distinct subcellular compartments of mDA neurons, we conducted RiboTag IP on dissections of four regions (Figure 2A): (1) the dorsal and ventral striatum, containing mDA axons, (2) the VTA, containing mDA somata and dendrites, (3) the SNc, containing mDA somata and dendrites, and (4) the SNr, containing a few mDA somata amid a high density of ventral-projecting dendrites of SNc mDA neurons (Figure S1G). Owing to the low yields from axonal RiboTag, we used Cre$^-$ mice to control for non-specific binding (Shigeoka et al., 2016). The total RNA yield from Cre$^-$ IPs was typically 10s–100s of picograms as estimated using qualitative reverse transcriptase polymerase chain reaction (qRT-PCR) for β-actin (*Actb*) (Figure S1I).

We estimated eL22-HA abundance in each dissected region by Western blotting eL22-HA IPs. To estimate the sensitivity of our striatal IP, we included control samples of Cre$^-$ striatal lysates spiked with 1% of VM lysates from Cre$^+$ mice. eL22-HA bands were prominent in VTA and SNc IPs, while faint eL22-HA signal was only visible at high contrast in IPs from the striatum and 1% VM spike-in control (Figures 2B and 2C). Quantification revealed that of all eL22-HA captured, 37.4% was from the SNc, 54.5% from the VTA, 4.13% from the SNr, 2.23% from the striatum, and 1.75% from our 1% VM spike-in control (Figure 2D). Striatal eL22-HA abundance was not significantly different from the 1% VM spike-in control. eL22-HA abundance correlated well with our spike-in control (1% of VM lysate vs. an estimated 1.75% eL22-HA) and the reported distribution of mDA neurons

(C) Ventrolateral SNc and SNr stained for TH and eL22-HA. x, y insets shown in white lines. Dendrites d1-d4 are displayed below in (E). Scale bars, main image, 100 μm; insets, 5 μm. cpd, cerebral peduncle.

(D) TH and eL22-HA staining in the MFB, striatum, and SNr of Cre$^-$/Cre$^+$ RiboTag mice. Scale bar, 10 μm.

(E) Straightened dendritic segments d1-d4 from (C) with eL22-HA intensity normalized to local background and plotted below. Arrows indicate eL22-HA hotspots.

(F) Quantification of eL22-HA signal within TH$^+$ neurites in the MFB (axons), striatum (axons), and SNr (dendrites) of Cre$^-$ and Cre$^+$ mice. Data are background-normalized mean eL22-HA intensity of TH$^+$ pixels within a field of the indicated region (n = 6-10 fields, n = 4 sections, n = 3 mice per each genotype/region). Two-way ANOVA main effects: Region, $F = 74.4$ (2, 100), $p = 1.6^{-20}$; genotype, $F = 117.2$ (1, 100), $p = 1.5 \times 10^{-18}$; and region:fenotype interaction, $F = 68.4$ (2,100), $p = 1.9 \times 10^{-19}$. Tukey's HSD *post hoc* test for Cre$^+$ versus Cre$^-$ staining: SNr (p-adj < 0.001), MFB and Str (both p-adj > 0.9). ***p-adj < 0.001.

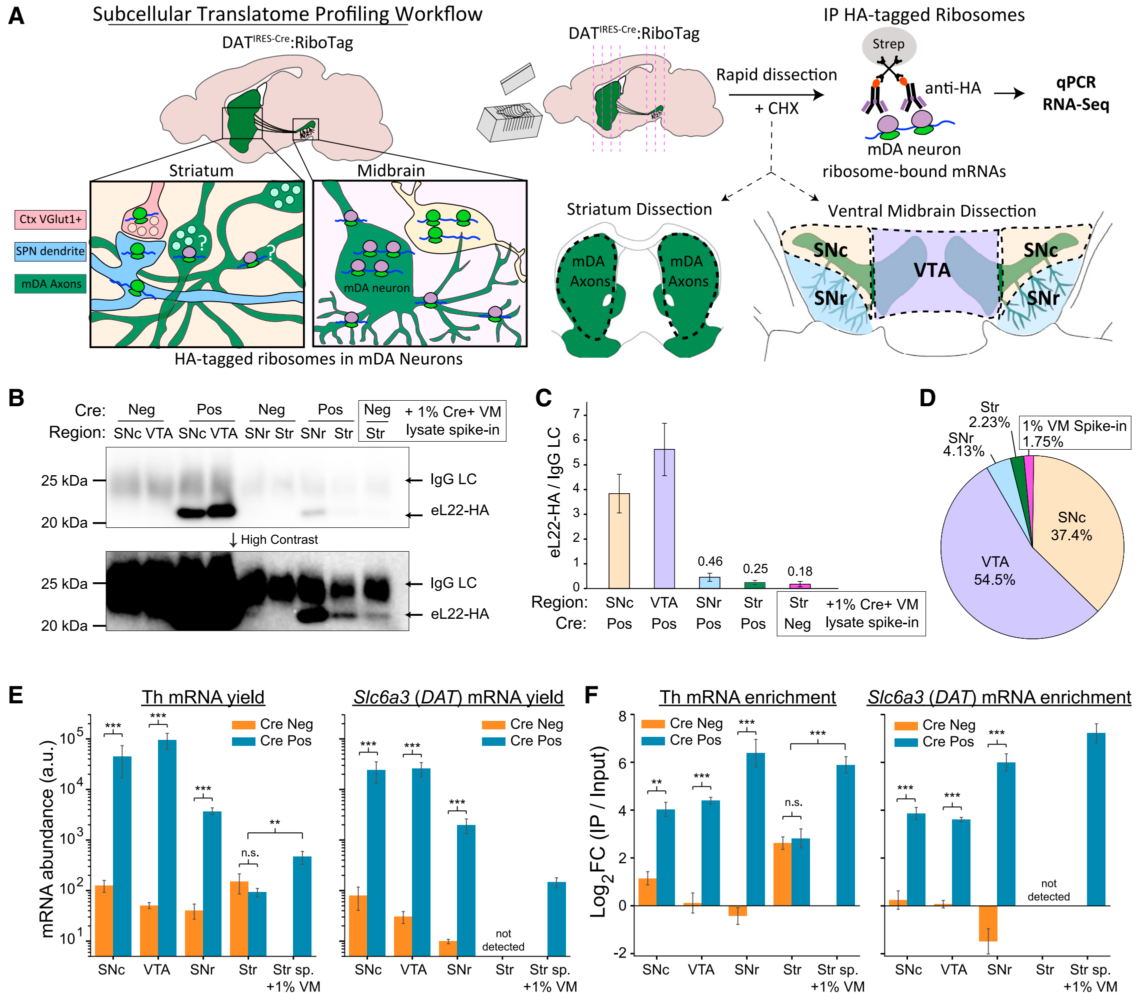

Figure 2. Regional distribution of eL22-HA protein and dopaminergic mRNAs captured by RiboTag IP in adult mice (10–14 mo)

(A) Subcellular translatome profiling workflow.

(B) Western blot of captured eL22-HA from RiboTag IPs. eL22-HA (23 kDa) is detected below IgG light chain (LC, approximately 25 kDa). *Lower:* High contrast reveals bands in striatal samples and 1% VM spike-in.

(C) Quantification of Western blot eL22-HA, normalized to IgG LC with mean ± SEM shown for each region/genotype (n = 3-4).

(D) Fractional abundance of eL22-HA captured in each region, using normalized eL22-HA intensity from (C).

(E and F) qRT-PCR of *Th* and *Slc6a3/DAT* mRNA in RiboTag IPs from each region/genotype (n = 3-4 each). (E) mRNA abundance in arbitrary units (2^{40-Cq}). Mean (A)u. +/− SEM are plotted. For *Th* mRNA, two-way ANOVA main effects: region, F = 17.7 (3, 19), $p = 9.8 \times 10^{-6}$; genotype, F = 168.1 (1, 19), $p = 6.9 \times 10^{-11}$; and region:genotype interaction, F = 20.1 (3, 19), $p = 4.0 \times 10^{-6}$. Tukey's HSD *post hoc* for Cre^{+} versus Cre^{-} samples: ***p-adj<0.001 for SNc, VTA, and SNr, but not for Striatum (p-adj > 0.9). Welch's *t* test for 1%VM/Str_Sp samples versus Cre^{+}/Cre^{-} striatal samples: t(9) = 3.72, p = 0.0062. For DAT mRNA, two-way ANOVA main effects: region, F = 13.0 (2, 15), $p = 5.3 \times 10^{-4}$; genotype, F = 262.5 (1, 15), $p = 6.5 \times 10^{-11}$; and region:genotype interaction, F = 1.46 (2, 15), p = 0.26. DAT mRNA was not detected in striatal samples. Tukey's HSD *post hoc* test for Cre^{+} versus Cre^{-} samples: p-adj < 0.001 for the SNc, VTA, and SNr (***). (F) qRT-PCR for RiboTag IP/Input enrichment relative to *Actb* (mean ΔΔCq +/− SEM). For *Th* mRNA, two-way ANOVA main effects: genotype, F = 154.5 (1, 20), $p = 7.3 \times 10^{-11}$; region, F = 1.97 (3, 20), p = 0.15; and region:genotype interaction, F = 23.4 (3, 20), $p = 9.4 \times 10^{-7}$. Tukey's HSD *post hoc* test for Cre^{+} versus Cre^{-} samples: p-adj < 0.01 for SNc, VTA, and SNr, but not for striatum (p-adj > 0.9). Welch's *t* test for 1%VM/Str_Sp samples versus Cre^{+}/Cre^{-} striatal samples: t(10) = 6.66, p = 0.0009. For *Slc6a3/DAT* mRNA, two-way ANOVA main effects: genotype, F = 269.2 (1, 15), $p = 5.4 \times 10^{-11}$; region, F = 1.96 (3, 15), p = 0.18; and region:genotype interaction, F = 19.1 (2, 15), $p = 7.5 \times 10^{-5}$. Tukey's HSD *post hoc* test for Cre^{+} versus Cre^{-} samples: p-adj < 0.001 for SNc, VTA, and SNr (**p-adj < 0.01, ***p-adj < 0.001).

in C57BL/6J mice (Nelson et al., 1996): approximately 8,000 in the SNc (38% of mDA neurons vs. 37.4% of eL22-HA) and approximately 10,000 in the VTA (47.6% of mDA neurons vs. estimated 54.5% of eL22-HA). The eL22-HA Western blot and histology data support the soma as the major site of protein synthesis in mDA neurons (Palay and Palade, 1955) and are consistent with very low levels or the absence of ribosomes in striatal mDA axons.

qRT-PCR of RiboTag IPs revealed significant Cre-dependent increases in yield and enrichment of *Th* and *Slc6a3/DAT* mRNA in IPs from SNc and VTA (Figures 2E and 2F). We also found a significant Cre-dependent increase in the yield of *Th* and

CellPress
OPEN ACCESS

Table 1. Summary of RiboTag IP capture for eL22-HA, *Th* mRNA, and *Slc6a3/DAT* mRNA

Percentage of total yield

Region	eL22-HA protein	*Th* mRNA	*Slc6a3/DAT* mRNA
SNc	37.4 ± 8.2%	25.5 ± 6.4%	42.0 ± 5.2%
VTA	54.5 ± 8.8%	69.6 ± 5.4%	52.9 ± 4.1%
SNr	4.13 ± 0.77%	4.0 ± 1.2%	4.62 ± 1.02%
Striatum	2.23 ± 0.33%	0.14 ± 0.07%	Undetectable
Striatum +1% VM spike-in	1.75 ± 1.24%	0.78 ± 0.52%	0.51 ± 0.29%

TH (*Th*) mRNA

Region	Log$_2$ (ΔYield) Cre^{+} - Cre^{-}	Tukey HSD (p-adj)	Log$_2$ FC (IP/Input) Cre^{+} - Cre^{-}	Tukey HSD (p-adj)
SNc	7.88 ± 1.16	<0.001	2.88 ± 0.44	0.0013
VTA	10.49 ± 0.73	<0.001	4.28 ± 0.52	<0.001
SNr	6.93 ± 1.03	<0.001	6.82 ± 0.73	<0.001
Striatum	−1.16 ± 1.25	>0.9	0.20 ± 0.50	>0.9
Striatum +1% VM spike-in	1.94 ± 0.94	NA	5.89 ± 0.40	NA

Dopamine transporter (*Slc6a3/DAT*) mRNA

Region	Log$_2$ (ΔYield) Cre^{+}/Cre^{-}	Tukey HSD (p-adj)	Log$_2$ FC (IP/Input) Cre^{+} - Cre^{-}	Tukey HSD (p-adj)
SNc	8.41 ± 1.30	<0.001	3.62 ± 0.51	<0.001
VTA	9.65 ± 0.78	<0.001	3.54 ± 0.22	<0.001
SNr	7.45 ± 0.48	<0.001	7.48 ± 0.78	<0.001
Striatum	NA	NA	NA	NA
Striatum +1% VM spike-in	Cre^{-} undetectable	NA	7.21 ± 0.44 (Cre^{+}) Cre^{-} undetectable	NA

Upper: Mean percentage of total yield ±SEM for each region across all Cre^{+} RiboTag IPs. Data from Figures 2B–2D (eL22-HA protein, n = 3 each region) and Figures 2E and 2F (*Th* and *Slc6a3* mRNA, n = 3-4 each genotype/region).
Lower: Mean log2 differences in yield (*left*) or enrichment (*right*) ± SEM between Cre^{+} and Cre^{-} RiboTag IPs for each region/mRNA. Data and p-adj from Tukey's HSD *post hoc* comparisons are from Figures 2E and 2F (*Th* and *Slc6a3* mRNA, n = 3-4 each genotype/region).

Slc6a3/DAT mRNAs in SNr IPs (Figure 2E) and an enrichment of *Th* and *Slc6a3/DAT* from the SNr that was higher than in the SNc and VTA (Figure 2F). When comparing striatal IPs from Cre^{+} and Cre^{-} mice, we found no significant differences in *Th* mRNA yield or enrichment, and *Slc6a3/DAT* was undetectable in all samples (Figures 2E and 2F). Similar to the SNr, we found a more than 64-fold Cre-dependent enrichment of both *Th* and *Slc6a3/DAT* in our 1% VM spike-in control, despite a low yield (Figures 2E and 2F). The yield and enrichment of *Th* mRNA were significantly higher in our 1% VM spike-in controls than striatal IPs from Cre^{-} and Cre^{+} mice (Figures 2E and 2F). These results demonstrate sensitive, quantitative RiboTag IP from distinct mDA neuron compartments and suggest that *Th* and *Slc6a3/DAT* mRNAs are translated in dopaminergic dendrites in the SNr, but not in striatal mDA axons (Table 1).

Lack of evidence for axonal translation in striatal RiboTag IPs

To identify mRNAs bound to putative axonal ribosomes, we analyzed the content of striatal RiboTag IPs from Cre^{-} and Cre^{+} samples using RNA sequencing (RNA-seq). To accommodate picogram samples, we used a low input, pooled, 3′-end library construction strategy designed for single-cell RNA-seq (scRNA-seq) (Snyder et al., 2019) that includes unique molecular identifiers (UMIs) to mitigate PCR bias and estimate the number of transcripts captured per gene. Developing axons typically have a greater translational capacity than mature axons, which may reflect the downregulation of axonal ribosomes after synaptogenesis (Costa et al., 2019). Thus, in addition to middle-aged adult mice (10–14 months of age) (Figure 2), we also conducted RiboTag IPs from the striatum of Cre^{+} and Cre^{-} mice at postnatal ages P0, P7, P14, P21, P31, and P90 (69 mice total, n = 2–7 each for Cre^{-} and Cre^{+} mice at each age). We used a generalized linear model (GLM) within *DESeq2* (Love et al., 2014) to test whether mRNAs were significantly enriched in IP versus Input samples only in Cre^{+} mice (Figure S2A) (see STAR Methods).

We found no significant effect of genotype for any genes (Figures 3A and 3B), indicating that the single DAT$^{IRES-Cre}$ allele does not alter the striatal transcriptome. However, there was a significant effect of fraction for more than 5,000 genes (Figures 3A and 3B), demonstrating conservation of IP versus the input sample differences across genotype and age. This reflects age- and genotype-independent bias in non-specific binding of striatal polysome lysates during RiboTag IP. We found a significant effect of age for more than 7,000 genes (Figure 3B), consistent with developmental regulation of the striatal transcriptome that is conserved across fraction and genotype. Critically, we found no significant genotype:fraction interaction for any gene (Figures 3A and 3B), and thus found no statistical evidence for

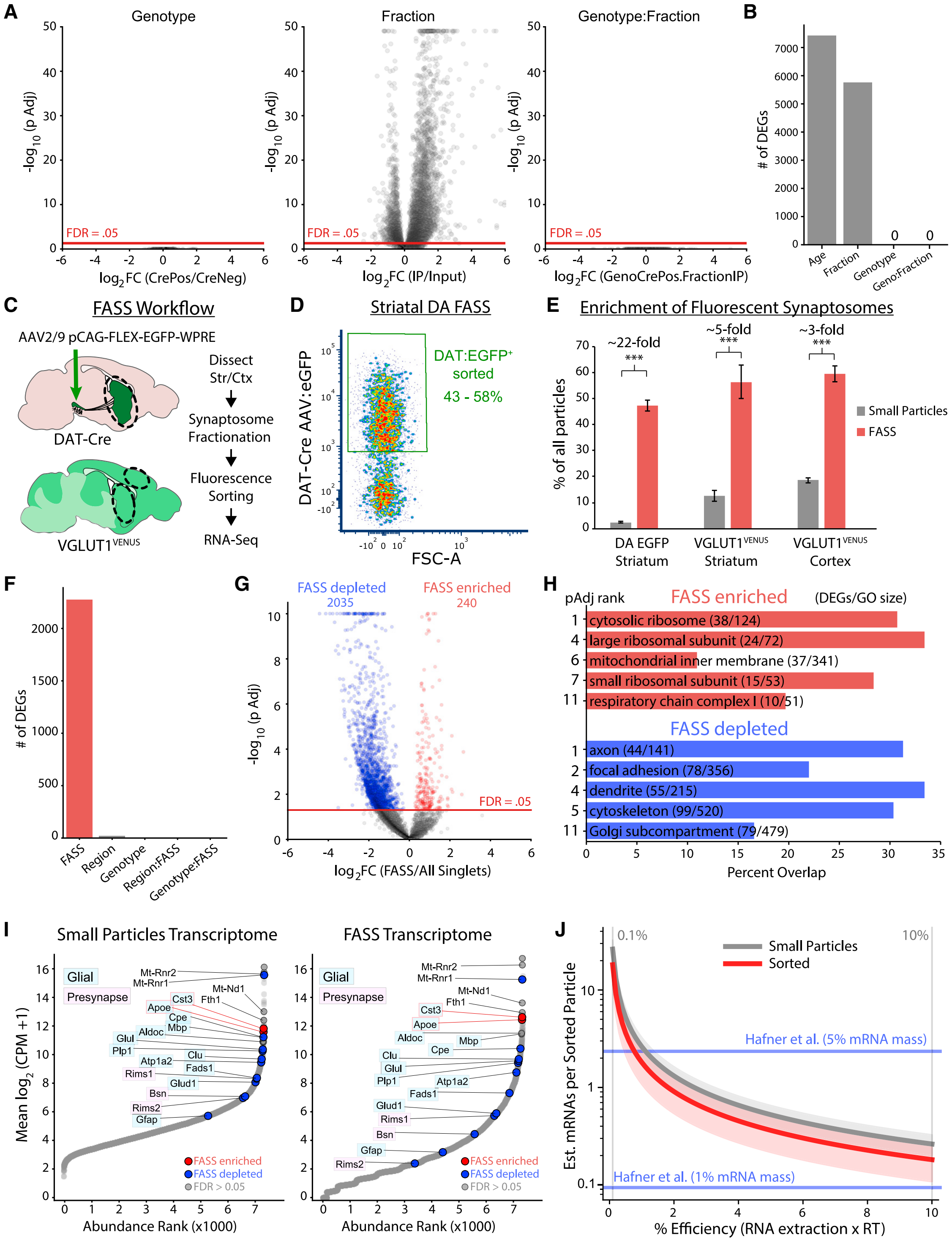

Figure 3. Striatal RiboTag IP and dopaminergic synaptosome sorting provide no evidence of translation in DA axons

Data are from P0 to adult mice in (A), (B) and adult (10–14 mo) for (C–J).

(A) *DESeq2* GLM analysis of striatal RiboTag IP and Input samples from Cre-/Cre + mice: (P0: n = 6/6, P7: n = 6/6, P14: n = 6/6, P21: n = 6/7, P31: n = 2/2, P90: n = 2/3, 10–14 mo: n = 6/4, with IP and Input samples for each mouse. *Left*: genotype effect across levels of fraction (IP and Input). *Middle*: fraction effect across levels of genotype (Cre^+ and Cre^-). *Right*: genotype:fraction interaction. log_2(GenoCrePos.FractionIP) is the difference in fraction effect between genotypes: {Cre^+ log_2FC(IP/Input) – Cre^- log_2FC(IP/Input)}. See Figure S2A schematic.

(B) Number of DEGs (FDR < 0.05) from *DESeq2* in (A).

(legend continued on next page)

age-conserved, Cre-dependent axonal RiboTag IP enrichment of any genes.

We conducted the same analyses at each age, again finding no significant effect of genotype or genotype:fraction interaction, but a significant effect of fraction for more than 1,000 genes at most ages (Figure S2B). These findings are consistent with undetectable Cre-dependent mRNA capture in all striatal RiboTag IPs, regardless of age. Furthermore, we found no significant Cre-dependent differences in *Th* or *Actb* yield (Figure S2C) or in total mRNA yield as determined by total UMIs per sample (Figure S2D). Thus, despite our RiboTag IP and RNA-seq protocol with single-cell sensitivity, we found no evidence of ribosome-bound mRNAs derived from DA axons in the striatum. The yield from Cre^{-} striatal IPs was on the order of 1–50 cells (10–500 pg total RNA via qRT-PCR and 20,000–200,000 UMIs via RNA-seq), and thus the yield of axonally derived ribosomes likely falls below this background level. We conclude that translating ribosomes in striatal DA axons are extremely sparse and not amenable to detection using striatal RiboTag IP.

Lack of dopaminergic mRNA signature in sorted striatal synaptosomes

Another approach to study the presynaptic transcriptome involves enrichment of resealed nerve terminals containing a genetically encoded fluorescent reporter (Biesemann et al., 2014; Hafner et al., 2019; Luquet et al., 2017). To directly interrogate the transcriptome of dopaminergic synaptosomes, we used our recently developed DA fluorescence activated synaptosome sorting (FASS) protocol (Paget-Blanc et al., 2021), where mDA neurons are labeled by injection of adeno-associated virus (AAV) expressing Cre-dependent eGFP into DAT-Cre mice (Figure 3C). We gated on small particles to avoid synaptosomal aggregates (Biesemann et al., 2014; Hobson and Sims, 2019) and sorted $DAT:EGFP^{+}$ particles (Figure S2E). Reanalysis revealed 43%–58% $EGFP^{+}$ particles in the sorted samples (Figure 3D), reflecting a more than 20-fold enrichment of $DAT:EGFP^{+}$ synaptosomes (Figures 3E and S2E). However, resealed axonal varicosities of mDA neurons can remain stably bound to $VGlut1^{+}$ presynaptic boutons and other synaptic elements (Paget-Blanc et al., 2021). To control for mRNA derived from co-enrichment of these other synaptic elements, we also sorted $VGlut1^{+}$ synaptosomes from the striatum and cortex of $VGlut1^{VENUS}$ mice (Figure 3C). $VGlut1^{VENUS+}$ particles were more abundant than $DAT:EGFP^{+}$ particles in striatal synaptosomes, resulting in a slightly higher sorted purity (50%–60%), but substantially lower enrichment (approximately five-fold) of $VGlut1^{VENUS+}$ synaptosomes compared with $DAT:EGFP^{+}$ synaptosomes (Figures 3E and S2E). We also sorted all small particles from each sample to control for bias owing to the small particle gating and sorting procedure.

We used the same RNA-seq protocol as above to characterize the transcriptome of FASS and small particle sorted samples. For each sample of 1.5–7.5 million particles, we obtained between 10^{4}–10^{5} UMIs (Figure S2F). For striatal samples, DAT:EGFP and $VGlut1^{VENUS}$ FASS samples yielded significantly fewer UMIs per sorted particle compared with all small particles (Figure S2G), although the yield from both sample types was only approximately 1 UMI per 50–200 sorted particles. A principal component analysis (PCA) clearly separated FASS samples from small particle controls along PC1, but DAT:EGFP FASS samples were not separated from striatal or cortical $VGlut1^{VENUS}$ FASS samples (Figures S2H and S2I). As above, we used *DESeq2* to identify differentially expressed genes (DEGs) across FASS and control samples (see STAR Methods). We found a significant effect of *FASS* for more than 2,000 genes, but no significant effect of region:FASS or genotype:FASS interactions for any gene (Figure 3F). These data demonstrate that the FASS transcriptome is distinct from all small particles, but that the $VGlut1^{VENUS}$ and DAT:EGFP sorted samples are largely indistinguishable. Given the paucity of DA axons in the cortex compared to the striatum, this result argues against any detectable contribution of DA synaptosome-specific mRNAs. Most of the 240 genes enriched in FASS samples were enriched by more than 2-fold (Figure 3G), substantially lower than the more than 20-fold enrichment of $DAT\text{-}EGFP^{+}$ particles in FASS sorted samples. No canonical dopaminergic mRNAs (e.g., *Th*, *Slc6a3/DAT*) or other DA neuron-specific mRNAs were detected in any FASS samples.

Presynaptic elements are often bound to other particles in synaptosome preparations, which can represent non-specific aggregation (Hobson and Sims, 2019) or native adherence of synaptic structures (Paget-Blanc et al., 2021). Astrocytic processes containing mRNA and ribosomes are also present in synaptosome preparations (Chicurel et al., 1993; Mazaré et al., 2020; Sakers et al., 2017) and are a likely source of mRNA in our FASS samples. We found that, despite a relative depletion by sorting, many oligodendrocyte- and astrocyte-enriched mRNAs were abundant in our striatal FASS samples, while the microglia- and astrocyte-enriched mRNAs *Cst3* and *Apoe* were enriched by sorting (Figure 3I). Many mDA axonal varicosities lack active zone scaffolding proteins and do not release DA upon stimulation (Liu et al., 2018; Pereira et al., 2016). We found no evidence for the local translation of active zone proteins in dopaminergic synaptosomes: *Rims1* and *Bsn* mRNAs had low

(C) FASS RNA-seq schematic. (D) Density plot of EGFP-sorted striatal synaptosomes from DAT-Cre mice expressing EGFP in mDA neurons.

(E) Comparison of fluorescent particles in unsorted and sorted synaptosome samples. Mean ± SEM for the $\%EGFP^{+}/VENUS^{+}$ out of all particles are plotted for: DAT:EGFP striatum (n = 6), $VGlut1^{VENUS}$ striatum (n = 3), and $VGlut1^{VENUS}$ cortex (n = 3). Two-way ANOVA main effects: region/genotype, F = 16.9 (2, 18), p = 7.3×10^{-5}; fraction, F = 427.9 (1, 18), p = 5.4×10^{-14}; and region/genotype:fraction interaction, F = 0.30 (2, 18), p = 0.74. Tukey's HSD *post hoc* test for unsorted versus sorted samples: p-adj < 0.001 for all three comparisons. ***p < 0.001.

(F) Number of DEGs (FDR < 0.05) from the *DESeq2* with indicated terms removed from the GLM: ∼FASS + region + genotype + region:FASS + genotype:FASS.

(G) Volcano plot for *DESeq2*, comparing FASS samples to all small particles (enriched/depleted genes, FDR < 0.05).

(H) GO analysis of FASS-enriched and -depleted DEGs from (G).

(I) Abundance versus rank for all small particles (*left*) and FASS samples (*right*). FASS-enriched and -depleted mRNAs are shown in red and blue, respectively.

(J) Estimated mRNAs per sorted particle as a function of FASS RNA-seq efficiency with estimates for whole forebrain $VGlut1^{VENUS}$ sorted particles from Hafner et al., 2019 (see STAR Methods).

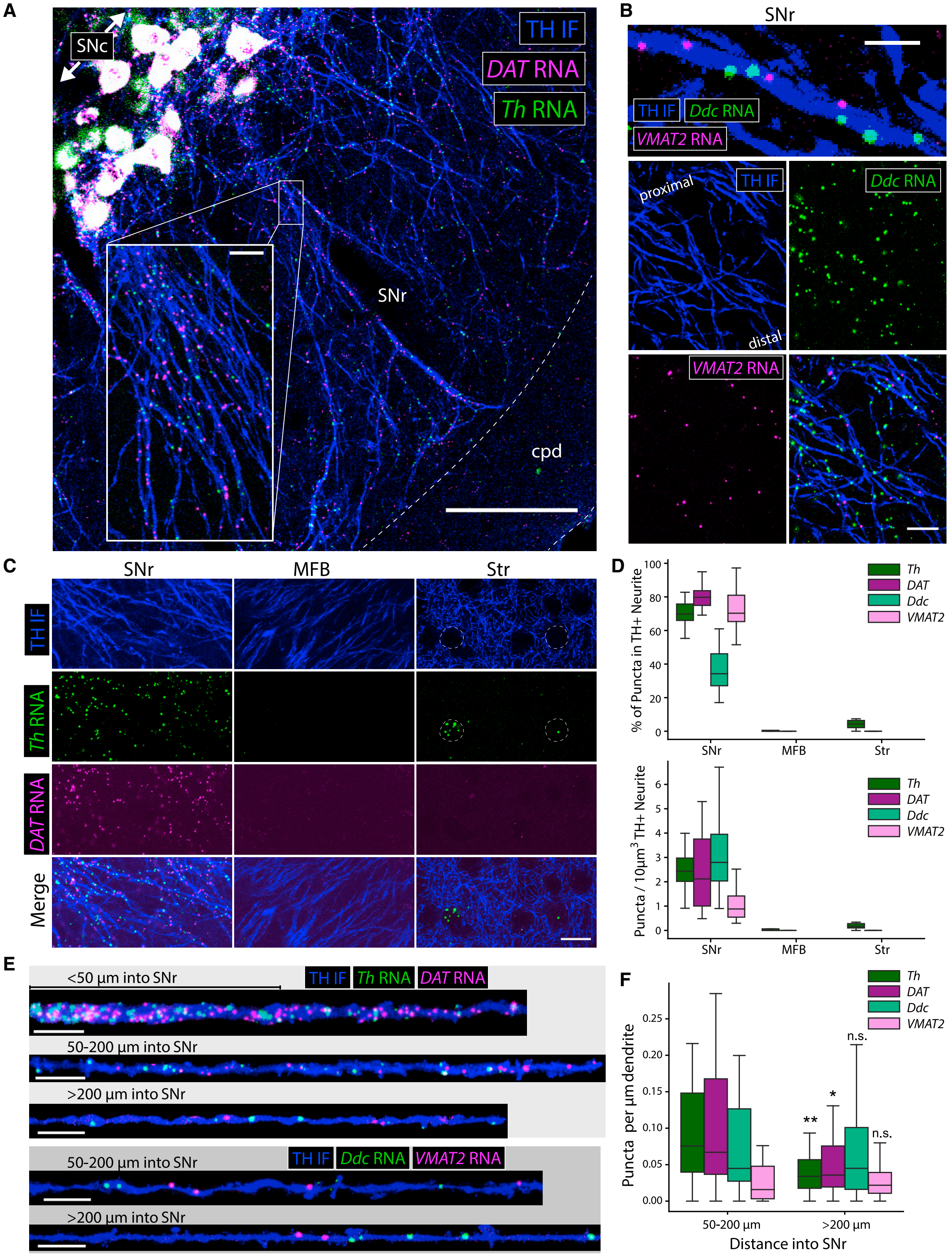

(legend on next page)

abundance in small particle samples and were further depleted by sorting (Figure 3I), suggesting that local translation does not regulate active and silent presynaptic sites in mDA axons.

A gene ontology (GO) analysis revealed that mRNAs encoding axonal, dendritic, and cytoskeletal proteins were overrepresented among the FASS-depleted mRNAs, while mRNAs encoding ribosomal proteins and nuclear-encoded mitochondrial proteins were overrepresented among FASS-enriched mRNAs (Figure 3H). Although the latter two groups of mRNAs have been observed in axons (Aschrafi et al., 2016; Briese et al., 2016; Gumy et al., 2011; Shigeoka et al., 2016, 2019; Taylor et al., 2009), they are also present in dendrites (Fusco et al., 2021; Perez et al., 2021), the synaptic neuropil (Biever et al., 2020; Cajigas et al., 2012) and perisynaptic astrocyte processes (Mazaré et al., 2020). Thus, it is unclear whether any FASS-enriched mRNAs are derived from DA synaptosomes. Since millions of synaptosomal particles yielded UMI counts similar to a single cell (Figure S2F), we estimated the mRNA content of sorted synaptosomal particles (see STAR Methods). Based on estimates of RNA extraction and reverse transcription efficiency, we estimate there are between 0.2 and 2 mRNAs per sorted synaptosomal particle (Figure 3J), consistent with the total RNA yield from VGlut1^{VENUS} FASS samples (Hafner et al., 2019) of 0.1–2.2 mRNAs per sorted synaptosomal particle (Figure 3J). In addition to the lack of a DAT:EGFP-specific signature and a major contribution of glial mRNAs to the striatal FASS transcriptome, it is possible that many striatal synaptosomes contain no mRNA. Collectively, these data provide no evidence for mRNA localization in dopaminergic synaptosomes.

In a final effort to enrich mRNAs from mDA axons, we conducted RiboTag IP on striatal synaptosomes from DAT$^{IRES-Cre}$: RiboTag mice. Similar to whole striatal IPs, we observed no significant effect for genotype or genotype:fraction interaction, while hundreds of genes were significantly affected by fraction (Figure S2J). We found no significant difference in the mRNA yield of Cre^{+} versus Cre^{-} IPs (Figure S2K), and there was no Cre-dependent bias for FASS-enriched or -depleted mRNAs (Figures S2L and S2M). Collectively, these data strongly suggest that mRNAs enriched in striatal FASS samples are not derived from ribosomes in dopaminergic varicosities.

mRNAs encoding DA transmission machinery are robustly localized to dopaminergic dendrites, but not axons

The eL22-HA staining in SNr dendrites (Figure 1) and enrichment of *Th* and *Slc6a3/DAT* mRNA in SNr RiboTag IPs (Figure 2) suggest local translation of these mRNAs in dopaminergic dendrites. We confirmed the dendritic localization of *Th* and *Slc6a3/DAT* mRNA using fluorescence *in situ* hybridization (FISH) (RNAscope). In addition to dense staining of somata in the SNc, we observed dispersed *Th* and *Slc6a3/DAT* mRNA puncta throughout the SNr (Figure S3A). No staining was observed for *DapB*, a negative control bacterial mRNA (Figure S3B). Combining FISH with TH IF revealed a striking density of *Th* and *Slc6a3/DAT* mRNA puncta within TH^{+} dendrites in the SNr (Figure 4A). We found that *Slc18a2/VMAT2* (*vesicular monoamine transporter 2*) and *Ddc* (*aromatic l-amino acid decarboxylase*) mRNAs were also localized in dopaminergic SNr dendrites (Figure 4B). Among these four dopaminergic mRNAs, only *Ddc* is expressed by other midbrain cells (Figure S3C), but *Ddc* puncta within TH^{+} dendrites were clearly distinguished from SNr cells (Figures S3D and S3E). All four mRNAs were observed within dopaminergic dendrites deep in the SNr, hundreds of microns from SNc soma (Figures 4A and S3F).

In contrast with cultured sympathetic neurons (Aschrafi et al., 2017; Gervasi et al., 2016), we found no *Th* mRNA puncta in MFB mDA axons (Figure 4C). Previous work proposed mDA axons as the source of striatal *Th* mRNA *in vivo* (Melia et al., 1994). In the striatum, we found dense clusters of *Th* mRNA puncta in soma-sized areas devoid of TH^{+} axons (Figure 4C), which likely represent *Th* mRNA^{+} interneurons that release GABA, not DA (Xenias et al., 2015) (Figure S4A). Striatal *Th* mRNA^{+} neurons occasionally expressed detectable TH protein (Figure S4B). To further establish that striatal interneurons are the source of striatal *Th* mRNA, we measured dopaminergic mRNA levels in Pitx3-KO mice, which lack dopaminergic innervation of the dorsal striatum (Lieberman et al., 2018; Nunes et al., 2003). We found a more than four-fold decrease in *Th*, *Slc6a3/DAT*, and *Slc18a2/VMAT2* mRNA in the VM, consistent with developmental cell death of SNc, but not VTA mDA neurons. However, we found no significant difference in any of these mRNAs in dorsal striatum (Figure S4C). Collectively, these data show that *Th* mRNA^{+} striatal interneurons, and not dopaminergic axons, are the source of striatal *Th* mRNA. mDA neurons may lack RNA-binding or trafficking proteins that mediate axonal *Th* mRNA localization in sympathetic neurons (Aschrafi et al., 2017; Gervasi et al., 2016); further research is needed to characterize the molecular mechanisms that control *Th* mRNA trafficking in central and peripheral catecholamine neurons.

Using stringent criteria (see STAR Methods), we found that more than 70% of all *Th*, *Slc6a3/DAT*, and *Slc18a2/VMAT2* mRNA puncta in the SNr were localized within TH^{+} dendrites (Figure 4D). Since *Ddc* is expressed in other midbrain cells, only approximately 33% of *Ddc* mRNA puncta met the co-localization criteria. In the MFB and striatum, more than 5% of *Th* and *Slc6a3/DAT* mRNA puncta were localized within dopaminergic axons (Figure 4D). We quantified mRNA density in segmented dendrites in the proximal SNr (50–200 μm from the SNc) or distal SNr

Figure 4. FISH reveals dendritic, but not axonal, localization of dopaminergic mRNAs in adult (10–14 mo) mouse brain

(A) TH IF and FISH for *Th* and *Slc6a3/DAT* mRNA in the SN, cpd: cerebral peduncle. Scale bar, 100 μm; inset scale bar, 10 μm.

(B) TH IF and FISH for *Ddc* and *Slc18a2/VMAT2* mRNA in the SNr. Scale bars, 5 μm (*upper*), 15 μm (*lower*).

(C) TH IF and FISH for *Th* and *Slc6a3/DAT* mRNA. Scale bar, 15 μm.

(D) Quantification of RNA puncta within TH^{+} neurites from each region in (C): n = 8-21 fields from 5-6 sections, 5-6 mice.

(E) TH^{+} dendrite segments and mRNAs at various distances into the SNr. Scale bars, 10 μm.

(F) Quantification of RNA puncta within TH^{+} dendrites from (E). Box and whiskers plots represent puncta per μm for each segmented dendrite in each region: (50-200μm, *Th*) n = 31-38 dendrites from 5-6 sections, 5-6 mice. *p < 0.05, **p < 0.01 for Mann-Whitney *U* test comparing >200 μm with 50-200 μm for each RNA.

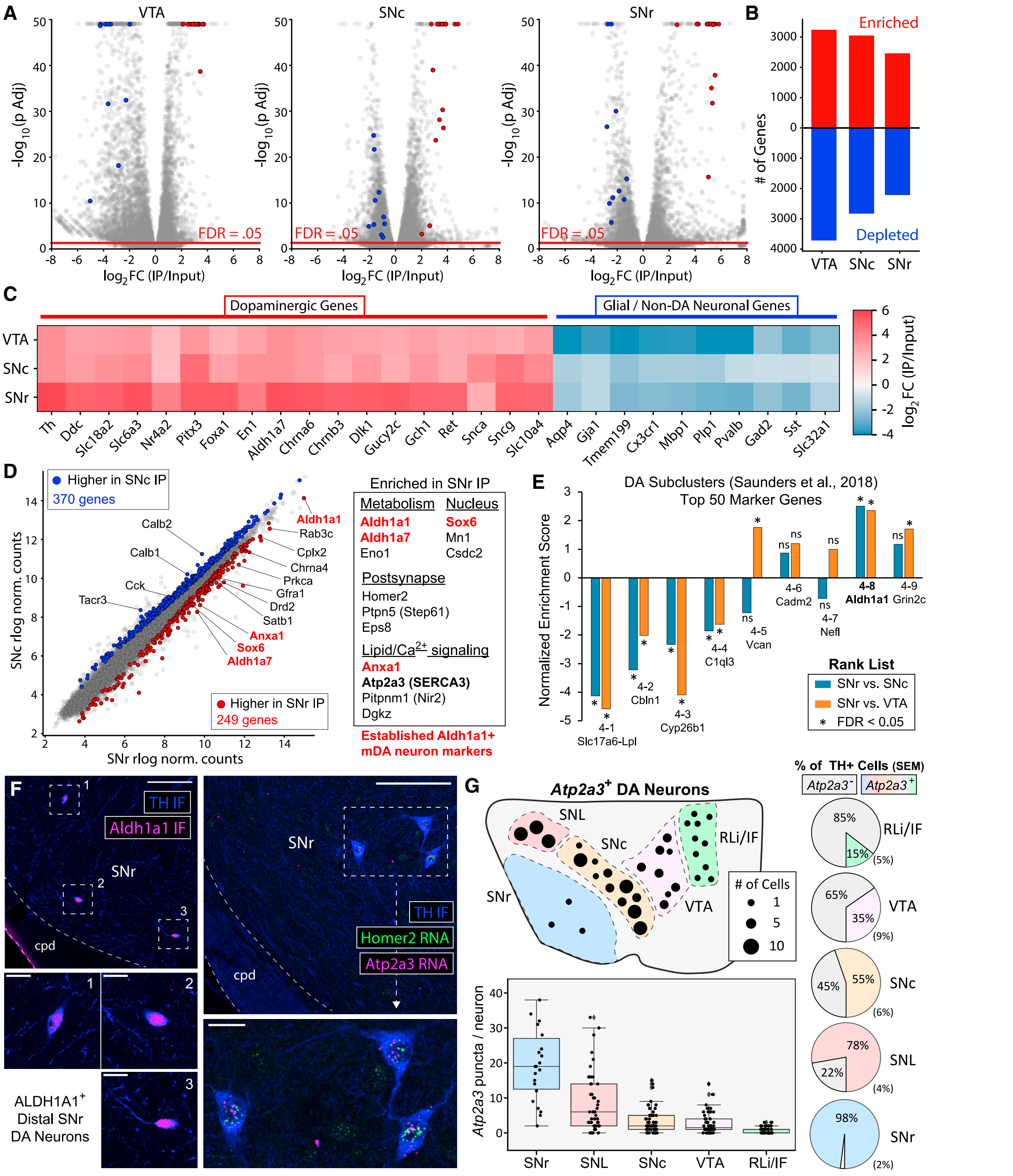

Figure 5. SNr RiboTag translatome reveals *Aldh1a1*⁺ molecular signature and *Atp2a3* (SERCA3) expression in SNr mDA neurons in adult (10–14 mo) mice

(A) RiboTag IP versus Input volcano plots for VTA, SNc, and SNr samples (n = 4 each). Red or blue points are specific to mDA neurons or to glia and non-mDA neurons, respectively, and shown in (C).

(B) Number of RiboTag IP-enriched or depleted genes (FDR < 0.05) from (A).

(C) Heatmap of average RiboTag IP enrichment for VTA, SNc, and SNr RiboTag IP (n = 4 each).

(D) Average *DESeq2* rlog normalized counts for SNr and SNc RiboTag IP samples (n = 4 each). Red and blue genes are differentially expressed between SNr and SNc IP samples. Genes in red are markers of Aldh1a1^{+}/Sox6^{+} ventral-tier SNc mDA neurons.

(legend continued on next page)

(>200 μm from the SNc) (Figure 4E). The abundance of *Th* and *Slc6a3/DAT* mRNA in proximal dendritic segments was notably greater than *Slc18a2/VMAT2* mRNA (Figure 4F). However, *Th* and *Slc6a3/DAT* mRNA abundance significantly decreased in distal dendritic segments, while *Ddc* and *Slc18a2/VMAT2* mRNA did not, so that the abundance of all four mRNAs was comparable within distal dendritic segments (Figure 4F). Thus, the mRNAs encoding DA synthesis, release, and reuptake machinery are present throughout mDA dendritic projections into the SNr.

Regional translatome profiling reveals *Aldh1a1*+/*Sox6*+ molecular profile of SNr mDA neurons

To characterize the mDA neuronal translatome in our SNr dissections (Figure 2), we conducted full-length total RNA-seq (see STAR Methods) of Input and RiboTag IP samples from the SNc, VTA, and SNr. Comparison of IP versus Input samples from Cre+ mice revealed a core enrichment signature of canonical dopaminergic genes (e.g., *Th*, *Ddc*, *Slc18a2/VMAT2*, *Slc6a3/DAT*, *Pitx3*, *En1*, *Ret*, and *Gch1)* in all three regions (Figures 5A–5C). PCA clearly separated IP versus Input samples along PC1 and Regions along PC2 (Figure S5A). The enrichment factor for genes expected to be highly enriched in mDA neurons (Brichta et al., 2015; Saunders et al., 2018) was strikingly higher in SNr IPs than in VTA and SNc IPs (Figure 5C). Together with the low yield from SNr samples (Figure 2), these results suggested that tagged ribosomes derived from a few SNr mDA neurons dominated the RNA content of our SNr RiboTag IPs. Twenty marker genes of various mDA neuronal subpopulations (Poulin et al., 2020) were sufficient for accurate clustering of RiboTag IP samples from all three regions (Figures S5B and S5C). These data demonstrate that many mDA neuronal subpopulation markers identified at the embryonic or early postnatal timepoints are still differentially translated in adult mice. The key markers of the *Aldh1a1*+/*Sox6*+ ventral-tier SNc population, which is particularly vulnerable in models of Parkinson's disease (PD) (Cai et al., 2014; Liu et al., 2014; Poulin et al., 2014), were enriched in our SNr RiboTag IPs (*Aldh1a1*, *Sox6*, *Aldh1a7*, and *Anxa1*).

We identified mRNAs differentially expressed between SNr and SNc IPs (Figure 5D), with further filtering to select only RiboTag enriched genes (see STAR Methods). We identified 249 genes with greater abundance in SNr versus SNc IPs, including the ventral-tier SNc mDA neuronal markers noted above (*Aldh1a1*, *Sox6*, *Aldh1a7*, and *Anxa1*) (Figure 5D) (Data S6). Other SNr-enriched mRNAs encoded proteins involved in lipid/calcium signaling, metabolism, and postsynaptic function (Figures 5D, S5F–S5H). Consistent with an enrichment of *Aldh1a1*+/*Sox6*+ mDA neurons in our SNr dissection, gene set enrichment analysis (GSEA) (Subramanian et al., 2005) revealed that the top 50 marker genes of the *Aldh1a1*+ cluster in Saunders et al. (2018) were significantly enriched in SNr versus SNc/VTA IPs (Figure 5E). All of the few TH+ mDA neurons within the SNr were ALDH1A1+ (Figures 5F and S6A). We used FISH to study the distribution of SNr-enriched mRNAs that were not previously described as markers of *Aldh1a1*+/*Sox6*+ mDA neurons (*Atp2a3*, *Homer2*, *Dgkz*, and *Prkca*). These mRNAs were present within mDA neuronal somata in the SNr, but we found no evidence of dendritic localization (Figure 5F, S6B–S6D). Thus, our SNr RiboTag IP predominantly reflects the translatome of SNr mDA neurons, and a molecular signature consistent with *Aldh1a1*+/*Sox6*+ mDA neurons in the ventral-tier SNc (Poulin et al., 2020; Saunders et al., 2018). These data are consistent with earlier interpretations that SNr mDA neurons are displaced SNc mDA neurons (Guyenet and Crane, 1981; van der Kooy, 1979) with electrophysiological properties similar to SNc mDA neurons (Brown et al., 2009; Richards et al., 1997).

The SNr enrichment of *Atp2a3* mRNA encoding the sarco/ER Ca^{2+}-ATPase isoform 3 (SERCA3) is of particular interest given the importance of cytosolic Ca^{2+} oscillations in mDA neurons (Zampese and Surmeier, 2020). SERCA3 is predominantly expressed in hematopoietic and endothelial cells (Bobe et al., 1994; Burk et al., 1989; Wuytack et al., 1994) and has an approximately 5-fold lower affinity for Ca^{2+} than the ubiquitous SERCA2 isoform (Lytton et al., 1992). Our RiboTag data indicate that mDA neuronal translation of *Atp2a3/SERCA3* in all VM regions, although relative abundance was greatest in the SNr (Figure 5E). We used FISH to define the anatomical distribution of *Atp2a3/SERCA3*-expressing mDA neurons. In addition to the SNr (Figure 5F), we found *Atp2a3/SERCA3*+ mDA neurons in the substantia nigra pars lateralis (SNL), SNc, VTA, and midline nuclei (rostral linear nucleus and interfascicular nucleus [RLi/IF]) (Figures S6E–S6H). While few in number, nearly all SNr mDA neurons were *Atp2a3/SERCA3*+ and expressed the highest mRNA levels per neuron (Figure 5G). mDA neurons in the SNL also express high levels of *Atp2a3/SERCA3*, while *Atp2a3/SERCA3* expression was extremely sparse in the midline nuclei (Figure 5G). Cerebellar Purkinje neurons also express SERCA3 (Baba-Aïssa et al., 1996) and exhibit pacemaker firing (Raman and Bean, 1999). Given that SERCA3 expression is altered in PD (Aguila et al., 2021) and may regulate cytosolic Ca^{2+} dynamics, future studies should investigate SERCA3 function in mDA neurons.

Midbrain synaptosome RiboTag IP reveals dendritic localization of mRNAs encoding vesicular release proteins

Another approach to identify translating mRNAs in dendrites is to combine cell type-specific ribosome IP with subcellular

(E) Pre-ranked GSEA for SNr versus SNc/VTA IP rank lists using the top 50 marker genes of mDA neuronal clusters from Saunders et al. (2018) as gene sets. *FDR < 0.05 for a rank list/gene set combination.

(F) (*Left*) TH and ALDH1A1 IF. Scale bars, 100 μm (main) and 20 μm (insets). (*Right*) FISH for *Atp2a3* (*SERCA3*) and *Homer2* in the SNr. Scale bars, 100 μm (main) and 20 μm (inset).

(G) Quantification of *Atp2a3* FISH. (*Upper left*) Anatomical representation of TH+/Atp2a3+ neurons in a single hemi-section (approximately 15 μm thick) containing the indicated VM regions (−3.2 mm posterior to Bregma). Each dot represents 1, 5, or 10 mDA neurons approximating the average of 5 hemi-sections from 4 mice: (RLi/IF) 9.8, (VTA) 31.6, (SNc) 62.8, (SNL) 28.8, and (SNr) 2.8. (*Right*) Average TH+/*Atp2a3*- or TH+/*Atp2a3*+ mDA neurons ±SEM as indicated, corresponding to the upper left. Total cell counts for each region (TH+ neurons/TH+*Atp2a3*+ neurons): RLi/IF (49/338), VTA (158/503), SNc (314/555), SNL (144/187), SNr (28/29). (*Lower left*) Boxplot of *Atp2a3* mRNA puncta per TH + neuron in the indicated regions from 5 sections, 4 mice per region: (SNr) 22 cells, (SNL) 47 cells, (SNc) 64 cells, (VTA) 58 cells, and (RLi/IF) 58 cells.

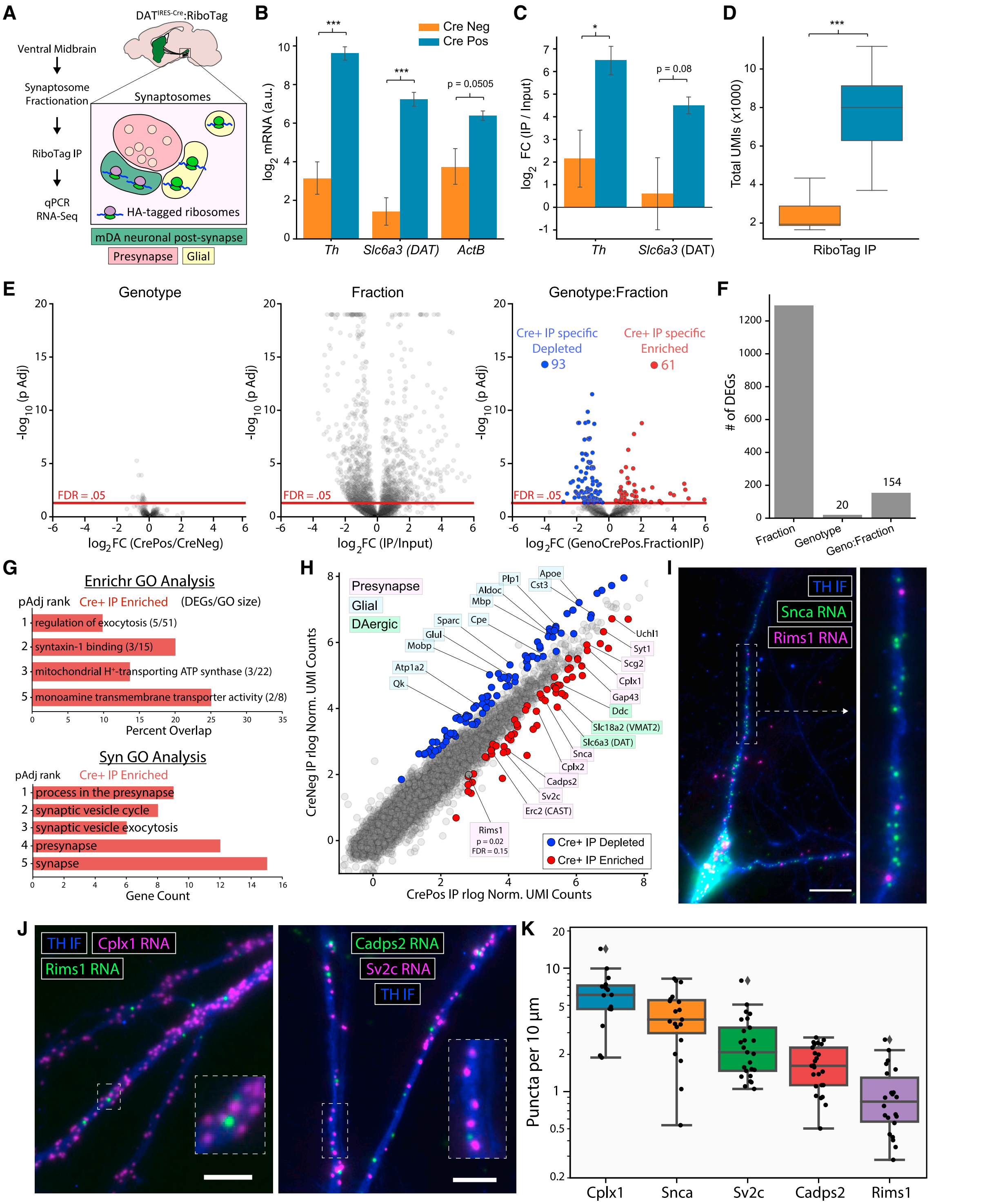

Figure 6. Dendritic localization of mRNAs encoding exocytosis and vesicular release proteins in DA neurons

Data in Figures 6A–6H are from adult mice (10–14 mo); Figures 6I–6K are from postnatal mDA neuronal cultures.

(A) Schematic for midbrain synaptosome fractionation and RiboTag IP.

(B) qRT-PCR of *Th*, *Slc6a3/DAT*, and *Actb* from RiboTag IP of VM synaptosomes from Cre^- (n = 5) and Cre^+ mice (n = 6). Log_2 mRNA abundance is in arbitrary units (40 – Cq). Two-way ANOVA main effects for genotype, F = 98.2 (1, 27), p = 1.7e-10; RNA, F = 6.10 (2, 27), p = 0.0065; and genotype:RNA interaction, F = 5.47 (2, 27), p = 0.010. Tukey's HSD *post hoc* test for Cre^- versus Cre^+ IP samples (p-adj < 0.001 for Th and DAT, p-adj = 0.0505 for ActB). ***p < 0.001.

(C) qRT-PCR for RiboTag IP/Input enrichment relative to *Actb* (mean ΔΔCq +/— SEM), n = 5 Cre^- and n = 6 Cre^+ mice. *p < 0.05, Welch's t test.

(legend continued on next page)

fractionation (Ouwenga et al., 2017, Ouwenga et al., 2018). SNc mDA neurons possess dendritic spines (Hage and Khaliq, 2015; Jang et al., 2015), and resealed dendritic elements within the midbrain synaptosome preparations exhibit DA release and reuptake (Hefti and Lichtensteiger, 1978a, 1978b; Silbergeld and Walters, 1979). We conducted RiboTag IP on synaptosomes prepared from VM tissue (Figure 6A). qRT-PCR revealed greater yield of *Th*, *Slc6a3/DAT*, and *Actb* mRNA in Cre^+ IPs compared with Cre^- controls (Figure 6B). Similarly, *Th* and *Slc6a3/DAT* enrichment was approximately 16-fold greater in Cre^+ IP versus Input comparisons compared with Cre^- controls (Figure 6C). Given the absence of local axon collaterals from mDA neurons (Omelchenko and Sesack, 2009; Tepper et al., 1987), these data demonstrate mDA neuron-specific ribosome capture from dendritic or postsynaptic elements.

We next analyzed the mDA neuronal translatome of midbrain synaptosome samples using 3′ UMI-based RNA-seq. Consistent with the increased mRNA yield measured by qRT-PCR, Cre^+ IP samples had significantly more UMIs than Cre^- controls (Figure 6D). As in the striatum, we found a significant effect of fraction for more than 1,000 genes (Figures 6E and 6F), reflecting genotype-independent non-specific binding. However, in contrast with all striatal RiboTag IP experiments, we observed a significant effect of genotype:fraction interaction for 154 genes (Figures 6E and 6F). These genes are significantly depleted (93) or enriched (61) in IP compared with Input samples only in Cre^+ mice (see Data S7). Similar to striatal synaptosomes, glial mRNAs such as *Apoe*, *Cst3*, *Cpe*, *Glul*, *Mbp*, and *Plp1* were abundant in midbrain synaptosomes; however, these glial mRNAs were uniformly depleted from Cre^+ IP samples (Figure 6H). Strikingly, GO analysis of the Cre^+ IP-enriched genes revealed a significant overrepresentation of terms such as regulation of exocytosis, process in the presynapse, and synaptic vesicle exocytosis (Figure 6G). Thus, in addition to canonical dopaminergic mRNAs, we found Cre-dependent enrichment of mRNAs encoding a wide range of proteins with presynaptic function (Figure 6H). These include mRNAs encoding proteins involved in synaptic vesicle fusion and recycling (*Erc2/CAST*, *Cplx1*, *Cplx2*, *Syt1*, *Sv2c*, and *Snca*) and dense core vesicle release (*Cadps2/CAPS2* and *Scg2*) (Figure 6H). We also observed a near-significant enrichment of *Rims1*, which encodes the active zone protein RIM1 that is involved in both axonal and somatodendritic DA release (Liu et al., 2018; Robinson et al., 2019). Many of these mRNAs have also been identified in the dendrites of both glutamatergic and GABAergic hippocampal neurons (Perez et al., 2021) (Figure S7A).

We validated the dendritic localization of several mRNAs encoding release proteins in mDA neuron cultures (Rayport et al., 1992). We first confirmed that cultured mDA neurons recapitulate the dendritic localization of *Th*, *Ddc*, *Slc6a3/DAT*, and *Slc18a2/VMAT2* mRNAs observed in the VM (Figures S7B–S7E). Although α-synuclein is abundant in presynaptic varicosities, we found dense localization of *Snca* mRNA in dendrites (Figure 6I). Similarly, we found a striking density of *Cplx1* (Complexin 1) mRNA in dendrites, along with scattered *Rims1* mRNA (Figure 6J). The Ca^{2+}-dependent activator protein of secretion 2 (CAPS2) is involved in catecholamine loading into dense core vesicles in neuroendocrine cells (Brunk et al., 2009; Ratai et al., 2019) and is particularly enriched in mDA neurons (Sadakata et al., 2006). We found *Cadps2/CAPS2* mRNA within dopaminergic dendrites, along with *Sv2c* mRNA (Figure 6J). Synaptic vesicle glycoprotein 2C (SV2C) is involved in axonal DA release (Dunn et al., 2017) and may also play a role in somatodendritic DA release. Consistent with the midbrain synaptosome IP (Figure 6H), quantification of mRNA puncta revealed that *Cplx1* and *Snca* were most abundant in dendrites, followed by *Sv2c* and *Cadps2*, and *Rims1* (Figure 6K). Collectively, these data suggest that the local translation of vesicular release proteins may regulate dendritic DA release in mDA neurons.

DISCUSSION

Predominance of somatic translation in mDA neurons

We used multiple approaches to characterize the subcellular distribution of tagged ribosomes in mDA neurons, each of which identified the soma as the major site of protein synthesis. The absence of axonal mRNA localization by FISH (Figure 4) and lack of mRNA capture by striatal RiboTag IP (Figure 3) supports the absence of translating ribosomes in mDA axons. The paucity of eL22-HA in mDA axons is surprising, given their massive axonal arborization. Strikingly, while the striatal axons of SNc mDA neurons likely comprise more than 90%–95% of their cellular volume (e.g., Matsuda et al., 2009) and approximately 90% of their cellular protein (Hobson et al., 2021), we found only approximately 1% of eL22-HA in the striatum (Figure 2D). If all of this eL22-HA were present in functional ribosomes, the axonal ribosome/protein ratio would be 10^3–10^4 lower than in mDA neuronal perikarya. Our results suggest that mDA striatal axons are supplied by a combination of slow and fast axonal transport (Maday et al., 2014; Roy, 2014) of somatically synthesized proteins. Indeed, the massive bioenergetic burden placed on axonal transport systems in mDA neurons may contribute to their demise in PD (Chu et al., 2012; Sulzer, 2007).

(D) UMIs per sample for VM synaptosome RiboTag IPs from Cre^- (n = 6) and Cre^+ mice (n = 8). ***p < 0.001, Welch's t test.
(E) Volcano plots from *DESeq2* with GLM: ∼*genotype* + *fraction* + *genotype:fraction*. (*Left*) Genotype effect. (*Middle*) fraction effect. (*Right*) Genotype:fraction interaction. log_2(GenoCrePos.FractionIP) is the difference in the fraction effect between genotypes: {Cre^+ log_2FC(IP/Input) – Cre^- log_2FC(IP/Input)}.
(F) Number of DEGs (FDR < 0.05) from (E).
(G) GO analysis (upper: *Enrichr*; lower, *SynGO*) of Cre^+ IP enriched genes from (E).
(H) Average *DESeq2* rlog normalized UMI counts for Cre^+ (n = 8) and Cre^- (n = 6) VM synaptosome IPs. Red/blue genes indicate Cre^+ IP enriched/depleted genes (FDR < 0.05). Genes with dopaminergic (*green*), glial (*blue*), and presynaptic function (pink) are labeled.
(I) TH IF and FISH (RNAscope) for *Snca* and *Rims1* mRNA in cultured mDA neurons. Dashed white lines indicate the inset (right). Scale bar, 20 μm.
(J) TH IF and FISH for *Cplx1* and *Rims1* (*left*) or *Cadps2* and *Sv2c* mRNA (*right*) in the dendrites of cultured mDA neurons. Dashed white lines indicate inset (right). Scale bars, 10 μm. (K) Quantification of RNA puncta from (I–J) per 10 μm of dendrite. Data are from 2-3 independent cultures, with n dendrites quantified for each mRNA: *Cplx1* (n = 16), *Snca* (n = 19), *Sv2c* (n = 25), and *Cadps2* (n = 25), *Rims1* (n = 35).

Dopaminergic mRNA localization and translation in dendrites

In addition to their massive axonal arbors, SNc mDA neurons must supply SNr dendrites with machinery for DA synthesis, release, and reuptake. Here, we show that *Th*, *Ddc*, *Slc6a3/DAT*, and *Slc18a2/VMAT2* mRNAs are localized throughout mDA neuronal dendrites in the SNr (Figure 4) and are bound to dopaminergic ribosomes in midbrain synaptosomes (Figure 6). In conjunction with vesicular sorting mechanisms (Li et al., 2005), dendritic translation could rapidly modify the local abundance of DA transmission machinery. DAT is often localized on vesicular and tubular membrane elements within dendrites (Hersch et al., 1997; Nirenberg et al., 1996a). VMAT2 is also found on similar structures, termed tubulovesicles, that seem to consist of smooth ER and may represent the site of dendritic DA storage and release (Cheramy et al., 1981; Nirenberg et al., 1996b). The local synthesis of DAT and VMAT2 would require the presence of dendritic ER and a local secretory pathway. Although mDA neuronal dendrites are devoid of obvious Golgi apparatus in ultrastructural studies (Nirenberg et al., 1996b), local processing could occur in Golgi outposts (Hanus and Ehlers, 2016) or recycling endosomes (Bowen et al., 2017). The tubulovesicles could also be related to ribosome-associated vesicles, a novel ER subcompartment found in secretory cells and neuronal dendrites (Carter et al., 2020). Thus, the translation of DAT and VMAT2 within local secretory pathways may be linked to the biogenesis of DA storage and release structures within mDA dendrites.

Beyond the core dopaminergic machinery, how do mDA neurons manage simultaneous axonal and dendritic localization of vesicular release proteins? For proteins involved in both axonal and somatodendritic DA release, such as RIM1 (Liu et al., 2018; Robinson et al., 2019), our data show that protein trafficking supplies the vast majority of protein to striatal mDA axons. In contrast, the local translation of RIM1 and other release proteins such as complexins may be important for establishing exocytic release sites in dopaminergic dendrites (Figure 6). Postsynaptic complexins are known to regulate AMPA receptor exocytic events during long-term potentiation (Ahmad et al., 2012) and could be involved in dendritic DA release.

The molecular characteristics of the organelles and fusion mechanisms that mediate somatodendritic DA release remain unclear (Rice and Patel, 2015). When expressed in hippocampal neurons, VMAT2 colocalizes with brain-derived neurotrophic factor on vesicles that undergo regulated exocytosis in dendrites (Li et al., 2005). Given that CAPS2 regulates the release of neurotrophin-containing vesicles in cerebellar granule cells (Sadakata et al., 2004) and co-localizes with SNAP-25 in mDA neuronal soma (Sadakata et al., 2006), the dendritic translation of *Cadps2/CAPS2* mRNA (Figure 6) raises the possibility of CAPS2 involvement in dendritic DA release. More broadly, it is possible that the trafficking of synaptic vesicle release proteins in mDA neurons has been optimized to shuttle them into striatal axons, and that such a polarization is incompatible with simultaneous trafficking into dendrites. Local translation in dopaminergic dendrites may provide an alternative mechanism of localization for these proteins, enabling the dynamic regulation of proteins at the precise intracellular sites of dendritic exocytosis.

Limitations of the study

While our findings are supported by multiple, independent approaches, there are limitations. We cannot definitively exclude the possibility that extremely low levels of axonal ribosomes are present below our limit of detection, since the visualization of eL22-HA in retinal axons required immunoelectron microscopy (Shigeoka et al., 2016). Tagged ribosomal proteins did not prevent the axonal localization of ribosomes in other neurons (Ostroff et al., 2019; Shigeoka et al., 2016), but we also cannot rule out that the HA-tag in eL22 somehow interferes with axonal localization in mDA neurons. However, we were able to reliably detect low levels of eL22-HA on translating ribosomes in mDA neuronal dendrites. We found that the presence of only a few mDA neuronal soma in the SNr dominated dendritic ribosomes in RiboTag IPs from SNr dissections (Figure 5); future studies of dendritic DA release in the SNr should be designed to avoid SNr mDA neurons. In our synaptosome RiboTag IP studies of the dendritic translatome, we confirmed the dendritic localization of key candidate mRNAs using FISH (Figure 6). Nonetheless, it is possible that some mDA neuronal ribosomes in the synaptosome fraction are derived from non-synaptic cellular elements. Future work on subcellular mRNA localization may leverage super-resolution imaging (Alon et al., 2021; Eng et al., 2019; Wang et al., 2020).

STAR★METHODS

Detailed methods are provided in the online version of this paper and include the following:

- KEY RESOURCES TABLE
- RESOURCE AVAILABILITY
  - Lead contact
  - Materials availability
  - Data and code availability
- EXPERIMENTAL MODEL AND SUBJECT DETAILS
  - Animals
- METHOD DETAILS
  - Viral injections
  - Neuronal cultures
  - Immunohistochemistry
  - Tissue dissection for RiboTag IP
  - Synaptosome preparation for RiboTag IP
  - Fluorescence-activated synaptosome sorting
  - FISH
  - RiboTag ribosome IP
  - Quantitative RT-PCR
  - Western blotting
  - Image acquisition
  - eL22-HA image analysis
  - FISH image analysis
  - Full-length total RNA-seq
  - Low input RNA-seq with 96-well plate, pooled library construction
  - Synaptosome mRNA content estimation
  - RNA-seq differential expression analysis
  - GSEA and GO analysis
- QUANTIFICATION AND STATISTICAL ANALYSIS

CellPress
OPEN ACCESS

SUPPLEMENTAL INFORMATION

Supplemental information can be found online at https://doi.org/10.1016/j.celrep.2021.110208.

ACKNOWLEDGMENTS

This work was conducted in collaboration with the JP Sulzberger Columbia Genome Center. This research was funded in part by Aligning Science Across Parkinson's [ASAP-000375] (DS and PAS) through the Michael J. Fox Foundation for Parkinson's Research. For the purpose of open access, the author has applied a CC-BY public copyright license to all Author Accepted Manuscripts arising from this submission. This work was supported by the JPB Foundation (DS). This work was supported by NIH grants F30 DA047775-03 (BDH), R01 NS095435 (DS), R01 DA07418 (DS), and R01 MH122470 (DS). This study benefited from the Agence Nationale de la Recherche consortium fundings (IDEX Bordeaux ANR-10-IDEX-03-02; Labex BRAIN ANR-10-LABX-43 BRAIN; France Bio Imaging ANR-10-INBS-04). EH received salary funding for MFA from Fondation pour la Recherche Médicale (ING20150532192). We thank Vanessa Morales for assistance with animal colony management and Ellen Kanter for assistance with dopamine neuron cultures. Our work benefitted from the core facilities at Bordeaux university: Bordeaux Imaging Center (CNRS UMS 3420, INSERM US4); Biochemistry and Biophysics of Proteins; Flow cytometry UB'FACSility (CNRS UMS 3427, INSERM US5); animal care & breeding; genotyping.

AUTHOR CONTRIBUTIONS

BDH conceived the overall project with input from PAS and DS. BDH executed all dissection, IP, Western blotting, qRT-PCR, and RNA-seq experiments. MFA and BDH performed FASS experiments with input and supervision from EH. BDH performed histology and IF experiments with assistance from OJL and EVM. BDH performed FISH experiments with assistance from LK. BDH conducted all image analysis. BDH conducted RNA-seq analysis with input from PAS. PAS, DS, and EH supervised the research. BDH wrote the manuscript with input from PAS, DS, and EH. All authors edited, read, and approved the final manuscript.

DECLARATION OF INTERESTS

The authors declare no competing interests.

Received: July 30, 2021
Revised: October 25, 2021
Accepted: December 13, 2021
Published: January 11, 2022

REFERENCES

Adams, J.C. (1992). Biotin amplification of biotin and horseradish peroxidase signals in histochemical stains. J. Histochem. Cytochem. *40*, 1457–1463.

Aguila, J., Cheng, S., Kee, N., Cao, M., Wang, M., Deng, Q., and Hedlund, E. (2021). Spatial RNA sequencing identifies robust markers of vulnerable and resistant human midbrain dopamine neurons and their expression in Parkinson's disease. Front. Mol. Neurosci. *14*, 699562.

Ahmad, M., Polepalli, J.S., Goswami, D., Yang, X., Kaeser-Woo, Y.J., Südhof, T.C., and Malenka, R.C. (2012). Postsynaptic complexin controls AMPA receptor exocytosis during LTP. Neuron *73*, 260–267.

Aschrafi, A., Gioio, A.E., Dong, L., and Kaplan, B.B. (2017). Disruption of the axonal trafficking of tyrosine hydroxylase mRNA impairs catecholamine biosynthesis in the axons of sympathetic neurons. eNeuro. *4*, ENEURO.0385–16.2017.

Aschrafi, A., Kar, A.N., Gale, J., Elkahloun, A.G., Vargas, J.-N., Sales, N., Wilson, G., Tompkins, M., Gioio, A.E., and Kaplan, B.B. (2016). A heterogeneous population of nuclear-encoded mitochondrial mRNAs is present in the axons of primary sympathetic neurons. Mitochondrion *30*, 18–23.

Alon, S., Goodwin, D.R., Sinha, A., Wassie, A.T., Chen, F., Daugharthy, E.R., Bando, Y., Kajita, A., Xue, A.G., Marrett, K., et al. (2021). Expansion sequencing: spatially precise in situ transcriptomics in intact biological systems. Science *371*, eaax2656.

Ashburner, M., Ball, C.A., Blake, J.A., Botstein, D., Butler, H., Cherry, J.M., Davis, A.P., Dolinski, K., Dwight, S.S., Eppig, J.T., et al. (2000). Gene Ontology: tool for the unification of biology. Nat. Genet. *25*, 25–29.

Baba-Aïssa, F., Raeymaekers, L., Wuytack, F., Callewaert, G., Dode, L., Missiaen, L., and Casteels, R. (1996). Purkinje neurons express the SERCA3 isoform of the organellar type Ca2+-transport ATPase. Mol. Brain Res. *41*, 169–174.

Bäckman, C.M., Malik, N., Zhang, Y., Shan, L., Grinberg, A., Hoffer, B.J., Westphal, H., and Tomac, A.C. (2006). Characterization of a mouse strain expressing Cre recombinase from the 3' untranslated region of the dopamine transporter locus. Genesis *44*, 383–390.

Banerjee, A., Lee, J., Nemcova, P., Liu, C., and Kaeser, P.S. (2020). Synaptotagmin-1 is the Ca2+ sensor for fast striatal dopamine release. Elife *9*, e58359.

Biesemann, C., Grønborg, M., Luquet, E., Wichert, S.P., Bernard, V., Bungers, S.R., Cooper, B., Varoqueaux, F., Li, L., Byrne, J.A., et al. (2014). Proteomic screening of glutamatergic mouse brain synaptosomes isolated by fluorescence activated sorting. EMBO J. *33*, 157–170.

Biever, A., Glock, C., Tushev, G., Ciirdaeva, E., Dalmay, T., Langer, J.D., and Schuman, E.M. (2020). Monosomes actively translate synaptic mRNAs in neuronal processes. Science. *367*, eaay4991.

Björklund, A., and Dunnett, S.B. (2007). Dopamine neuron systems in the brain: an update. Trends Neurosci. *30*, 194–202.

Bobe, R., Bredoux, R., Wuytack, F., Quarck, R., Kovàcs, T., Papp, B., Corvazier, E., Magnier, C., and Enouf, J. (1994). The rat platelet 97-kDa Ca2+ATPase isoform is the sarcoendoplasmic reticulum Ca2+ATPase 3 protein. J. Biol. Chem. *269*, 1417–1424.

Bobrow, M.N., Litt, G.J., Shaughnessy, K.J., Mayer, P.C., and Conlon, J. (1992). The use of catalyzed reporter deposition as a means of signal amplification in a variety of formats. J. Immunol. Methods *150*, 145–149.

Bowen, A.B., Bourke, A.M., Hiester, B.G., Hanus, C., and Kennedy, M.J. (2017). Golgi-independent secretory trafficking through recycling endosomes in neuronal dendrites and spines. ELife *6*, e27362.

Bradshaw, K.D., Emptage, N.J., and Bliss, T.V.P. (2003). A role for dendritic protein synthesis in hippocampal late LTP. Eur. J. Neurosci. *18*, 3150–3152.

Brichta, L., Shin, W., Jackson-Lewis, V., Blesa, J., Yap, E.-L., Walker, Z., Zhang, J., Roussarie, J.-P., Alvarez, M.J., Califano, A., et al. (2015). Identification of neurodegenerative factors using translatome-regulatory network analysis. Nat. Neurosci. *18*, 1325–1333.

Briese, M., Saal, L., Appenzeller, S., Moradi, M., Baluapuri, A., and Sendtner, M. (2016). Whole transcriptome profiling reveals the RNA content of motor axons. Nucleic Acids Res. *44*, e33.

Brown, M.T.C., Henny, P., Bolam, J.P., and Magill, P.J. (2009). Activity of neurochemically heterogeneous dopaminergic neurons in the substantia nigra during spontaneous and driven changes in brain state. J. Neurosci. *29*, 2915–2925.

Brunk, I., Blex, C., Speidel, D., Brose, N., and Ahnert-Hilger, G. (2009). Ca2+-dependent activator proteins of secretion promote vesicular monoamine uptake. J. Biol. Chem. *284*, 1050–1056.

Burk, S.E., Lytton, J., MacLennan, D.H., and Shull, G.E. (1989). cDNA cloning, functional expression, and mRNA tissue distribution of a third organellar Ca2+ pump*. J. Biol. Chem. *264*, 18561–18568.

Cai, H., Liu, G., Sun, L., and Ding, J. (2014). Aldehyde Dehydrogenase 1 making molecular inroads into the differential vulnerability of nigrostriatal dopaminergic neuron subtypes in Parkinson's disease. Transl. Neurodegenerat. *3*, 27.

Cajigas, I.J., Tushev, G., Will, T.J., tom Dieck, S., Fuerst, N., and Schuman, E.M. (2012). The local transcriptome in the synaptic neuropil revealed by deep sequencing and high-resolution imaging. Neuron *74*, 453–466.

Carter, S.D., Hampton, C.M., Langlois, R., Melero, R., Farino, Z.J., Calderon, M.J., Li, W., Wallace, C.T., Tran, N.H., Grassucci, R.A., et al. (2020).

Ribosome-associated vesicles: a dynamic subcompartment of the endoplasmic reticulum in secretory cells. Sci. Adv. *6*, eaay9572.

Chen, B.T., and Rice, M.E. (2001). Novel Ca2+ dependence and time course of somatodendritic dopamine release: substantia nigra versus striatum. J. Neurosci. *21*, 7841–7847.

Cheramy, A., Leviel, V., and Glowinski, J. (1981). Dendritic release of dopamine in the substantia nigra. Nature *289*, 537–542.

Chicurel, M.E., Terrian, D.M., and Potter, H. (1993). mRNA at the synapse: analysis of a synaptosomal preparation enriched in hippocampal dendritic spines. J. Neurosci. *13*, 4054–4063.

Chu, Y., Morfini, G.A., Langhamer, L.B., He, Y., Brady, S.T., and Kordower, J.H. (2012). Alterations in axonal transport motor proteins in sporadic and experimental Parkinson's disease. Brain *135*, 2058–2073.

Costa, R.O., Martins, H., Martins, L.F., Cwetsch, A.W., Mele, M., Pedro, J.R., Tomé, D., Jeon, N.L., Cancedda, L., Jaffrey, S.R., et al. (2019). Synaptogenesis stimulates a proteasome-mediated ribosome reduction in axons. Cell Rep. *28*, 864–876.e6.

Cracco, J.B., Serrano, P., Moskowitz, S.I., Bergold, P.J., and Sacktor, T.C. (2005). Protein synthesis-dependent LTP in isolated dendrites of CA1 pyramidal cells. Hippocampus *15*, 551–556.

Crispino, M., Chun, J.T., Cefaliello, C., Perrone Capano, C., and Giuditta, A. (2014). Local gene expression in nerve endings. Dev. Neurobiol. *74*, 279–291.

Dobin, A., Davis, C.A., Schlesinger, F., Drenkow, J., Zaleski, C., Jha, S., Batut, P., Chaisson, M., and Gingeras, T.R. (2013). STAR: ultrafast universal RNA-seq aligner. Bioinformatics *29*, 15–21.

Dunn, A.R., Stout, K.A., Ozawa, M., Lohr, K.M., Hoffman, C.A., Bernstein, A.I., Li, Y., Wang, M., Sgobio, C., Sastry, N., et al. (2017). Synaptic vesicle glycoprotein 2C (SV2C) modulates dopamine release and is disrupted in Parkinson disease. Proc. Natl. Acad. Sci. U S A. *114*, E2253–E2262.

Eng, C.-H.L., Lawson, M., Zhu, Q., Dries, R., Koulena, N., Takei, Y., Yun, J., Cronin, C., Karp, C., Yuan, G.-C., et al. (2019). Transcriptome-scale super-resolved imaging in tissues by RNA seqFISH+. Nature *568*, 235–239.

Fusco, C.M., Desch, K., Dörrbaum, A.R., Wang, M., Staab, A., Chan, I.C.W., Vail, E., Villeri, V., Langer, J.D., and Schuman, E.M. (2021). Neuronal ribosomes dynamically exchange ribosomal proteins in a context-dependent manner. BioRxiv, 2021.03.25.437026.

Geffen, L.B., Jessell, T.M., Cuello, A.C., and Iversen, L.L. (1976). Release of dopamine from dendrites in rat substantia nigra. Nature *260*, 258–260.

Gene Ontology Consortium (2021). The Gene Ontology resource: enriching a GOld mine. Nucleic Acids Res. *49*, D325–D334.

Gervasi, N.M., Scott, S.S., Aschrafi, A., Gale, J., Vohra, S.N., MacGibeny, M.A., Kar, A.N., Gioio, A.E., and Kaplan, B.B. (2016). The local expression and trafficking of tyrosine hydroxylase mRNA in the axons of sympathetic neurons. RNA *22*, 883–895.

Gumy, L.F., Yeo, G.S.H., Tung, Y.-C.L., Zivraj, K.H., Willis, D., Coppola, G., Lam, B.Y.H., Twiss, J.L., Holt, C.E., and Fawcett, J.W. (2011). Transcriptome analysis of embryonic and adult sensory axons reveals changes in mRNA repertoire localization. RNA *17*, 85–98.

Guyenet, P.G., and Crane, J.K. (1981). Non-dopaminergic nigrostriatal pathway. Brain Res. *213*, 291–305.

Hafner, A.-S., Donlin-Asp, P.G., Leitch, B., Herzog, E., and Schuman, E.M. (2019). Local protein synthesis is a ubiquitous feature of neuronal pre- and postsynaptic compartments. Science *364*, eaau3644.

Hage, T.A., and Khaliq, Z.M. (2015). Tonic firing rate controls dendritic Ca2+ signaling and synaptic gain in substantia nigra dopamine neurons. J. Neurosci. *35*, 5823–5836.

Hanus, C., and Ehlers, M.D. (2016). Specialization of biosynthetic membrane trafficking for neuronal form and function. Curr. Opin. Neurobiol. *39*, 8–16.

Hefti, F., and Lichtensteiger, W. (1978a). Dendritic dopamine: studies on the release of endogenous dopamine from subcellular particles derived from dendrites of rat nigro-striatal neurons. Neurosci. Lett. *10*, 65–70.

Hefti, F., and Lichtensteiger, W. (1978b). Subcellular distribution of dopamine in substantia Nigra of the rat brain: effects of γ-butyrolactone and destruction of noradrenergic afferents suggest formation of particles from dendrites1. J. Neurochem. *30*, 1217–1230.

Hersch, S.M., Yi, H., Heilman, C.J., Edwards, R.H., and Levey, A.I. (1997). Subcellular localization and molecular topology of the dopamine transporter in the striatum and substantia nigra. J. Comp. Neurol. *388*, 211–227.

Herzog, E., Nadrigny, F., Silm, K., Biesemann, C., Helling, I., Bersot, T., Steffens, H., Schwartzmann, R., Nägerl, U.V., El Mestikawy, S., et al. (2011). In vivo imaging of intersynaptic vesicle exchange using VGLUT1 Venus knock-in mice. J. Neurosci. *31*, 15544–15559.

Hobson, B.D., and Sims, P.A. (2019). Critical analysis of particle detection artifacts in synaptosome flow cytometry. eNeuro *6*, ENEURO.0009–19.2019.

Hobson, B.D., Choi, S.J., Soni, R.K., Sulzer, D., and Sims, P.A. (2021). Subcellular proteomics of dopamine neurons in the mouse brain reveals axonal enrichment of proteins encoded by Parkinson's disease-linked genes. BioRxiv, 2021.06.01.446584.

Huber, K.M., Kayser, M.S., and Bear, M.F. (2000). Role for rapid dendritic protein synthesis in hippocampal mGluR-dependent long-term depression. Science *288*, 1254–1257.

Jang, M., Um, K.B., Jang, J., Kim, H.J., Cho, H., Chung, S., and Park, M.K. (2015). Coexistence of glutamatergic spine synapses and shaft synapses in substantia nigra dopamine neurons. Sci. Rep. *5*, 14773.

Jung, H., Yoon, B.C., and Holt, C.E. (2012). Axonal mRNA localization and local protein synthesis in nervous system assembly, maintenance and repair. Nat. Rev. Neurosci. *13*, 308–324.

Juraska, J.M., Wilson, C.J., and Groves, P.M. (1977). The substantia nigra of the rat: a Golgi study. J. Comp. Neurol. *172*, 585–600.

Kang, H., and Schuman, E.M. (1996). A requirement for local protein synthesis in neurotrophin-induced hippocampal synaptic plasticity. Science *273*, 1402–1406.

Koopmans, F., Nierop, P.van, Andres-Alonso, M., Byrnes, A., Cijsouw, T., Coba, M.P., Cornelisse, L.N., Farrell, R.J., Goldschmidt, H.L., Howrigan, D.P., et al. (2019). SynGO: an evidence-based, expert-curated knowledge base for the synapse. Neuron *103*, 217–234.e4.

van der Kooy, D. (1979). The organization of the thalamic, nigral and raphe cells projecting to the medial vs lateral caudate-putamen in rat. A fluorescent retrograde double labeling study. Brain Res. *169*, 381–387.

Korf, J., Zieleman, M., and Westerink, B.H.C. (1976). Dopamine release in substantia nigra? Nature *260*, 257–258.

Lammel, S., Steinberg, E.E., Földy, C., Wall, N.R., Beier, K., Luo, L., and Malenka, R.C. (2015). Diversity of transgenic mouse models for selective targeting of midbrain dopamine neurons. Neuron *85*, 429–438.

Langmead, B., and Salzberg, S.L. (2012). Fast gapped-read alignment with Bowtie 2. Nat. Methods *9*, 357–359.

Li, H., Waites, C.L., Staal, R.G., Dobryy, Y., Park, J., Sulzer, D.L., and Edwards, R.H. (2005). Sorting of vesicular monoamine transporter 2 to the regulated secretory pathway confers the somatodendritic exocytosis of monoamines. Neuron *48*, 619–633.

Liao, Y., Smyth, G.K., and Shi, W. (2014). featureCounts: an efficient general purpose program for assigning sequence reads to genomic features. Bioinformatics *30*, 923–930.

Lieberman, O.J., McGuirt, A.F., Mosharov, E.V., Pigulevskiy, I., Hobson, B.D., Choi, S., Frier, M.D., Santini, E., Borgkvist, A., and Sulzer, D. (2018). Dopamine triggers the maturation of striatal spiny projection neuron excitability during a critical period. Neuron *99*, 540–554.e4.

Liu, C., Kershberg, L., Wang, J., Schneeberger, S., and Kaeser, P.S. (2018). Dopamine secretion is mediated by sparse active zone-like release sites. Cell *172*, 706–718.e15.

Liu, G., Yu, J., Ding, J., Xie, C., Sun, L., Rudenko, I., Zheng, W., Sastry, N., Luo, J., Rudow, G., et al. (2014). Aldehyde dehydrogenase 1 defines and protects a nigrostriatal dopaminergic neuron subpopulation. J. Clin. Invest. *124*, 3032–3046.

Love, M.I., Huber, W., and Anders, S. (2014). Moderated estimation of fold change and dispersion for RNA-seq data with DESeq2. Genome Biol. *15*, 550.

Luquet, E., Biesemann, C., Munier, A., and Herzog, E. (2017). Purification of synaptosome populations using fluorescence-activated synaptosome sorting. Methods Mol. Biol. *1538*, 121–134.

Lytton, J., Westlin, M., Burk, S.E., Shull, G.E., and MacLennan, D.H. (1992). Functional comparisons between isoforms of the sarcoplasmic or endoplasmic reticulum family of calcium pumps. J. Biol. Chem. *267*, 14483–14489.

Maday, S., Twelvetrees, A.E., Moughamian, A.J., and Holzbaur, E.L.F. (2014). Axonal transport: cargo-specific mechanisms of motility and regulation. Neuron *84*, 292–309.

Madisen, L., Zwingman, T.A., Sunkin, S.M., Oh, S.W., Zariwala, H.A., Gu, H., Ng, L.L., Palmiter, R.D., Hawrylycz, M.J., Jones, A.R., et al. (2010). A robust and high-throughput Cre reporting and characterization system for the whole mouse brain. Nat. Neurosci. *13*, 133–140.

Matsuda, W., Furuta, T., Nakamura, K.C., Hioki, H., Fujiyama, F., Arai, R., and Kaneko, T. (2009). Single nigrostriatal dopaminergic neurons form widely spread and highly dense axonal arborizations in the neostriatum. J. Neurosci. *29*, 444–453.

Mazaré, N., Oudart, M., Moulard, J., Cheung, G., Tortuyaux, R., Mailly, P., Mazaud, D., Bemelmans, A.-P., Boulay, A.-C., Blugeon, C., et al. (2020). Local translation in perisynaptic astrocytic processes is specific and changes after fear conditioning. Cell Rep. *32*, 108076.

Melia, K.R., Trembleau, A., Oddi, R., Sanna, P.P., and Bloom, F.E. (1994). Detection and regulation of tyrosine hydroxylase mRNA in catecholaminergic terminal fields: possible axonal compartmentalization. Exp. Neurol. *130*, 394–406.

Mendez, J.A., Bourque, M.-J., Fasano, C., Kortleven, C., and Trudeau, L.-E. (2011). Somatodendritic dopamine release requires synaptotagmin 4 and 7 and the participation of voltage-gated calcium channels. J. Biol. Chem. *286*, 23928–23937.

Mingote, S., Chuhma, N., Kalmbach, A., Thomsen, G.M., Wang, Y., Mihali, A., Sferrazza, C., Zucker-Scharff, I., Siena, A.-C., Welch, M.G., et al. (2017). Dopamine neuron dependent behaviors mediated by glutamate cotransmission. ELife *6*, e27566.

Nelson, E.L., Liang, C.L., Sinton, C.M., and German, D.C. (1996). Midbrain dopaminergic neurons in the mouse: computer-assisted mapping. J. Comp. Neurol. *369*, 361–371.

Nirenberg, M.J., Vaughan, R.A., Uhl, G.R., Kuhar, M.J., and Pickel, V.M. (1996a). The dopamine transporter is localized to dendritic and axonal plasma membranes of nigrostriatal dopaminergic neurons. J. Neurosci. *16*, 436–447.

Nirenberg, M.J., Chan, J., Liu, Y., Edwards, R.H., and Pickel, V.M. (1996b). Ultrastructural localization of the vesicular monoamine transporter-2 in midbrain dopaminergic neurons: potential sites for somatodendritic storage and release of dopamine. J. Neurosci. *16*, 4135–4145.

Nunes, I., Tovmasian, L.T., Silva, R.M., Burke, R.E., and Goff, S.P. (2003). Pitx3 is required for development of substantia nigra dopaminergic neurons. PNAS *100*, 4245–4250.

Oh, S.W., Harris, J.A., Ng, L., Winslow, B., Cain, N., Mihalas, S., Wang, Q., Lau, C., Kuan, L., Henry, A.M., et al. (2014). A mesoscale connectome of the mouse brain. Nature *508*, 207–214.

Omelchenko, N., and Sesack, S.R. (2009). Ultrastructural analysis of local collaterals of rat ventral tegmental area neurons: GABA phenotype and synapses onto dopamine and GABA cells. Synapse *63*, 895–906.

Ouwenga, R., Lake, A.M., Aryal, S., Lagunas, T., and Dougherty, J.D. (2018). The differences in local translatome across distinct neuron types is mediated by both baseline cellular differences and post-transcriptional mechanisms. ENeuro *5*, ENEURO.0320–18.2018.

Ouwenga, R., Lake, A.M., O'Brien, D., Mogha, A., Dani, A., and Dougherty, J.D. (2017). Transcriptomic analysis of ribosome-bound mRNA in cortical neurites in vivo. J. Neurosci. *37*, 8688–8705.

Ostroff, L.E., Santini, E., Sears, R., Deane, Z., Kanadia, R.N., LeDoux, J.E., Lhakhang, T., Tsirigos, A., Heguy, A., and Klann, E. (2019). Axon TRAP reveals learning-associated alterations in cortical axonal mRNAs in the lateral amygdala. Elife *8*, e51607.

Paget-Blanc, V., Pfeffer, M.E., Pronot, M., Lapios, P., Angelo, M.-F., Walle, R., Cordelières, F.P., Levet, F., Claverol, S., Lacomme, S., et al. (2021). Synaptomic analysis of dopaminergic inputs reveal hub synapses in the mouse striatum. BioRxiv, 2020.02.18.952978.

Palay, S.L., and Palade, G.E. (1955). The fine structure of neurons. J. Biophys. Biochem. Cytol. *1*, 69–88.

Papathanou, M., Dumas, S., Pettersson, H., Olson, L., and Wallén-Mackenzie, Å. (2019). Off-target effects in transgenic mice: characterization of dopamine transporter (DAT)-Cre transgenic mouse lines exposes multiple non-dopaminergic neuronal clusters available for selective targeting within limbic neurocircuitry. ENeuro *6*, ENEURO.0198–19.2019.

Pereira, D.B., Schmitz, Y., Mészáros, J., Merchant, P., Hu, G., Li, S., Henke, A., Lizardi-Ortiz, J.E., Karpowicz, R.J., Morgenstern, T.J., et al. (2016). Fluorescent false neurotransmitter reveals functionally silent dopamine vesicle clusters in the striatum. Nat. Neurosci. *19*, 578–586.

Perez, J.D., tom Dieck, S., Alvarez-Castelao, B., Tushev, G., Chan, I.C., and Schuman, E.M. (2021). Subcellular sequencing of single neurons reveals the dendritic transcriptome of GABAergic interneurons. ELife *10*, e63092.

Poulin, J.-F., Zou, J., Drouin-Ouellet, J., Kim, K.-Y.A., Cicchetti, F., and Awatramani, R.B. (2014). Defining midbrain dopaminergic neuron diversity by single-cell gene profiling. Cell Rep. *9*, 930–943.

Poulin, J.-F., Gaertner, Z., Moreno-Ramos, O.A., and Awatramani, R. (2020). Classification of midbrain dopamine neurons using single-cell gene expression profiling approaches. Trends Neurosci. *43*, 155–169.

Prensa, L., and Parent, A. (2001). The nigrostriatal pathway in the rat: a single-axon study of the relationship between dorsal and ventral tier nigral neurons and the striosome/matrix striatal compartments. J. Neurosci. *21*, 7247–7260.

Raman, I.M., and Bean, B.P. (1999). Ionic currents underlying spontaneous action potentials in isolated cerebellar Purkinje neurons. J. Neurosci. *19*, 1663–1674.

Ratai, O., Schirra, C., Rajabov, E., Brunk, I., Ahnert-Hilger, G., Chitirala, P., Becherer, U., Stevens, D.R., and Rettig, J. (2019). An alternative exon of CAPS2 influences catecholamine loading into LDCVs of chromaffin cells. J. Neurosci. *39*, 18–27.

Rayport, S., Sulzer, D., Shi, W.X., Sawasdikosol, S., Monaco, J., Batson, D., and Rajendran, G. (1992). Identified postnatal mesolimbic dopamine neurons in culture: morphology and electrophysiology. J. Neurosci. *12*, 4264–4280.

Rice, M.E., and Patel, J.C. (2015). Somatodendritic dopamine release: recent mechanistic insights. Philos. Trans. R. Soc. Lond. B, Biol. Sci. *370*, 20140185.

Richards, C.D., Shiroyama, T., and Kitai, S.T. (1997). Electrophysiological and immunocytochemical characterization of GABA and dopamine neurons in the substantia nigra of the rat. Neuroscience *80*, 545–557.

Robinson, B.G., Cai, X., Wang, J., Bunzow, J.R., Williams, J.T., and Kaeser, P.S. (2019). RIM is essential for stimulated but not spontaneous somatodendritic dopamine release in the midbrain. Elife *8*, e47972.

Roy, S. (2014). Seeing the unseen: the hidden world of slow axonal transport. Neuroscientist *20*, 71–81.

Sadakata, T., Mizoguchi, A., Sato, Y., Katoh-Semba, R., Fukuda, M., Mikoshiba, K., and Furuichi, T. (2004). The secretory granule-associated protein CAPS2 regulates neurotrophin release and cell survival. J. Neurosci. *24*, 43–52.

Sadakata, T., Itakura, M., Kozaki, S., Sekine, Y., Takahashi, M., and Furuichi, T. (2006). Differential distributions of the Ca2+ -dependent activator protein for secretion family proteins (CAPS2 and CAPS1) in the mouse brain. J. Comp. Neurol. *495*, 735–753.

Sakers, K., Lake, A.M., Khazanchi, R., Ouwenga, R., Vasek, M.J., Dani, A., and Dougherty, J.D. (2017). Astrocytes locally translate transcripts in their peripheral processes. Proc. Natl. Acad. Sci. U.S.A. *114*, E3830–E3838.

Sanz, E., Yang, L., Su, T., Morris, D.R., McKnight, G.S., and Amieux, P.S. (2009). Cell-type-specific isolation of ribosome-associated mRNA from complex tissues. Proc. Natl. Acad. Sci. U.S.A. *106*, 13939–13944.

Saunders, A., Macosko, E., Wysoker, A., Goldman, M., Krienen, F., de Rivera, H., Bien, E., Baum, M., Wang, S., Goeva, A., et al. (2018). Molecular diversity and specializations among the cells of the adult mouse brain. Cell *174*, 1015–1030.e16.

Scarnati, M.S., Kataria, R., Biswas, M., and Paradiso, K.G. (2018). Active presynaptic ribosomes in the mammalian brain, and altered transmitter release after protein synthesis inhibition. Elife *7*, e36697.

Shigeoka, T., Jung, H., Jung, J., Turner-Bridger, B., Ohk, J., Lin, J.Q., Amieux, P.S., and Holt, C.E. (2016). Dynamic axonal translation in developing and mature visual circuits. Cell *166*, 181–192.

Shigeoka, T., Koppers, M., Wong, H.H.-W., Lin, J.Q., Cagnetta, R., Dwivedy, A., de Freitas Nascimento, J., van Tartwijk, F.W., Ströhl, F., Cioni, J.-M., et al. (2019). On-site ribosome remodeling by locally synthesized ribosomal proteins in axons. Cell Rep. *29*, 3605–3619.e10.

Silbergeld, E.K., and Walters, J.R. (1979). Synaptosomal uptake and release of dopamine in substantia nigra: effects of γ-aminobutyric acid and substance P. Neurosci. Lett. *12*, 119–126.

Snyder, M.E., Finlayson, M.O., Connors, T.J., Dogra, P., Senda, T., Bush, E., Carpenter, D., Marboe, C., Benvenuto, L., Shah, L., et al. (2019). Generation and persistence of human tissue-resident memory T cells in lung transplantation. Sci. Immunol. *4*, eaav5581.

Subramanian, A., Tamayo, P., Mootha, V.K., Mukherjee, S., Ebert, B.L., Gillette, M.A., Paulovich, A., Pomeroy, S.L., Golub, T.R., Lander, E.S., et al. (2005). Gene set enrichment analysis: a knowledge-based approach for interpreting genome-wide expression profiles. PNAS *102*, 15545–15550.

Sulzer, D. (2007). Multiple hit hypotheses for dopamine neuron loss in Parkinson's disease. Trends Neurosci. *30*, 244–250.

Taylor, A.M., Berchtold, N.C., Perreau, V.M., Tu, C.H., Li Jeon, N., and Cotman, C.W. (2009). Axonal mRNA in uninjured and regenerating cortical mammalian axons. J. Neurosci. *29*, 4697–4707.

Tepper, J.M., Sawyer, S.F., and Groves, P.M. (1987). Electrophysiologically identified nigral dopaminergic neurons intracellularly labeled with HRP: light-microscopic analysis. J. Neurosci. *7*, 2794–2806.

Tinevez, J.-Y., Perry, N., Schindelin, J., Hoopes, G.M., Reynolds, G.D., Laplantine, E., Bednarek, S.Y., Shorte, S.L., and Eliceiri, K.W. (2017). TrackMate: an open and extensible platform for single-particle tracking. Methods *115*, 80–90.

Turiault, M., Parnaudeau, S., Milet, A., Parlato, R., Rouzeau, J.-D., Lazar, M., and Tronche, F. (2007). Analysis of dopamine transporter gene expression pattern – generation of DAT-iCre transgenic mice. FEBS J. *274*, 3568–3577.

Wang, F., Flanagan, J., Su, N., Wang, L.-C., Bui, S., Nielson, A., Wu, X., Vo, H.-T., Ma, X.-J., and Luo, Y. (2012). RNAscope: a novel in situ RNA analysis platform for formalin-fixed, paraffin-embedded tissues. J. Mol. Diagn. *14*, 22–29.

Wang, G., Ang, C.-E., Fan, J., Wang, A., Moffitt, J.R., and Zhuang, X. (2020). Spatial organization of the transcriptome in individual neurons. BioRxiv, 2020.12.07.414060.

Witkovsky, P., Patel, J.C., Lee, C.R., and Rice, M.E. (2009). Immunocytochemical identification of proteins involved in dopamine release from the somatodendritic compartment of nigral dopaminergic neurons. Neuroscience *164*, 488–496.

Wuytack, F., Papp, B., Verboomen, H., Raeymaekers, L., Dode, L., Bobe, R., Enouf, J., Bokkala, S., Authi, K.S., and Casteels, R. (1994). A sarco/endoplasmic reticulum Ca(2+)-ATPase 3-type Ca2+ pump is expressed in platelets, in lymphoid cells, and in mast cells. J. Biol. Chem. *269*, 1410–1416.

Xenias, H.S., Ibáñez-Sandoval, O., Koós, T., and Tepper, J.M. (2015). Are striatal tyrosine hydroxylase interneurons dopaminergic? J. Neurosci. *35*, 6584–6599.

Xie, Z., Bailey, A., Kuleshov, M.V., Clarke, D.J.B., Evangelista, J.E., Jenkins, S.L., Lachmann, A., Wojciechowicz, M.L., Kropiwnicki, E., Jagodnik, K.M., et al. (2021). Gene set knowledge discovery with Enrichr. Curr. Protoc. *1*, e90.

Younts, T.J., Monday, H.R., Dudok, B., Klein, M.E., Jordan, B.A., Katona, I., and Castillo, P.E. (2016). Presynaptic protein synthesis is required for long-term plasticity of GABA release. Neuron *92*, 479–492.

Zampese, E., and Surmeier, D.J. (2020). Calcium, bioenergetics, and Parkinson's disease. Cells *9*, 2045.

CellPress
OPEN ACCESS

STAR★METHODS

KEY RESOURCES TABLE

REAGENT or RESOURCE	SOURCE	IDENTIFIER
Antibodies		
Rabbit anti-HA	Abcam	ab9110, RRID:AB_307019
Biotinylated Rabbit anti-HA	Abcam	ab26228, RRID:AB_449023
Chicken anti-TH	Millipore	AB9702, RRID:AB_570923
Rabbit anti-RFP	Rockland	600-401-379, RRID:AB_2209751
Rabbit anti-ALDH1A1	Abcam	ab52492, RRID:AB_867566
Goat anti-Chicken IgY (H+L), Alexa Fluor Plus 488	ThermoFisher	A-32931TR, RRID:AB_2866499
Goat anti-Rabbit IgG (H+L), Alexa Fluor 647	ThermoFisher	A32733, RRID:AB_2633282
Goat anti-Rabbit IgG HRP	Vector Laboratories	PI-1000, RRID:AB_2336198
Mouse anti-HA	Cell Signaling	2367S, RRID:AB_10691311
Goat anti-Mouse HRP	Jackson ImmunoResearch	115-005-003, RRID:AB_2338447
Bacterial and Virus Strains		
AAV pCAG-FLEX-EGFP-WPRE	Addgene	51502, RRID:Addgene_51502
Chemicals, peptides, and recombinant proteins		
TSA Cy3	Perkin Elmer	NEL744001KT
TSA Cy5	Perkin Elmer	NEL745001KT
Critical commercial assays		
Mm-Slc6a3-C1	ACD Biotechne	315441
Mm-Th-C2	ACD Biotechne	317621-C2
Mm-Ddc-C3	ACD Biotechne	318681-C3
Mm-Slc18a2-C1	ACD Biotechne	425331
Mm-Dgkz-C1	ACD Biotechne	534861
Mm-Prkca-C2	ACD Biotechne	432261-C2
Mm-Homer2-O1	ACD Biotechne	581231
Mm-Atp2a3-C2	ACD Biotechne	1049861-C2
Mm-Cplx1-C3	ACD Biotechne	482531-C3
Mm-Snca-C1	ACD Biotechne	313281
Mm-Cadps2-C3	ACD Biotechne	529361-C3
Mm-Sv2c-C1	ACD Biotechne	545001
Mm-Rims1-C2	ACD Biotechne	539601-C2
Taqman qRT-PCR Assay: Mouse *ActB*	ThermoFisher	Mm01205647_g1
Taqman qRT-PCR Assay: Mouse *Th*	ThermoFisher	Mm00447557_m1
Taqman qRT-PCR Assay: Mouse *Slc6a3/DAT*	ThermoFisher	Mm00438388_m1
Taqman qRT-PCR Assay: Mouse *Slc18a2/VMAT2*	ThermoFisher	Mm00553058_m1
Taqman qRT-PCR Assay: Mouse *Gfap*	ThermoFisher	Mm01253033_m1
Taqman qRT-PCR Assay: Mouse *Mbp*	ThermoFisher	Mm01266402_m1
Taqman qRT-PCR Assay: *ERCC-0096*	ThermoFisher	Ac03460023_a1
Deposited Data		
DropViz scRNA-seq (Saunders et al., 2018)	GEO	GEO: GSE116470
RNA-seq data from this study	GEO	GEO: GSE180913

(*Continued on next page*)

Continued		
REAGENT or RESOURCE	SOURCE	IDENTIFIER
Experimental models: Organisms/strains		
Mouse: DAT$^{IRES\text{-}Cre}$: B6.SJL-*Slc6a3*$^{tm1.1(cre)Bkmn}$/J	Jackson Laboratories	JAX #006660, RRID: IMSR_JAX:006660
Mouse: Ai9: B6.Cg-*Gt(ROSA)26 Sor*$^{tm9(CAG\text{-}tdTomato)Hze}$/J	Jackson Laboratories	JAX #007909, RRID: IMSR_JAX:007909
Mouse: RiboTag: B6J.129(Cg)-*Rpl22*$^{tm1.1Psam}$/SjJ	Jackson Laboratories	JAX #029977, RRID: IMSR_JAX:029977
Mouse: DAT-Cre: Tg(Slc6a3-icre)1Fto	François Tronche, Université Pierre et Marie Curie	MGI:3770172, RRID: MGI:3770172
Mouse: VGLUT1^{VENUS}: Slc17a7$^{tm1.1Ehzg}$	Etienne Herzog, University of Bordeaux	RRID: 5297706
Software and algorithms		
RNA-seq data processing	GitHub	DropSeqPipeline8, https://github.com/simslab/DropSeqPipeline8
FISH analysis	GitHub	Neurite_FISH_Quant, https://github.com/simslab/Neurite_FISH_Quant

RESOURCE AVAILABILITY

Lead contact

Further information and requests for resources should be directed to and will be fulfilled by the Lead Contact, Peter Sims (pas2182@columbia.edu).

Materials availability

This study did not generate new unique reagents.

Data and code availability

- The RNA-seq data generated in this study are publicly available on the NIH Gene Expression Omnibus database (GEO: GSE180913). Raw count matrices and differential expression analysis output are provided as supplemental information.
- The Python and Shell code used for processing of RNA-seq data is accessible at: https://github.com/simslab/DropSeqPipeline8 (https://doi.org/10.5281/zenodo.5534458), and Python code for FISH analysis is accessible at: https://github.com/simslab/Neurite_FISH_Quant (https://doi.org/10.5281/zenodo.5570748).
- Any additional information required to reanalyze the data reported in this paper is available from the lead contact upon request.

EXPERIMENTAL MODEL AND SUBJECT DETAILS

Animals

All animals were housed in a 12-h/12h light/dark cycle with *ad libitum* access to food and water. DAT$^{IRES\text{-}Cre}$ mice (JAX #006660, RRID: IMSR_JAX:006660) (Bäckman et al., 2006), Ai9 mice (JAX #007909, RRID: IMSR_JAX:007909) (Madisen et al., 2010) and RiboTag mice (JAX #029977, RRID: IMSR_JAX:029977) (Sanz et al., 2009) were obtained from Jackson Laboratories. DAT-Cre mice (MGI:3770172, RRID: MGI:3770172) (Turiault et al., 2007) used in the FASS studies were a kind gift from Dr François Tronche. VGLUT1^{VENUS} mice (Slc17a7$^{tm1.1Ehzg}$, RRID: 5297706) used in the FASS studies have been previously described (Biesemann et al., 2014; Herzog et al., 2011).

Middle aged adult mice (10–14 months of age) of both sexes were used in most experiments unless otherwise noted, except for DA FASS studies, which used mature adult mice (3–6 months) of both sexes. For RiboTag experiments involving early postnatal ages (P0–P31), mice of both sexes were used and the exact ages are indicated in the text and figure captions. DAT$^{IRES\text{-}Cre}$:RiboTag experimental litters were bred by crossing homozygous RiboTag mice (RiboTag$^{+/+}$) with heterozygous DAT$^{IRES\text{-}Cre}$ (DAT$^{IRES\text{-}Cre/wt}$) mice, yielding litters of DAT$^{IRES\text{-}Cre/wt}$;RiboTag$^{+/-}$ (Cre-positive) and DAT$^{wt/wt}$;RiboTag$^{+/-}$ (Cre-negative) mice. Experimenters were blind to the genotype of mice in these litters throughout animal sacrifice and tissue dissection. Genotyping for the DAT$^{IRES\text{-}Cre}$ allele was conducted before biochemical experiments using established protocols (Bäckman et al., 2006). All experimental procedures were conducted according to National Institutes of Health guidelines and were approved by the Institutional Animal Care and Use

Committees of Columbia University and the New York State Psychiatric Institute, or according to the European guide for the care and use of laboratory animals and approved by the ethics committee of Bordeaux Universities (CE50) under the APAFIS #21132-2019061314534378v4 (CNRS, France).

METHOD DETAILS

Viral injections

As previously described (Paget-Blanc et al., 2021), Stereotaxic injections were performed in heterozygous DAT-*Cre*$^{+}$ mice of either sex at 8–9 weeks of age. An AAV1 pCAG-FLEX-EGFP-WPRE from the University of Pennsylvania core facility (Oh et al., 2014) was injected into DAT-*Cre*$^{+}$ mice. Saline-injected littermates were used as autofluorescence controls. The stereotaxic injections were performed in isoflurane-anesthetized mice using a 30-μL glass micropipette. Injection coordinates for the SNc were anterior/posterior (A/P), 3.6 mm; lateral (L), ±1.3 mm; and dorsal/ventral (D/V), 4.2 mm. Injection coordinates for the VTA were A/P, 3.16 mm, L, ±0.6 mm; and D/V, 4.2mm. The A/P and L coordinates are with respect to the *bregma*, whereas the D/V coordinates are given with respect to the brain surface. The animals were euthanized after 28 days at the maximal viral EGFP expression. For FASS experiments, four to six DAT-*Cre*$^{+}$ mice and one wild-type mouse were used.

Antibody use

Name	Use
Rabbit anti-HA	IHC, 1:1000
Chicken anti-TH	IHC, 1:500
	ICC, 1:1000
Rabbit anti-RFP	IHC, 1:500
Rabbit anti-ALDH1A1	IHC, 1:500
Goat anti-chicken IgY (H+L), Alexa Fluor Plus 488	IHC/ICC, 1:1000
Goat anti-rabbit IgG (H+L), Alexa Fluor 647	IHC/ICC, 1:1000
Goat anti-rabbit IgG HRP	IHC, 3:10,000
Mouse anti-HA	WB, 1:1000
Goat anti-mouse HRP	WB, 1:5000

See the Key resources table for manufacturer/catalog numbers. IHC, staining of acute brain slices or sections; ICC, staining of cultured neurons; WB, Western blotting.

Neuronal cultures

Ventral mesencephalic cultures containing dopaminergic neurons were prepared according to established procedures (Rayport et al., 1992). The VM (SN and VTA) from postnatal days 0–2 mice of either sex was dissected, dissociated, and plated on a monolayer of rat cortical astrocytes at the plating density of approximately 100,000 cells/cm^2. Experiments were conducted 14–21 d after plating.

Immunohistochemistry

Mice were anesthetized with euthasol and transcardially perfused with approximately 15 mL of 0.9% saline followed by 40–50 mL of ice-cold 4% paraformaldehyde (PFA) in 0.1 M phosphate buffer (PB), pH 7.4. Brains were post-fixed in 4% PFA in 0.1M PB for 6–12 h at 4°C, washed three times in phosphate-buffered saline (PBS), and sectioned at 50 μm on a Leica VT1000S vibratome. Sections were placed in cryoprotectant solution (30% ethylene glycol, 30% glycerol, 0.1M PB, pH 7.4) and stored at −20°C until further use.

Sections were removed from cryoprotectant solution and washed three times in tris-buffered saline (TBS) at room temperature. Sections were then permeabilized in TBS + 0.2% Triton-X 100 for 1 h at room temperature, followed by blocking in TBS + 10% normal goat serum (NGS) and 0.3% Triton-X 100 for 1.5 h at room temperature. Sections were then directly transferred to a prechilled solution containing primary antibodies in TBS + 2% NGS + 0.1% Triton-X 100 and incubated for approximately 40 h at 4°C. Sections were washed in TBS + 0.05% Tween 20 (TBS+T) five times over 1 h at room temperature. Sections undergoing tyramide signal amplification were treated with 3% hydrogen peroxide in TBS + T for 15 min at room temperature, followed by another two washes in TBS + T. Sections were incubated in a solution containing secondary antibodies in TBS + 2% NGS + 0.1% Triton-X 100 at room temperature for 1.5 h, followed by four washes in TBS + T over 45 min at room temperature. Sections undergoing tyramide signal amplification were then incubated in TSA-Cy5 (Perkin Elmer; 1:7500) in the manufacturer's diluent buffer for 1 h at room temperature. After four additional washes in TBS, sections were slide mounted and coverslipped with Fluoromount G (Southern Biotech). See Antibody use for a complete list of antibodies and concentrations used in this study.

Tissue dissection for RiboTag IP

Mice were sacrificed by cervical dislocation and brains were rapidly extracted and submerged in ice-cold 0.32 M sucrose buffer with 5 mM HEPES pH 7.4, 10 mM $MgCl_2$, and 100 μg/mL cycloheximide (CHX). Brains were placed on an ice-cold brain matrix (Zivic Instruments) and separated into 0.5- to 1.0-mm sections using ice cold razor blades. Striatum was dissected from slices between approximately –0.5 mm and 1.5 mm AP to Bregma. To avoid potential $DAT^{IRES\text{-}Cre}$ recombined cells in the lateral septum, a single vertical cut was made descending from the lateral ventricle on each side, and all medial tissue (including the lateral septum and nucleus accumbens shell) was discarded. The corpus callosum, cortex, and ventral olfactory tubercle were removed. The remaining dorsal and ventral striatum tissue was flash frozen on liquid nitrogen and stored at −80°C.

VM tissue was dissected from slices between approximately −2.5 mm and −3.75 mm AP to Bregma. First, the cortex, hippocampi, and any hypothalamus or white matter ventral to the midbrain were removed. For whole VM tissue dissections, a single horizontal cut was made just dorsal to the rostral linear nucleus and all dorsal tissue was discarded. The remaining tissue containing the SN/VTA was flash frozen on liquid nitrogen and stored at −80°C. For regional dissections, the SNr was first dissected away from the midbrain using a conservative semilunar cut halfway from the edge of the cerebral peduncle to the SNc (see Figure 2A). The remaining SNc tissue on either side was separated from the VTA by a vertical cut at the lateral edge of the VTA. All tissues were flash frozen and stored at −80°C.

Synaptosome preparation for RiboTag IP

VM or striatal dissections were homogenized in 1 mL of ice-cold 0.32 M sucrose with 5 mM HEPES pH 7.4, 10 mM $MgCl_2$, 100 μg/mL CHX, 1× EDTA-free protease inhibitors (Roche), and 100 U/mL SUPERaseIN. Nuclei and large debris were cleared at 2,000×*g* for 10 min at 4°C. The supernatant (S1) was further centrifuged at 7,000×*g* for 15 min at 4°C to yield the P2 pellet. The supernatant (S2) (cytoplasm and light membranes) was removed from the P2 pellet, which was washed by resuspension in 1 mL of ice-cold 0.32 M sucrose buffer (HEPES, $MgCl_2$, CHX, and inhibitors as above) and re-centrifuged at 10,000×*g* at 4°C before lysis. P2 pellets were lysed in 1 mL of lysis buffer (5 mM HEPES pH 7.4, 150 mM KCl, 10 mM $MgCl_2$, 1% Igepal CA-620, 100 μg/mL CHX, 1× EDTA-free protease inhibitors [Roche], and 100 U/mL SUPERaseIN). After resuspension, samples were incubated at 4°C on a rotor for 15 min. The resulting synaptosome lysate was subjected to RiboTag IP as described below.

Fluorescence-activated synaptosome sorting

Synaptosomes were prepared from the striatum or cortex of $VGLUT1^{venus}$ or DAT-Cre eGFP-expressing mice by homogenization in 1 mL of ice-cold isosmolar buffer (0.32 M sucrose, 4 mM HEPES pH7.4, protease inhibitor cocktail Set 3 EDTA-free [EMD Millipore Corp.]), using a 2 mL glass Teflon homogenizer with 12 strokes at 900 rpm. The homogenizer was rinsed with 250 μL of isosmolar buffer and three manual strokes and then, the pestle was rinsed with additional 250 μL of isosmolar buffer. The final 1.5 mL of homogenate (H) was centrifuged at 1000×*g* for 5 min at 4°C in a benchtop microcentrifuge. The supernatant (S1) was separated from the pellet (P1) and centrifuged at 12,600×*g* for 8 min at 4°C. The supernatant (S2) was discarded and the synaptosomes-enriched pellet (P2) was resuspended in 0.5 mL of isosmolar buffer and layered on a two-step Ficoll density gradient (900 μL of 7.5% and 900 μL of 13% Ficoll, 4 mM HEPES). The gradient was centrifuged at 50,000×*g* for 21 min at 4°C (Beckman Coulter Optima MAX XP ultracentrifuge with a TL-55 rotor). Sucrose synaptosomes were recovered at the 7.5/13% Ficoll interface using a 0.5-mL syringe.

Ficoll gradient-purified synaptosomes were diluted in PBS containing 1 μg /mL FM4-64 and stored on ice throughout the FASS procedures. The FACSAria-II (BD Biosciences) was operated with the following settings: 70 μm nozzle, sample shaking 300 rpm at 4°C, FSC neutral density filter 1.0, 488 nm laser on, area scaling 1.18, window extension 0.5, sort precision 0-16-0, FSC (340 V), SSC (488/10 nm, 365V), fluorescein isothiocyanta (enhanced green fluorescent protein) (530/30 nm, 700 V), PerCP (FM4-64) (675/20 nm, 700 V). Thresholding on FM4-64 was set with a detection threshold at 800. Samples were analyzed and sorted at rates of 15,000–20,000 events/s and flow rate of 3. Data were acquired using BD FACS DIVA 6. Cytometry plots were generated using FCS Express 7 (De Novo Software).

FISH

For mouse brain tissue and neuronal cultures, FISH was performed using the highly sensitive RNAScope Multiplex Fluorescent v2 assay (ACD Bio). See Antibodies and Reagents for a complete list of probes and reagents used in this study. Although most single FISH puncta using this assay are likely single mRNA molecules (Wang et al., 2012), this cannot be definitively determined owing to the enzymatic signal amplification and non-diffraction-limited size of the mRNA puncta.

Mouse brain sections were prepared as above, removed from cryoprotectant solution, and washed three times in TBS at room temperature. Sections were incubated with hydrogen peroxide (ACD) for 15 min at room temperature, washed several times in TBS, and then mounted to Superfrost slides (Fisher). Sections were allowed to dry for 10 min and a hydrophobic barrier (PAP pen, Vector Labs) was created around the tissue. Tissue was incubated in 50% EtOH, then 70% EtOH, then 100% EtOH for 5 min each. Sections were rehydrated in TBS for several minutes, digested with Protease IV (ACD) for 25 min at room temperature, and rinsed twice with TBS before proceeding to the RNA Scope Multiplex Fluorescent v2 assay (ACD).

Neuronal cultures were fixed in 4% PFA in 0.1 M PB + 4% sucrose for 10 min at room temperature. After several washes in TBS, the dish was filled with methanol pre-chilled to −20°C. Cultures were stored at −20°C for up to 4 weeks before FISH. After allowing

cultures to come to room temperature, methanol was replaced with 70% EtOH at room temperature for 2 min, then with 50% EtOH for 2 min, and then cultures were washed for 10 min in TBS. Cultures were treated with hydrogen peroxide (ACD) for 10 min at room temperature, followed by Protease III (ACD) diluted 1:15 in TBS for 10 min at room temperature, followed by two rinses in TBS before proceeding to the RNA Scope Multiplex Fluorescent v2 assay.

The RNA Scope Multiplex Fluorescent v2 assay was conducted according to the manufacturer's instructions, with all incubations taking place in a humidified chamber at 40°C. Two 5- min washes in excess RNA Scope Wash Buffer (ACD) took place between each incubation in sequential order: probes (2 h), AMP1 (30 min), AMP2 (30 min), AMP3 (15 min), HRP-C1/2/3 (15 min), TSA Cy3 (1:1500, 30 min), HRP blocker (30 min), HRP-C1/2/3 (15 min), and TSA Cy5 (1:1500, 30 min). Samples were washed twice more in RNA Scope Wash Buffer, then twice more in TBS. Samples were then blocked and immunostained for TH as described above. After immunostaining, samples were mounted in Fluoromount G and stored at 4°C for up to 1 week before imaging.

RiboTag ribosome IP

A detailed protocol is available online at protocols.io (https://doi.org/10.17504/protocols.io.by37pyrn): https://doi.org/10.17504/protocols.io.by37pyrn.

Frozen tissues were thawed on ice in a glass-glass dounce homogenizer with 1–1.5 mL of ice-cold lysis buffer (20 mM HEPES pH 7.4, 150 mM KCl, 10 mM MgCl2, 0.5 mM DTT, 100 μg/mL CHX, 1× EDTA-free protease inhibitors [Roche], and 100 U/mL SUPERaseIN). Tissues were lysed on ice using 30 strokes each with A and B pestles. Lysates were transferred to pre-chilled Eppendorf tubes and centrifuged at 1,000×*g* 4°C for 10 min, after which the supernatant was transferred to a new tube. One-ninth of the volume of 10% Igepal CA-630 was added to the lysates (final concentration 1%) and they were rotated at 4°C for 15 min. Lysates were clarified by centrifuging at 20,000×*g* 4°C for 10 min and transferred to a new tube. Five percent of the lysate was reserved as Input and frozen at −80°C.

We then added 1.5 μg (for striatal samples) or 6 μg (for midbrain samples) of biotinylated rabbit anti-HA and the lysates were rotated overnight at 4°C. Compared with previous protocols using Protein G Dynabeads, we found that biotinylated anti-HA IgG and streptavidin T1 Dynabeads enabled rapid binding with higher specificity (Figure S1H). Streptavidin T1 Dynabeads (ThermoFisher, catalog #65601) were then added to the lysates (5 μL/μg of biotinylated antibody) and rotated for 30 min at 4°C. Beads were captured on a magnetic rack and the lysate was discarded. Beads were resuspended in 500 μL of ice-cold high salt buffer (20 mM HEPES, pH 7.4, 350 mM KCl, 10 mM MgCl2, 1% Igepal CA-630, 0.5 mM DTT, 100 μg/mL CHX, 1× EDTA-free protease inhibitors [Roche], and 100 U/mL SUPERaseIN) and transferred to a new tube. Beads were rotated for 30 min at 4°C, then captured on a magnetic rack and washed again three more times with ice-cold high salt buffer (four washes total over 2 h). After the last wash, beads were resuspended in 100 μL of ribosome release buffer (20 mM HEPES, pH 7.4, 50 mM EDTA, 100 U/mL SUPERaseIN) and incubated for 10 min at room temperature. Beads were captured on a magnetic rack and the eluate containing the released mRNA was transferred to a new tube. Beads (with eL22-HA still bound) were flash frozen on liquid nitrogen and stored at −80°C. The aqueous mRNA eluate was purified using the RNEasy MinElute kit (Qiagen, catalog #74204) according to the manufacturer's instructions. RNA was eluted in 14 μL of nuclease free water supplemented with 20 U/mL SUPERaseIN and stored at −80°C.

Quantitative RT-PCR

For RiboTag IP samples, an equal fraction of captured RNA was reverse transcribed (1.5 μL of the 14 μL elution from RNEasy MinElute purification). For Input or other tissue samples, 20–50 ng of total RNA was reverse transcribed. RNA was reverse transcribed in a 20-μL reaction with 0.5 U of Maxima H Reverse Transcriptase (ThermoFisher, catalog # EP0753) and random hexamers (5 μM, ThermoFisher catalog #SO142).

A quantitative PCR was run with TaqMan Universal Master Mix (ThermoFisher catalog #4440042) and TaqMan FAM-MGB primer/probe sets spanning exon junctions on a BioRad CFX96. The following primer/probe sets were used (ThermoFisher): Mouse *ActB*, Mm01205647_g1; Mouse *Th*, Mm00447557_m1; Mouse *Slc6a3/DAT*, Mm00438388_m1; Mouse *Slc18a2/VMAT2*, Mm00553058_m1; Mouse *Gfap*, Mm01253033_m1; Mouse *Mbp*, Mm01266402_m1; and *ERCC-0096*, Ac03460023_a1. For RiboTag IP samples, an equal fraction of cDNA was used in each reaction. For Input or other tissue samples, 3–5 ng cDNA was used in each reaction.

Western blotting

Frozen Streptavidin T1 Dynabeads were thawed and resuspended in 1× LDS sample buffer supplemented with 20 mM DTT. To elute eL22-HA, beads were boiled at 95°C for 5 min and then placed onto a magnetic rack. Samples were loaded into 10% Bis-Tris polyacrylamide gels (Invitrogen, ThermoFisher catalog #NP0303BOX) and transferred to PVDF membranes (Immobilon-P, MilliporeSigma, catalog #IPVH00010). Membranes were initially washed for 15 min in TNS with Tween (TBST) (1× TBS + 0.1% Tween 20), blocked for 1 h in 5% bovine serum albumin (BSA)/TBST, and incubated overnight at 4°C with primary antibody in 5% BSA/TBST overnight. After primary incubation, membranes were washed three times in TBST before incubation with horseradish peroxidase (HRP)-conjugated secondary antibody in 5% BSA/TBST for 1 h at room temperature. After secondary incubation, membranes were washed three times in TBST. Signal was developed using Immobilon enhanced chemiluminescent substrate (Millipore, catalog #WBKLS0500) and imaged on an Azure Biosystems C600 system.

Image acquisition

Widefield imaging of eL22-HA staining was conducted on a Nikon Ti2 Eclipse equipped with a SpectraX light engine (Lumencor) and a DS-Qi2 camera (Nikon), using a 10x/0.25 NA or 20x/0.75 NA air objectives (Nikon). Confocal imaging was conducted on a Leica SP8 laser scanning system using a 60×/1.45 NA objective (Leica).

eL22-HA image analysis

Z stack images from the SNr, MFB, and striatum were acquired using a 60×/1.45 NA oil-immersion objective on a Leica SP8 confocal microscope. The 10-μm depth sub-stacks were collapsed via maximum projection for downstream analysis. A binary mask was used to identify pixels in TH-positive dendrites and axons. The mean eL22-HA intensity for TH-positive pixels was subtracted from the mean eL22-HA intensity for all pixels within each field and is reported in Figure 1F as normalized eL22-HA mean fluorescence intensity.

FISH image analysis

RNA puncta were analyzed using TrackMate (Tinevez et al., 2017). The Laplacian of Gaussian spot detector with estimated blob diameter of 0.5–1.0 μm. Additional filtering was implemented using a combination of quality, contrast, and total intensity as necessary to suppress background spot detection. For each image, the centroid (X,Y,Z) coordinates, diameter, and other quantitative parameters of each punctum were exported for further analysis.

For the percent co-localization shown in Figure 4D, a binary threshold was set for the TH IF signal based on two standard deviations above the image background to generate a binary mask of pixels for TH^+ neurites. The 23 pixels surrounding the TrackMate centroid coordinate of each punctum were analyzed (3 × 3 × 3 cube of pixels excluding the four corner pixels) for overlap with the TH^+ neurite pixels. Puncta with more than 60% overlapping pixels were retained as co-localized within TH^+ neurites. The number of puncta co-localized within TH^+ neurites was divided by the total volume of TH^+ pixels in each field, yielding the puncta per volume of TH^+ neurite shown in Figure 4D.

For quantification of puncta per micrometer of dendrite shown in Figure 4F, individual dendrites were segmented using the SimpleNeuriteTracer plugin in ImageJ. Dendrites were filled in three dimensions and exported as a binary mask, from which the (X,Y,Z) coordinates of all pixels in each dendrite were extracted. TrackMate was run once on each original image file, and the number of puncta within each dendrite was determined using the same co-localization analysis as above. The number of puncta in each dendrite was divided by the path length of each dendrite from SimpleNeuriteTracer.

For the quantification of *Atp2a3* puncta per neuron shown in Figure 5G, individual mDA neuronal soma were segmented in maximum projections of 10 μm Z-stack images using the ImageJ magic wand tool on thresholded TH pixel intensities. Each soma was saved as a region of interest (ROI), and the (X,Y) coordinates of each ROI were exported. TrackMate was run once on each original image file, and the puncta within each soma were determined using the same co-localization analysis as above.

For the quantification of puncta per 10 μm of dendrite shown in Figure 6K, dendrites of cultured neurons were manually segmented using *Selection – Straighten* in ImageJ. TrackMate was run on each individual image file, and the number of puncta in each dendrite was divided its length.

Full-length total RNA-seq

Full-length total RNA-seq was conducted using the SMARTer Stranded Total RNA-seq Kit v3, Pico Input Mammalian (Takara Bio, catalog no. 634485). We used 1,000 pg of total RNA for Input samples. For RiboTag IP samples, an estimated 500–1,000 pg (via ActB qPCR) (see Figure S1I) was used. Libraries were constructed according to the manufacturer's instructions with the following parameters: (1) 4-min fragmentation before reverse transcription, and (2) 14–15 cycles of PCR after ZapR depletion. Unique dual indexes were assigned to each sample, and libraries were pooled at 1 nM after quantification using Qubit dsDNA HS and Agilent 2100 Bioanalyzer High Sensitivity DNA assays. Pooled libraries were sequenced on a NextSeq 500 with 2 × 75 bp paired end reads (HO 150 kit, Illumina).

The first 15 bp of Read 2 (UMI and TSO sequences) were removed using fastx-trimmer (http://hannonlab.cshl.edu/fastx_toolkit/index.html), and paired-end reads then were depleted of rRNA by alignment to mouse 5S, 5.8S, 18S, and 28S rRNA using bowtie2 (Langmead and Salzberg, 2012). rRNA-depleted paired-end reads were then aligned to the mouse genome (GENCODE M25, GRCm38.p6) using STAR 2.6.7a (Dobin et al., 2013). Uniquely mapped reads were then quantified at the exon level using featureCounts version 1.6 (Liao et al., 2014).

Low input RNA-seq with 96-well plate, pooled library construction

The protocol for plate-based, 3′ end unique molecular indicator (UMI)-based RNA-seq of single cells has been described previously (Snyder et al., 2019) and was further modified to accommodate ultra-low input RiboTag IP samples. See Data S1 for sequences of all custom primers and oligonucleotides used in this protocol. Briefly, an estimated 20–500 pg of total RNA (based on qPCR, see above) for each sample was loaded into the wells of a 96-well plate in a volume of 6 μL of nuclease-free water containing 1 U/μL SUPERaseIN (ThermoFisher). After adding 1.5 μL of 10 μM barcoded RT primer (Integrated DNA Technologies), primer annealing was performed at 72°C for 3 min. Reverse transcription was performed by adding 7.5 μL RT mix to each well (2.81 μL of 40% polyethylene glycol 8000, 0.15 μL of 100 mM dNTPs, 3 μL of 5X Maxima H RT Buffer, 0.2 μL of 200 U/μL Maxima H Reverse Transcriptase [ThermoFisher],

0.2 μL of 20 U/μL SUPERaseIN, 0.15 μL of 100 μM Template Switching Oligo [Integrated DNA Technologies], and 1 μL of nuclease-free water). Reverse transcription was performed at 42°C for 90 min, followed by 10 cycles of 50°C for 2 min, 42°C for 2 min, 75°C for 10 min, followed by a 4°C hold. Excess primers were removed by adding 2 μL of Exonuclease I mix (1.875U ExoI in water) to each well and incubating at 37°C for 30 min, 85°C for 15 min, 75°C for 30 s, 4°C hold.

All wells were pooled into a single 15-mL Falcon tubes and cDNA was purified and concentrated using Dynabeads MyOne Silane beads (ThermoFisher) according to the manufacturer's instructions. The cDNA was split into duplicate reactions containing 25 μL cDNA, 25 μL 2× HIFI HotStart Ready Mix (Kapa Biosystems), and 0.2 M SMART PCR Primer. PCR was run as follows: 37°C for 30 min, 85°C for 15 min, 75°C for 30 s, 4°C hold. Duplicate reactions were combined and purified using 0.7 volumes AMPure XP beads (Beckman Coulter). The amplified cDNA was visualized on an Agilent 2100 Bioanalyzer and quantified using a Qubit II fluorometer (ThermoFisher).

Sequencing libraries were constructed using Nextera XT (Illumina) with modifications. A custom i5 primer was used (NexteraPCR) with 0.6 ng input cDNA and 10 cycles of amplification was performed. Unique i7 indexes were used for each plate. After amplification, the library was purified with two rounds of AMPure XP beads, visualized on the Agilent 2100 Bioanalyzer and quantified using the Qubit II fluorometer. Libraries were sequenced on an Illumina NextSeq 500 using the 75 cycle High Output kit (read lengths 26(R1) × 8(i) × 58(R2)). Custom sequencing primers were used for Read 1 (SMRT_R1seq and ILMN_R1seq, see Antibodies and Reagents). With each plate we targeted approximately 400M reads. Library pools were loaded at 1.8 pM with 20% PhiX (Illumina).

Reads were aligned to the mouse reference genome GRCm38 and transcriptome annotation (Gencode vM10) using the STAR aligner with parameters *–sjdbOverhang 65 –twopassMode Basic* after trimming poly(A)-tails from the 3′-ends. The aligned reads were demultiplexed using the well-identifying barcodes, correcting all single-nucleotide errors. All reads with the same well-identifying barcode, UMI, and gene mapping were collapsed to represent an individual transcript. To correct for sequencing errors in UMIs, we further collapsed UMIs that were within Hamming distance one of another UMI with the same well-identifying barcode and gene. For each 96-well plate, after generating a final list of individual transcripts with unique combinations of well-identifying barcodes, UMIs, and gene mapping, we produced a molecular count matrix for downstream analysis.

Synaptosome mRNA content estimation

For UMI-based estimation of mRNAs per particle shown in Figure 3J, the extent to which the total UMIs per sorted particle under-estimates the number of mRNAs per sorted particle was modeled as a function of the efficiency of RNA extraction and reverse transcription:

$$\textit{Estimated mRNAs per Sorted Particle} = \frac{\textit{Total UMIs}}{\textit{Sorted Particles} \times \textit{Efficiency}}$$

Where *Efficiency* is in decimal form (i.e., 1% efficiency = 0.01, such that the *Estimated mRNAs per Sorted Particle* is 100-fold more than for 100% efficiency = 1).

For the estimation of mRNAs per particle based on total RNA measurement of forebrain VGLUT1^{venus} FASS samples (Hafner et al., 2019) shown in Figure 3J, the total RNA yield (1–5 ng) from 100 million sorted particles was converted to mRNA estimates as follows:

$$\textit{Estimated mRNAs per Sorted Particle} = \frac{\textit{Total RNA Yield}}{10^8 \textit{ sorted particles}} \times \frac{\%\, \textit{mRNA} \times 6.022 \times 10^{23}}{\textit{mRNA MW}}$$

Where *% mRNA* specifies the estimated mass percent of mRNA among total RNA in decimal form (typically 1%–5% or 0.01–0.05), and *mRNA MW* is the average molecular weight of a eukaryotic mRNA in g/mol = (2000 nt × 320.5 g/mol) + 159. In Figure 3J, the upper bound corresponds to *Total RNA Yield* = 5 ng and *% mRNA* = 0.05, while the lower bound corresponds with the *Total RNA Yield* = 1 ng and *% mRNA* = 0.01.

RNA-seq differential expression analysis

An analysis of RiboTag IP and Input sample UMI count matrices shown in Figures 3, 6, and S2 (from *Low input RNA-seq with 96-well plate*, *pooled library construction*) was conducted using a GLM in *DESeq2* (Love et al., 2014). The likelihood ratio test (LRT) was used to identify genes for which a given term contributed significantly to the likelihood of the full GLM compared with a GLM lacking the given term; that is, the LRT identifies genes for which a given term adds significant explanatory power to the GLM. The *DESeq2 dds* object was constructed with two- or three-factor models and their interaction terms as specified in the Results text. For example, in comparing the full model: ~*genotype* + *fraction* + *genotype:fraction* versus the reduced model: ~*genotype* + *fraction*, the p values report on whether the increased likelihood of the full model is greater than by chance if the genotype:fraction term truly has no explanatory power. For most LRTs, the log2 Fold Changes specify the contrast between the two levels of the factor (e.g., IP versus Input for fraction, Cre^{-} versus Cre^{+} for genotype). For interaction terms, the contrast specifies the difference in log2 fold change for the effect of one factor between the levels of the other factor (i.e., for genotype:fraction, the difference in IP versus Input between Cre^{-} and Cre^{+} samples). Note that the age factor in Figures 3A and 3B has multiple levels, and so the p values do not relate specifically to any single contrast. The log2 fold changes specified for the age LRT in Data S5 are for the contrast P90 versus P0.

A complete *DESeq2* summary for analyses related to the following figures is found in the corresponding supplemental data:

- Data S5 contains the *DESeq2* results for the bulk striatal RiboTag IP analysis related to Figure 3
- Data S5 contains the *DESeq2* results for the striatal synaptosome RiboTag IP analysis related to Figure S2
- Data S5 contains the *DESeq2* results for the FASS analysis related to Figure 3
- Data S7 contains the *DESeq2* results for the midbrain synaptosome RiboTag IP analysis related to Figure 6

An analysis of the midbrain RiboTag IP and Input samples in Figures 5 and S5 (from full-length total RNA-seq) was conducted in a GLM in *DESeq2*. The Wald test was used to make direct comparisons between specific IP samples (e.g., SNr IP vs. VTA IP) or between the IP and Input samples within each region (e.g., SNr IP vs. SNr Input). Downstream filtering of DEGs (false discovery rate [FDR] < 0.05) to remove non-specific mRNAs is summarized in Figure S5F. After the identification of DEGs in comparisons of SNr IP versus VTA or SNc RiboTag IPs, the intersection of SNr-enriched or SNr-depleted genes (relative to SNc/VTA) from these two DEG lists is retained. Next, only genes enriched in SNr IP versus Input or SNc/VTA IP versus Input comparisons were retained. Fourth, the yield of Cre^{-} IPs was too low for full-length RNA-seq (Figures S5D and S5E), so we used the same UMI-based RNA-seq protocol as above to identify mRNAs significantly enriched in midbrain Cre^{-} IP samples and removed them from subsequent analyses (Data S6). Genes that were significantly higher in Cre^{-} IP samples compared with Cre^{+} IP samples were removed (non-specific binders). A complete *DESeq2* summary for Figures 5 and S5 are found in Data S6.

GSEA and GO analysis

For all GO analyses, a single list of unique genes was used (i.e., DEGs from *DESeq2* analysis). The GO analyses shown in Figures 3H and 6G were conducted using web-based Enrichr (Xie et al., 2021) with 2018 GO Terms for Cellular Component, Biological Process, and Molecular Function (Ashburner et al., 2000; Gene Ontology Consortium, 2021). The synaptic GO analysis shown in Figure 6G was conducted using SynGO (Koopmans et al., 2019). The results shown in Figure 5E used GSEA (Subramanian et al., 2005) in pre-ranked mode, with the SNr versus SNc IP or SNr versus VTA IP *DESeq2* log2 fold change as the rank list and the top 50 markers of each cluster as the gene sets.

QUANTIFICATION AND STATISTICAL ANALYSIS

Unless otherwise noted, all statistical analysis and data visualization was conducted in Python 3.7.3 using *SciPy*, *scikit-learn*, *Statsmodels*, *Matplotlib*, and *Seaborn* packages. Statistical comparisons were conducted using Welch's unequal variance *t* tests, Mann-Whitney *U* tests, or two-way analysis of variance (ANOVA) with Tukey's HSD *post hoc* comparisons. The number of replicates and other statistical testing information indicated in the figure captions. Box and whiskers plots display the median, first, and third quartiles, with whiskers at the minimum or maximum values. Error bars on bar charts are standard error of the mean.

Cell Reports, Volume *38*

Supplemental information

Subcellular and regional localization of mRNA translation in midbrain dopamine neurons

Benjamin D. Hobson, Linghao Kong, Maria Florencia Angelo, Ori J. Lieberman, Eugene V. Mosharov, Etienne Herzog, David Sulzer, and Peter A. Sims

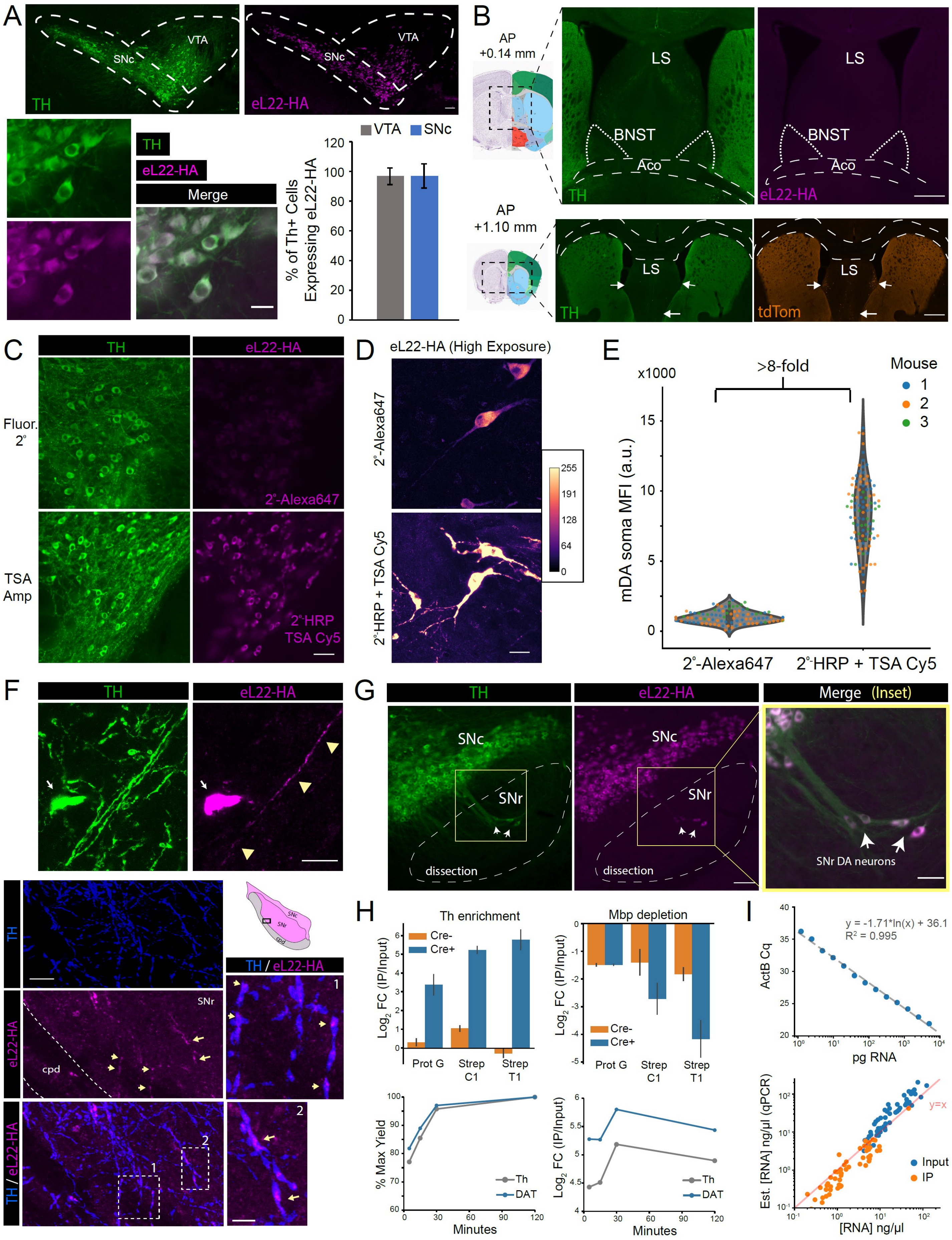

A
TH
eL22-HA
SNc
VTA
Merge
% of Th+ Cells Expressing eL22-HA
VTA
SNc
B
AP +0.14 mm
AP +1.10 mm
LS
BNST
Aco
tdTom
C
Fluor. 2°
TSA Amp
2°-Alexa647
2°HRP TSA Cy5
D
eL22-HA (High Exposure)
2°-Alexa647
2°HRP + TSA Cy5
E
x1000
>8-fold
Mouse
mDA soma MFI (a.u.)
2°-Alexa647
2°HRP + TSA Cy5
F
SNr
cpd
TH / eL22-HA
G
SNr
dissection
Merge (Inset)
SNr DA neurons
H
Th enrichment
Mbp depletion
Cre-
Cre+
Log2 FC (IP/Input)
Prot G
Strep C1
Strep T1
% Max Yield
Minutes
Th
DAT
I
y = -1.71*ln(x) + 36.1
R² = 0.995
ActB Cq
pg RNA
Est. [RNA] ng/μl (qPCR)
[RNA] ng/μl
y=x
Input
IP

Figure S1: Histological analysis of eL22-HA expression in DAT$^{IRES-Cre}$:RiboTag mice, eL22-HA signal amplification, eL22-HA staining in distal SNr dendrites of mDA neurons, RiboTag IP optimization, and qRT-PCR estimation of RiboTag IP yield, Related to Figure 1 and Figure 2

(A) *Upper*: Epifluorescence image of DAT$^{IRES-Cre}$:RiboTag midbrain immunostained for tyrosine hydroxylase (TH) and eL22-HA. Dashed lines indicate regions used for cell counting shown below. *Lower left*: SNc of DAT$^{IRES-Cre}$:RiboTag, representative of images for cell counting. Scale bar, 20 µm. *Lower right*: % of TH$^+$ cells expressing eL22-HA at indicated ages. Data represent mean ± standard deviation, and are derived from n=2-3 mice and n=6 fields, (VTA) n= 717 cells; (SNc) n=451 cells. Mean is >95% in both regions. Scale bar, 100 µm.

(B) *Upper*: DAT$^{IRES-Cre}$:RiboTag midbrain stained for TH and eL-22HA at the indicated coordinates. *Lower*: DAT$^{IRES-Cre}$:Ai9 section stained for TH and tdTomato at indicated coordinates. Both scale bars, 500 µm.

(C) Comparison of AlexaFluor647-conjugated secondary antibody vs. TSA-Cy5 + HRP-conjugated secondary antibody for eL22-HA immunostaining. Exposure was optimized to avoid saturation of soma. Scale bar, 50 µm.

(D) Same as panel A, but using high exposure to detect dendritic labeling (somata are saturated for TSA-Cy5). Scale bar, 25 µm.

(E) Quantification of somata eL22-HA intensity, related to panel A. Violin plot depicts n=100 soma quantified from 2-3 fields each from 3 mice.

(F) *Upper*: eL22-HA labeling in descending SNr dendrites can be distinguished from soma of SNr mDA neurons. Scale bar, 20 µm. *Lower*: Amplified eL22-HA labeling is observed in distal SNr mDA neuronal dendrites near the cerebral peduncle. Scale bar, 15 µm. Inset scale bar, 5 µm.

(G) DAT$^{IRES-Cre}$:RiboTag midbrain immunostained for TH and eL22-HA. Scale bar, 200 µm. *Arrows*: A few scattered Th+/eL22-HA+ mDA neurons are present in the SNr. Scale bar, 50 µm.

(H) Optimization of eL22-HA IP conditions. *Upper*: Cre-negative or Cre-positive ventral midbrain (n=2 each genotype) lysate was split into three equal parts for capture with Rabbit anti-HA (Protein G beads) or biotinylated Rabbit anti-HA (Streptavidin C1/T1 beads). Mean and confidence intervals are plotted. *Lower*: Further optimization of capture time using four equal parts of DAT$^{IRES-Cre}$:RiboTag ventral midbrain lysate with biotinylated Rb anti-HA and Streptavidin T1 beads. Antibody was incubated with polysome lysates overnight, and beads were added for the capture time indicated.

(I) *Upper:* qRT-PCR of beta-Actin (ActB) with total RNA input amounts (measured by Qubit) ranging from 1 pg – 5 ng. *Lower:* qRT-PCR (ActB) estimation of RNA concentration (measured by Qubit for Input samples, or RNA Pico bioanalyzer for IP samples, n=40-48 each).

Abbreviations: SNc, Substantia nigra pars compacta; SNr, Substantia nigra parts reticulata; VTA, Ventral tegmental area, Aco, Anterior commissure; LS, Lateral septum; BNST, Bed nucleus of the stria terminalis, TSA; tyramide signal amplification, HRP; horseradish peroxidase, cpd; cerebral peduncle, Th; tyrosine hydroxylase, Mbp; myelin basic protein, DAT; dopamine transporter, ActB; beta-actin.

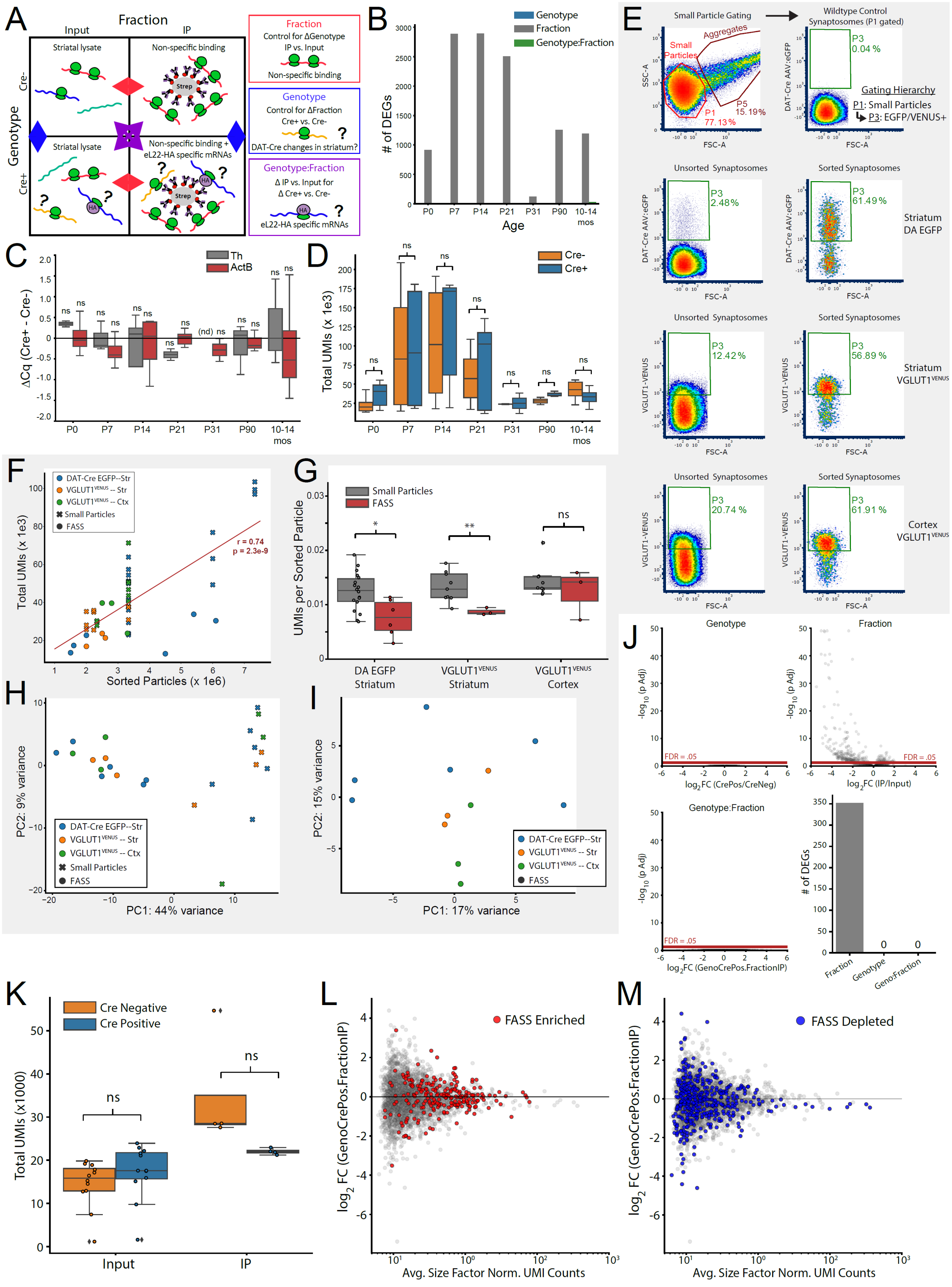

A
Fraction
Input
IP
Striatal lysate
Non-specific binding
Cre-
Genotype
Cre+
Striatal lysate
Non-specific binding + eL22-HA specific mRNAs
Strep
HA
Fraction
Control for ΔGenotype
IP vs. Input
Non-specific binding
Genotype
Control for ΔFraction
Cre+ vs. Cre-
DAT-Cre changes in striatum?
Genotype:Fraction
Δ IP vs. Input for
Δ Cre+ vs. Cre-
eL22-HA specific mRNAs
B
Genotype
Fraction
Genotype:Fraction
# of DEGs
P0
P7
P14
P21
P31
P90
10-14 mos
Age
C
Th
ActB
ΔCq (Cre+ - Cre-)
ns
(nd)
P0
P7
P14
P21
P31
P90
10-14 mos
D
Cre-
Cre+
Total UMIs (x 1e3)
ns
P0
P7
P14
P21
P31
P90
10-14 mos
E
Small Particle Gating
Aggregates
Small Particles
P1 77.13%
P5 15.19%
SSC-A
FSC-A
Wildtype Control
Synaptosomes (P1 gated)
DAT-Cre AAV:eGFP
P3 0.04 %
Gating Hierarchy
P1: Small Particles
P3: EGFP/VENUS+
Unsorted Synaptosomes
P3 2.48%
Sorted Synaptosomes
P3 61.49 %
Striatum DA EGFP
Unsorted Synaptosomes
VGLUT1-VENUS
P3 12.42%
Sorted Synaptosomes
P3 56.89 %
Striatum VGLUT1 VENUS
Unsorted Synaptosomes
P3 20.74 %
Sorted Synaptosomes
P3 61.91 %
Cortex VGLUT1 VENUS
F
DAT-Cre EGFP--Str
VGLUT1 VENUS -- Str
VGLUT1 VENUS -- Ctx
Small Particles
FASS
r = 0.74
p = 2.3e-9
Total UMIs (x 1e3)
Sorted Particles (x 1e6)
G
Small Particles
FASS
UMIs per Sorted Particle
*
**
ns
DA EGFP Striatum
VGLUT1 VENUS Striatum
VGLUT1 VENUS Cortex
H
PC2: 9% variance
PC1: 44% variance
DAT-Cre EGFP--Str
VGLUT1 VENUS -- Str
VGLUT1 VENUS -- Ctx
Small Particles
FASS
I
PC2: 15% variance
PC1: 17% variance
DAT-Cre EGFP--Str
VGLUT1 VENUS -- Str
VGLUT1 VENUS -- Ctx
FASS
J
Genotype
Fraction
-log10 (p Adj)
FDR = .05
log2FC (CrePos/CreNeg)
log2FC (IP/Input)
Genotype:Fraction
log2FC (GenoCrePos.FractionIP)
# of DEGs
0
0
Fraction
Genotype
Geno:Fraction
K
Cre Negative
Cre Positive
Total UMIs (x1000)
ns
ns
Input
IP
L
FASS Enriched
log2 FC (GenoCrePos.FractionIP)
Avg. Size Factor Norm. UMI Counts
M
FASS Depleted
log2 FC (GenoCrePos.FractionIP)
Avg. Size Factor Norm. UMI Counts

Figure S2: RNA-Seq analysis of bulk striatal and striatal synaptosome RiboTag IP samples, VGLUT1^{VENUS} and DA:EGFP FASS Gating, and RNA-Seq analysis of FASS samples, Related to Figure 3

(A) Schematic depicting generalized linear model analysis of striatal RiboTag IP using the *DESeq2* likelihood ratio test (LRT). Analogous to a 2-way analysis of variance, the main effects of each factor and the interaction between them are each tested for statistical significance across all genes (with FDR correction). See **Methods**.

(B) Number of genes meeting statistical significance (FDR < 0.05) in the *DESeq2* LRT with the indicated terms omitted from the following reduced model: ~*genotype* + *fraction* + *genotype:fraction*. In addition to testing each age was independently (*shown here*), the effects of *age*, *fraction*, *genotype*, and *fraction:genotype* interaction were also on the entire dataset across all ages (see **Figure 3A-B**). In contrast to the genotype-independent effect of *fraction* (non-specific binding), the effects of *genotype* and *genotype:fraction* interaction were negligible at all ages. See **Supplementary File 5** for complete summary of *DESeq2* analysis. Mice per age and genotype: (P0, Cre-) n= 6, (P0, Cre+) n=6, (P7, Cre-) n=6, (P7, Cre+) n=6, (P14, Cre-) n=6, (P14, Cre+) n=6, (P21, Cre-) n=6, (P21, Cre+) n=7, (P31, Cre-) n=2, (P31, Cre+), n=2, (P90, Cre-) n=2, (P90, Cre+), n=3, (10-14 mo., Cre-) n=6, (10-14 mo., Cre+) n=4, where each n indicates an IP and corresponding Input sample.

(C) qRT-PCR measurement of *Th* and *ActB* mRNA yield in striatal RiboTag IPs from Cre-negative and Cre-positive mice. The difference between the average Cq values (Cre-positive Cq – Cre-negative Cq) is plotted at each age. Mice per age and genotype are the same as in panel B. nd: not detectable, ns: all comparisons are not significant (p > 0.05), Welch's unequal variance t-test.

(D) Total UMIs per sample for striatal RiboTag IPs from Cre-negative and Cre-positive mice. Mice per age and genotype are the same as in panel B. ns: all comparisons are not significant (p > 0.05), Welch's unequal variance t-test.

(E) Density plots of Fluorescence-Activated Synaptosome Sorting (FASS) gating strategy. <u>*Upper*</u>: Particles are first selected from the 'P1' gate on forward and side scatter in order to avoid aggregates. Synaptosomes from wildtype controls are used to set a fluorescence threshold on which to sort VGLUT1^{VENUS} and DA:EGFP particles. <u>*Lower*</u>: Representative density plots of Unsorted (*left*) and Sorted (*right*) synaptosomes from the indicated regions and genotypes.

(F) Number of sorted particles vs. total UMIs for the indicated sorted samples (n=6 striatum DA:EGFP, n=3 striatum VGLUT1^{VENUS}, n=3 cortex VGLUT1^{VENUS}, where each n represents both a FASS sample and three corresponding small particle samples). Pearson's r = 0.74, p = 2.3e-09.

(G) Total UMIs per sorted particle for small particle and FASS samples as indicated. * indicates p < 0.05, ** indicates p < 0.01, Welch's unequal variance t-test. Samples are the same as shown in panel F.

(H) PCA of *DESeq2 rlog* normalized UMI counts for FASS and small particle samples. Samples are the same as shown in panel F, but small particle technical replicates (n=3 per FASS sample) were collapsed.

(I) Same as panel H, but only FASS samples are included in the PCA. See **Supplementary File 6** for complete summary of *DESeq2* testing.

(J) <u>*Upper and lower left:*</u> Volcano plots are derived from the *DESeq2* LRT, with the indicated terms removed from the following two-factor GLM: ~ *genotype* + *fraction* + *genotype:fraction*. <u>*Lower right:*</u> Number of differentially expressed genes (DEGs, FDR < 0.05) from the *DESeq2* LRT test for the indicated factors, related to panel A. See **Supplementary File 8** for complete summary of *DESeq2* testing.

(K) Total UMIs for Input and IP samples from striatal synaptosome RiboTag IPs (n = 12 each genotype for Input, n = 4 each genotype for IP). ns indicates p > 0.05, Welch's unequal variance t-test.

(L-M) log2 fold change vs. abundance (MA) plot for FASS-enriched or FASS-depleted genes shown in **Figure 3G.** Log_2(GenoCrePos.FractionIP) represents the difference in the *fraction* effect between genotypes: { Cre-positive log_2FC(IP/Input) – Cre-negative log_2FC(IP/Input) }.

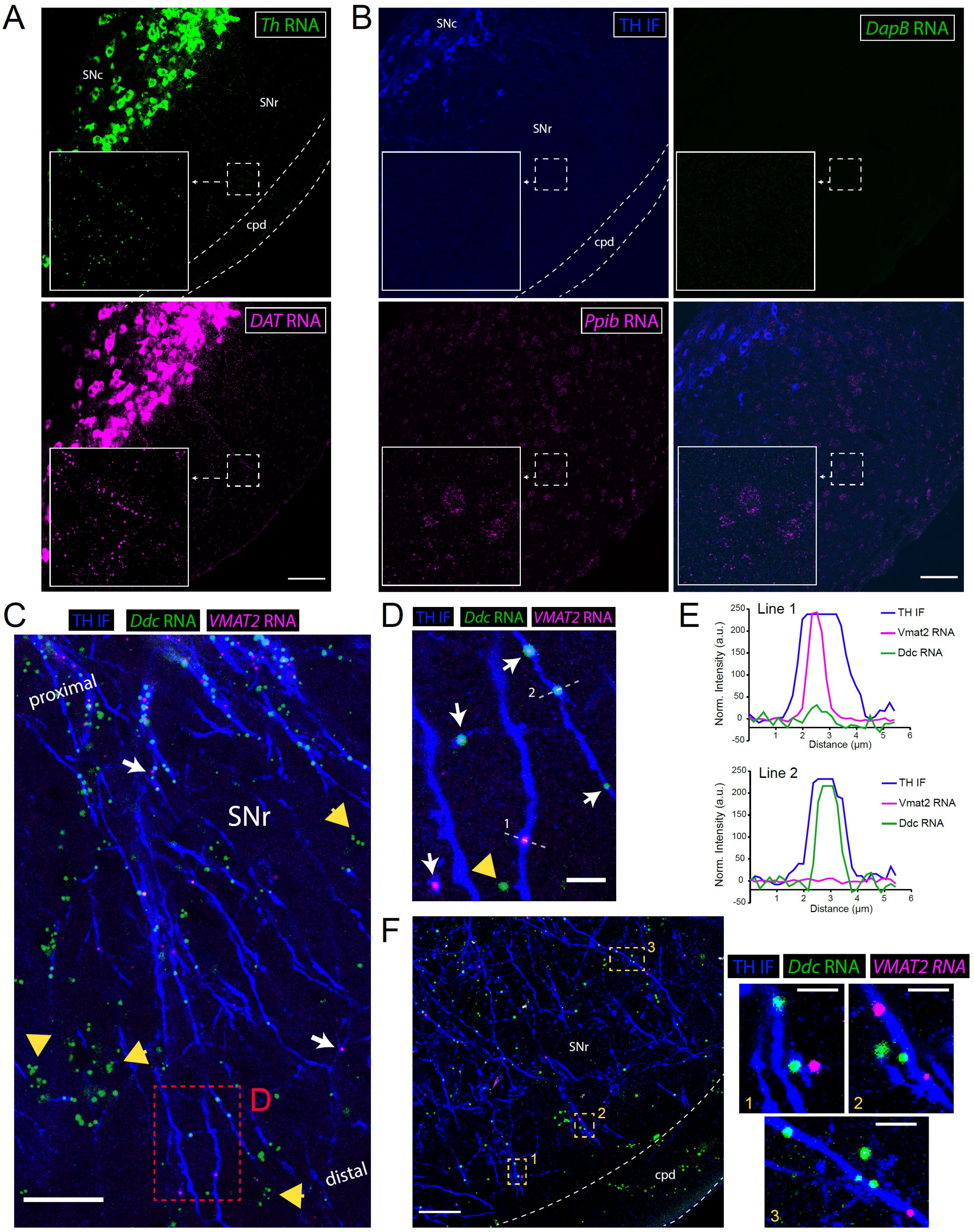

A
Th RNA
SNc
SNr
cpd
DAT RNA
B
SNc
TH IF
DapB RNA
SNr
cpd
Ppib RNA
C
TH IF
Ddc RNA
VMAT2 RNA
proximal
SNr
D
distal
D
TH IF
Ddc RNA
VMAT2 RNA
2
1
E
Line 1
TH IF
Vmat2 RNA
Ddc RNA
Norm. Intensity (a.u.)
Distance (µm)
Line 2
TH IF
Vmat2 RNA
Ddc RNA
Norm. Intensity (a.u.)
Distance (µm)
F
3
SNr
2
1
cpd
TH IF
Ddc RNA
VMAT2 RNA
1
2
3

Figure S3: Midbrain FISH for dopaminergic and control mRNAs, *Ddc* and *Slc18a2/Vmat2* mRNA in TH^{+} SNr dendrites, Related to Figure 4

(A) Multicolor fluorescence *in situ* hybridization (FISH, RNAScope assay) for *Th* and *Slc6a3/DAT* mRNA in the substantia nigra. Scale bar, 100 µm.

(B) TH immunostaining combined with multicolor FISH for the negative control (bacterial) mRNA *DapB* and positive control mRNA *Ppib*. Scale bar, 100 µm.

(C) TH immunostaining combined with multicolor FISH for *Ddc* and *Slc18a2/Vmat2* mRNA in the SNr. Yellow large arrowheads indicate clusters of *Ddc* mRNA outside of TH+ dendrites. White arrows indicate *Slc18a2/Vmat2* mRNA within TH^{+} dendrites. Red dashed lines indicate the inset in panel D. Scale bar, 25 µm.

(D) Inset corresponding to red dashed lines in panel C. Yellow large arrowhead indicates a *Ddc* mRNA puncta outside of TH+ dendrites. White arrows indicate *Ddc* and *Slc18a2/Vmat2* mRNA within TH^{+} dendrites. White dashed lines correspond to the intensity profiles shown below in panel E. Scale bar, 5 µm.

(E) Intensity profiles for all three fluorescent channels, corresponding to the lines indicated above in panel D.

(F) *Left:* TH immunostaining combined with multicolor FISH for *Ddc* and *Slc18a2/Vmat2* mRNA in the distal SNr near the cerebral peduncle (cpd). Yellow dashed lines indicate the insets shown on the right. Scale bar, 25 µm. *Right:* Insets of *Ddc* and *Slc18a2/Vmat2* mRNA in TH^{+} dendrites. Scale bars, 5 µm.

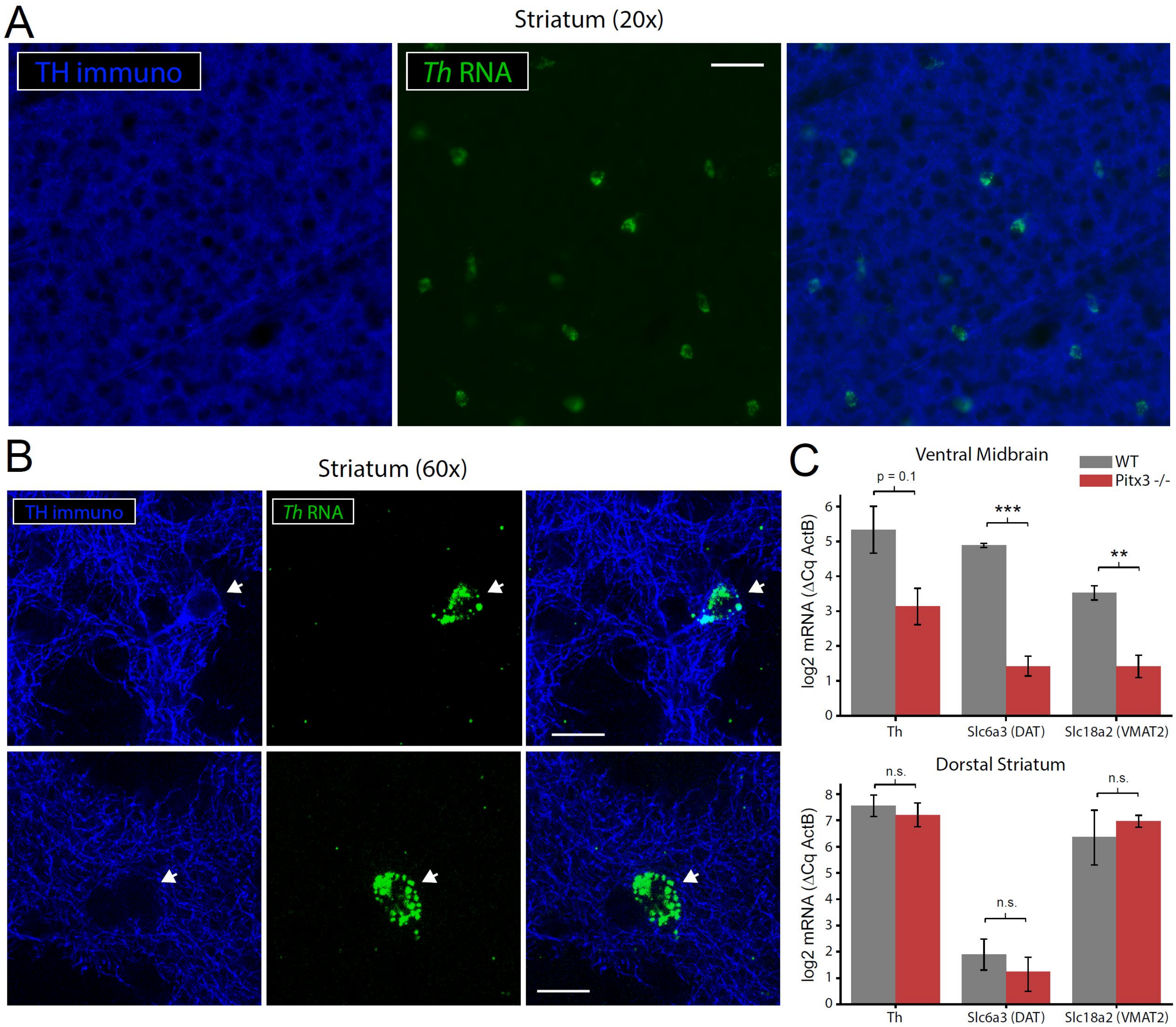

Figure S4: *Th* mRNA$^+$ striatal neurons, not dopaminergic axons, are the source of *Th* mRNA in the striatum, Related to Figure 4

(A) TH immunostaining combined with FISH for *Th* mRNA in the striatum. Scale bar, 50 µm.

(B) TH immunostaining combined with FISH for *Th* mRNA in the striatum. Occasionally, some *Th* mRNA$^+$ neurons also display TH immunoreactivity (*upper*). Typically, they do not (*lower*). Scale bars, 15 µm.

(C) qRT-PCR of the indicated dopaminergic mRNAs in the Ventral Midbrain (*upper*) or dorsal striatum (*lower*) of Wildtype (n=3) and Pitx3-/- mice (n=4). ** indicates $p < 0.01$, *** indicates $p < 0.001$, Welch's unequal variance t-test.

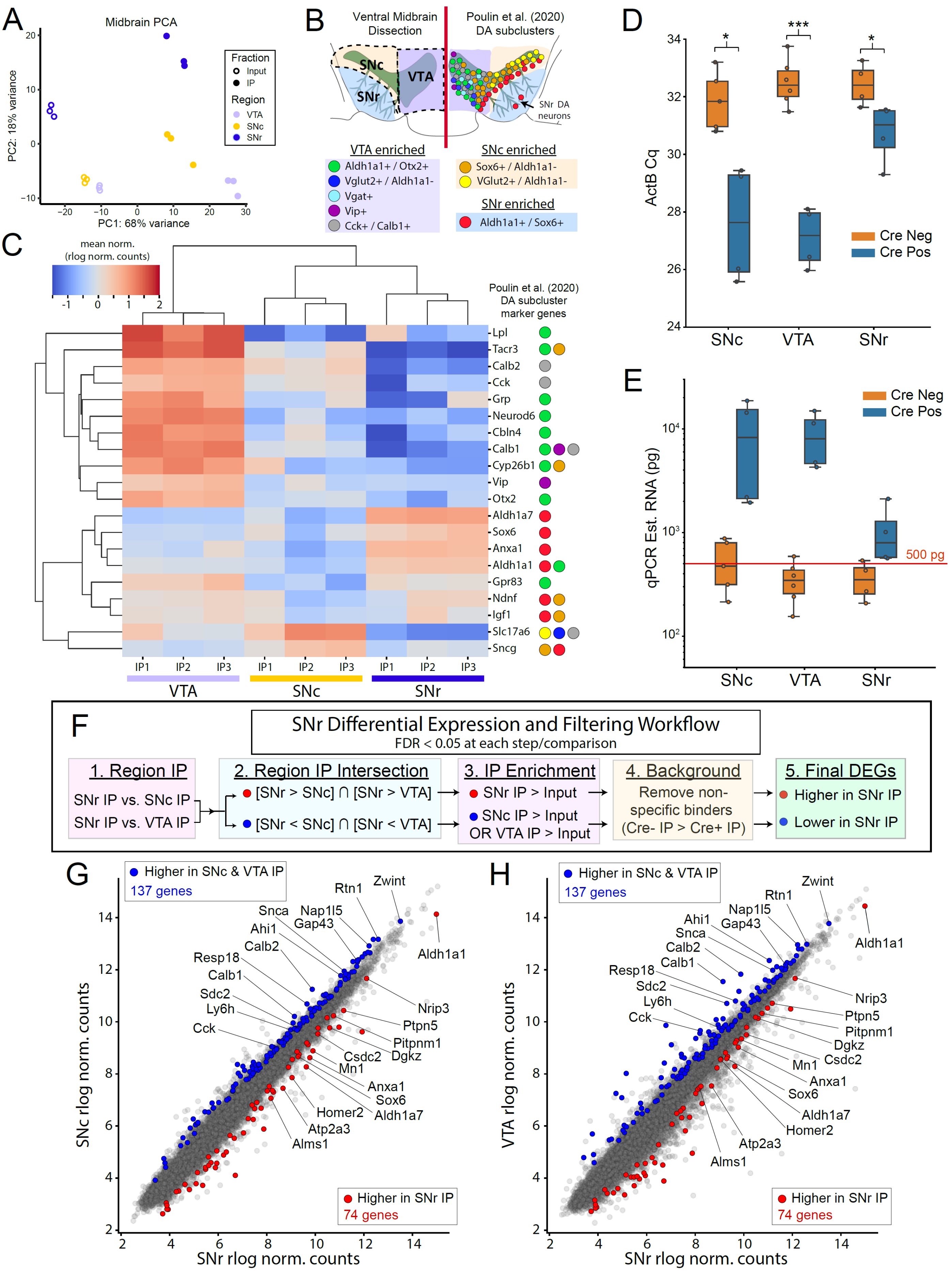
A
Midbrain PCA
PC2: 18% variance
PC1: 68% variance
Fraction
Input
IP
Region
VTA
SNc
SNr
B
Ventral Midbrain Dissection
Poulin et al. (2020) DA subclusters
SNc
VTA
SNr
SNr DA neurons
VTA enriched
Aldh1a1+ / Otx2+
Vglut2+ / Aldh1a1-
Vgat+
Vip+
Cck+ / Calb1+
SNc enriched
Sox6+ / Aldh1a1-
VGlut2+ / Aldh1a1-
SNr enriched
Aldh1a1+ / Sox6+
C
mean norm. (rlog norm. counts)
Poulin et al. (2020) DA subcluster marker genes
Lpl
Tacr3
Calb2
Cck
Grp
Neurod6
Cbln4
Calb1
Cyp26b1
Vip
Otx2
Aldh1a7
Sox6
Anxa1
Aldh1a1
Gpr83
Ndnf
Igf1
Slc17a6
Sncg
IP1 IP2 IP3 IP1 IP2 IP3 IP1 IP2 IP3
VTA
SNc
SNr
D
ActB Cq
Cre Neg
Cre Pos
SNc
VTA
SNr
E
qPCR Est. RNA (pg)
500 pg
Cre Neg
Cre Pos
SNc
VTA
SNr
F
SNr Differential Expression and Filtering Workflow
FDR < 0.05 at each step/comparison
1. Region IP
SNr IP vs. SNc IP
SNr IP vs. VTA IP
2. Region IP Intersection
[SNr > SNc] ∩ [SNr > VTA]
[SNr < SNc] ∩ [SNr < VTA]
3. IP Enrichment
SNr IP > Input
SNc IP > Input OR VTA IP > Input
4. Background
Remove non-specific binders (Cre- IP > Cre+ IP)
5. Final DEGs
Higher in SNr IP
Lower in SNr IP
G
Higher in SNc & VTA IP
137 genes
Higher in SNr IP
74 genes
SNc rlog norm. counts
SNr rlog norm. counts
Rtn1
Zwint
Snca
Nap1l5
Gap43
Ahi1
Calb2
Resp18
Calb1
Sdc2
Ly6h
Cck
Aldh1a1
Nrip3
Ptpn5
Pitpnm1
Dgkz
Csdc2
Mn1
Anxa1
Sox6
Aldh1a7
Homer2
Atp2a3
Alms1
H
Higher in SNc & VTA IP
137 genes
Higher in SNr IP
74 genes
VTA rlog norm. counts
SNr rlog norm. counts
Zwint
Rtn1
Nap1l5
Gap43
Ahi1
Snca
Calb2
Calb1
Resp18
Sdc2
Ly6h
Cck
Aldh1a1
Nrip3
Ptpn5
Pitpnm1
Dgkz
Csdc2
Mn1
Anxa1
Sox6
Aldh1a7
Homer2
Atp2a3
Alms1

Figure S5: RiboTag IP from midbrain dissections recapitulates mDA neuronal heterogeneity, qRT-PCR estimated yield of midbrain RiboTag IPs, SNr RiboTag IP Filtering, and Enrichment of *Aldh1a1*$^{+}$/*Sox6*$^{+}$ mDA neuronal markers, Related to Figure 5

(A) PCA of RiboTag IP and Input samples from VTA, SNc, and SNr dissections.

(B) Schematic depicting anatomical dissections and the anatomical distribution of mDA neuronal clusters described by Poulin et al. (2020).

(C) Clustered heatmap of *DESeq2 rlog* normalized counts, mean-normalized within each gene, for the indicated VTA, SNc, and SNr RiboTag IPs (n=3 each). The twenty genes shown are strong markers for specific mDA neuronal clusters as shown in panel B and in Poulin et al. (2020).

(D) beta-Actin (ActB) Cq values for Cre-negative and Cre-positive RiboTag IPs in the indicated regions (n=4 each region/genotype). * indicates $p < 0.05$, *** indicates $p < 0.001$, Welch's unequal variance t-test.

(E) Estimated total RNA (picograms) based on ActB Cq values. Red line indicates 500 picograms.

(F) Schematic depicting filtering and comparison of SNr RiboTag IP to VTA and SNc RiboTag IPs (FDR < 0.05 at each step). First, DEGs in SNr vs. SNc and in SNr vs. VTA comparisons are identified. Second, the intersection of SNr-enriched or SNr-depleted genes (relative to SNc/VTA) from these two DEG lists is retained. Third, only genes enriched in SNr IP vs. Input or SNc/VTA IP vs. Input comparisons are retained. Fourth, genes that are significantly higher in Cre-negative IP samples compared to Cre-positive IP samples are removed (non-specific binders). The final list of DEGs includes genes enriched (red) or depleted (blue) in SNr RiboTag IPs relative to SNc and VTA RiboTag IPs. See **Supplementary File 9** for complete summary of DEGs and filtering.

(G-H) Log-log plots depicting the average *DESeq2 rlog* normalized counts for SNr RiboTag IPs (x-axis) and SNc (*left*) or VTA (*right*) RiboTag IPs (y-axis). DEGs corresponding to panel F are labeled in blue or red, with select genes labeled.

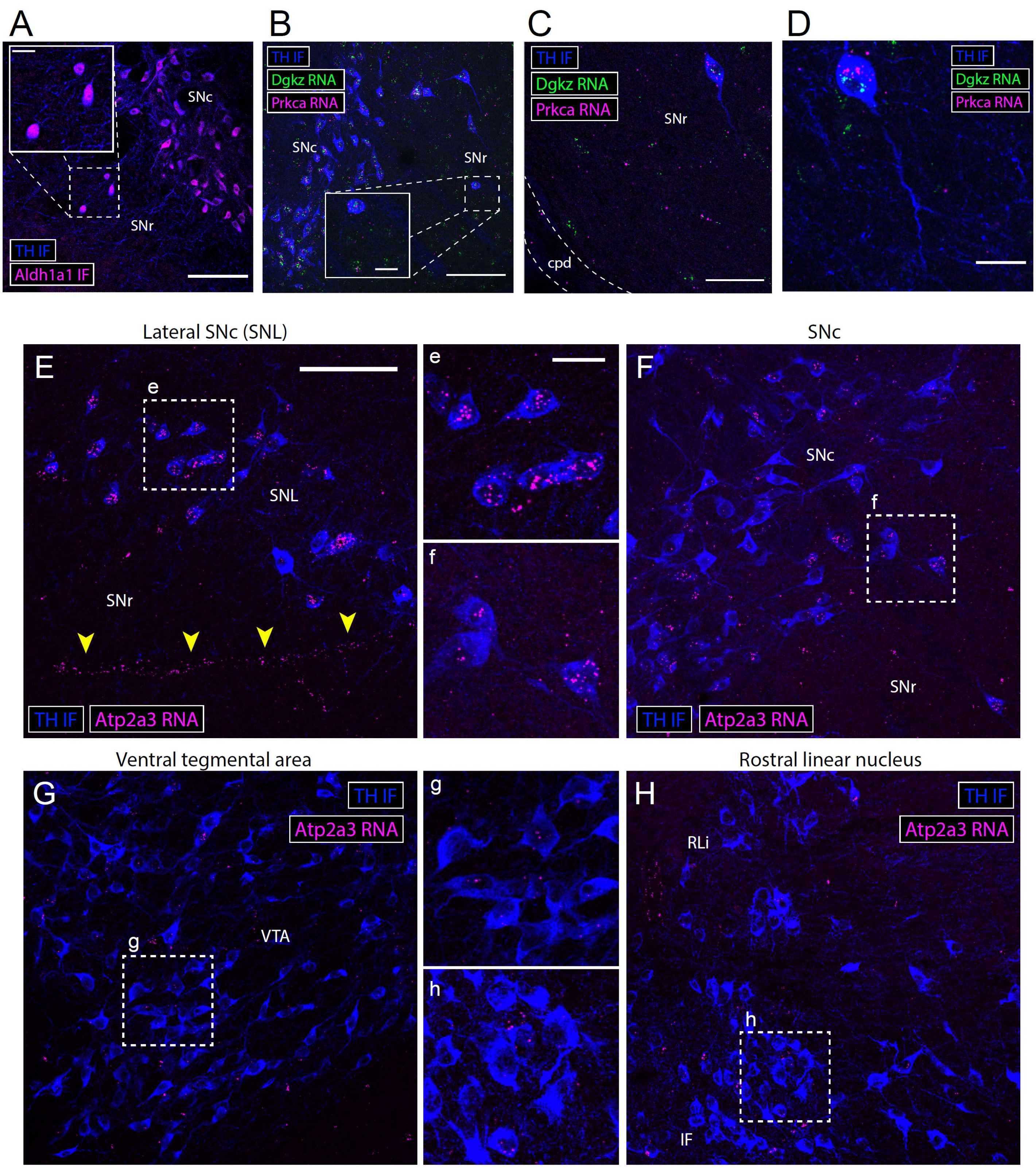

Figure S6: SNr RiboTag IP-enriched mRNAs localized within SNr mDA somata and heterogeneous expression of *Atp2a3* (SERCA3) in mDA neurons, Related to Figure 5

(A) Immunostaining for TH and Aldh1a1 reveals Aldh1a1^{+} mDA neurons within the SNr. Scale bar, 100 µm. Inset scale bar, 20 µm.

(B-D) TH immunostaining combined with multicolor FISH for *Dgkz* and *Prkca* mRNA in the proximal and distal SNr. Both of these SNr RiboTag IP-enriched mRNAs are localized within the soma of mDA neurons in the SNr,

and are not distributed in dopaminergic dendrites. **(B)** Scale bar, 100 μm. Inset scale bar, 20 μm. **(C)** Scale bar, 50 μm. **(D)** Scale bar, 20 μm.

(E-H) TH immunostaining combined with FISH for *Atp2a3/SERCA3* mRNA in the indicated regions. Dashed white lines indicate the insets shown in the center (e-h). Yellow arrowheads in **(E)** indicate prominent labeling of blood vessels, likely within endothelial cells which are known to express SERCA3. Scale bar, 100 μm. Inset scale bar, 25 μm. See **Figure 5G** for quantification within each region.

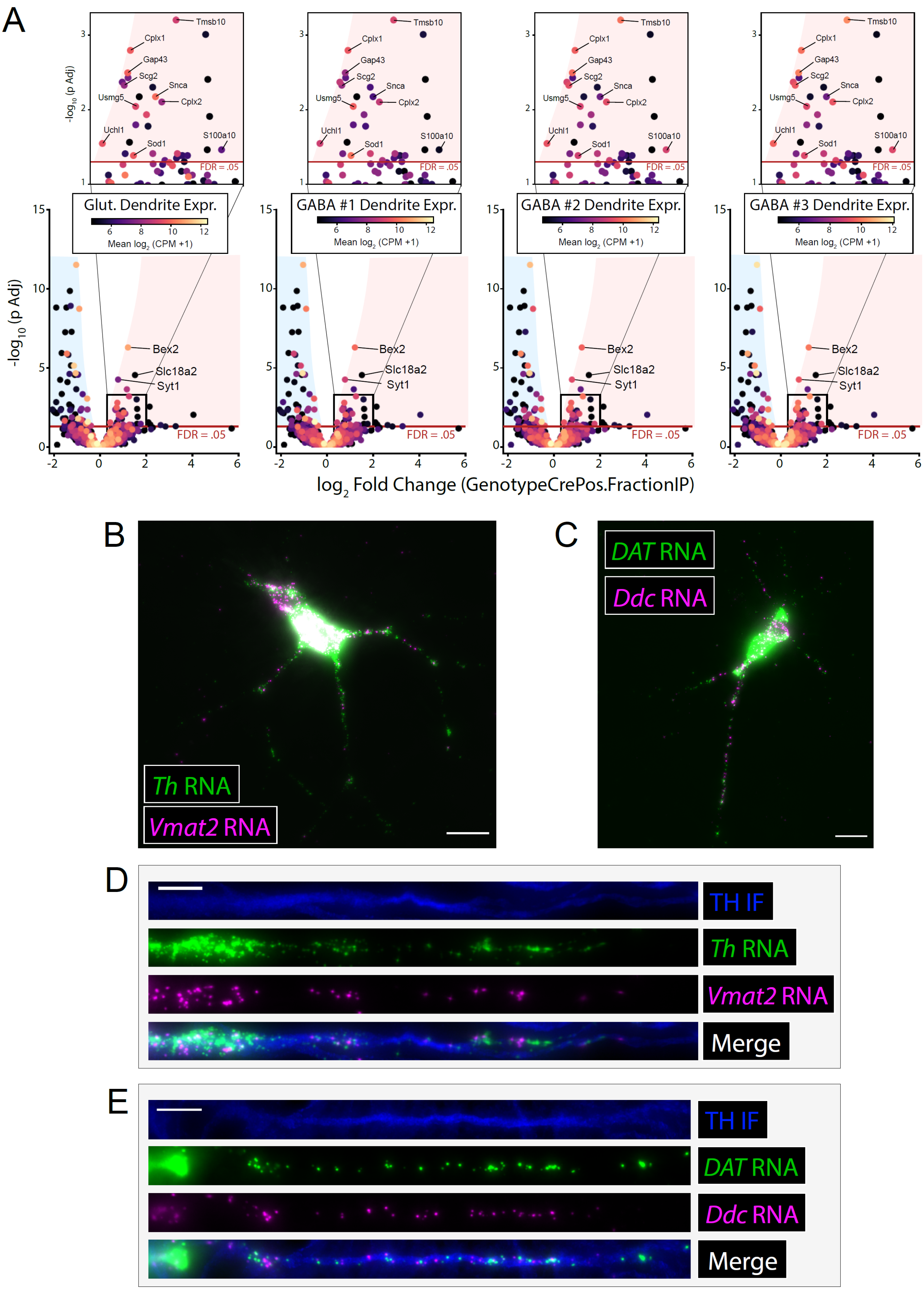
A
Glut. Dendrite Expr.
GABA #1 Dendrite Expr.
GABA #2 Dendrite Expr.
GABA #3 Dendrite Expr.
Mean log2 (CPM +1)
-log10 (p Adj)
Tmsb10
Cplx1
Gap43
Scg2
Snca
Usmg5
Cplx2
Uchl1
Sod1
S100a10
FDR = .05
Bex2
Slc18a2
Syt1
log2 Fold Change (GenotypeCrePos.FractionIP)
B
Th RNA
Vmat2 RNA
C
DAT RNA
Ddc RNA
D
TH IF
Th RNA
Vmat2 RNA
Merge
E
TH IF
DAT RNA
Ddc RNA
Merge

Figure S7: mRNAs encoding presynaptic proteins are also present in hippocampal dendrites, and dendritic localization of dopaminergic mRNAs in cultured mDA neurons, Related to Figure 6

(A) Volcano plots showing the Cre-dependent log2 fold change in IP vs. Input (Cre-positive – Cre-negative) for midbrain synaptosomal RiboTag IPs (same as **Figure 6E**) with genes colored by their expression level in the dendrites of four subsets of hippocampal neurons (Perez et al., 2021).

(B-C) Multicolor FISH for the indicated dopaminergic mRNAs in cultured mDA neurons. Scale bars, 20 μm.

(D-E) TH immunostaining combined with multicolor FISH for the indicated dopaminergic mRNAs in cultured mDA neurons. Straightened dendritic segments are displayed. Scale bars, 10 μm.
